# Biochar improves the nutrient cycle in sandy-textured soils and increases crop yield: a systematic review

**DOI:** 10.1186/s13750-024-00326-5

**Published:** 2024-02-22

**Authors:** Madina Bekchanova, Luca Campion, Stephan Bruns, Tom Kuppens, Johannes Lehmann, Marijke Jozefczak, Ann Cuypers, Robert Malina

**Affiliations:** 1 Centre for Environmental Sciences, Research Group Environmental Economics, UHasselt—Hasselt University, Agoralaan Gebouw D, 3590 Diepenbeek, Belgium; 2 Centre for Environmental Sciences, Research Group Environmental Biology, UHasselt—Hasselt University, Agoralaan Gebouw D, 3590 Diepenbeek, Belgium; 3 Vrije Universiteit Brussel, Multidisciplinary Institute for Teacher Education (MILO), Pleinlaan 9, 1050 Brussels, Belgium; 4 School of Integrative Plant Science, College of Agriculture and Life Sciences, Cornell University, Ithaca, NY USA

**Keywords:** Soil properties, Soil ecosystem services, Soil amendment, Soil fertility, Sustainable agriculture, Food crop yield, Biomass production, Meta-analysis

## Abstract

**Background:**

Biochar is a relatively new development in sustainable agricultural management that can be applied to ameliorate degraded and less fertile soils, especially sandy-textured ones, to improve their productivity with respect to crop production through improved nutrient availability. However, as the literature has shown, the response of sandy-textured soils to biochar varies in terms of effect size and direction. Therefore, the present study systematically reviewed the available evidence to synthesize the impact of biochar amendments on aspects of the nutrient cycle of sandy-textured soils.

**Methods:**

Both peer-reviewed and gray literature were searched in English in bibliographic databases, organizational web pages, and Internet search engines. Articles underwent a two-stage screening (title and abstract, and full-text) based on predefined criteria, with consistency checks. Validity assessments were conducted, utilizing specifically designed tools for study validity. Data extraction involved categorizing the various properties of the nutrient cycle into nine main Soil and Plant Properties (SPPs), each of which was studied independently. Nine meta-analyses were performed using a total of 1609 observations derived from 92 articles. Comparing meta-averages with and without correction for publication bias suggests that publication bias plays a minor role in the literature, while some indication for publication bias is found when accounting for heterogeneity by means of meta-regressions.

**Review findings:**

According to the results, soil total and available nitrogen [N], phosphorous [P] and potassium [K], plant nutrient level, and potential cation exchange capacity (CEC) increased by 36% (CI [23%, 50%]), 34% (CI [15%, 57%]), 15% (CI [1%, 31%]), and 18% (CI [3%, 36%), respectively, and N_2_O emission and mineral nutrient leaching decreased by 29% (CI [− 48%, − 3%]) and 38% (CI [− 56%, − 13%). On average, however, biochar had no effect on soil mineral nitrogen and nutrient use efficiency. Publication bias was identified in the response of effective CEC. After corrections for publication bias, the response shifted from 36% to a negative value of − 34% (CI [− 50%, − 14%]). Meta-regression found that the effect modifiers experimental continent, biochar application rate, and soil pH, explain result heterogeneity. Stronger responses came from the continent of South America, higher application rates, and higher pH soils. Overall, biochar is found useful for many SPPs of nutrient cycling of sandy-textured soils, thereby contributing to increased crop yields in such soils.

**Supplementary Information:**

The online version contains supplementary material available at 10.1186/s13750-024-00326-5.

## Introduction

### Background

Sandy-textured soils are marginal soil types for crop production [1] due to their poor fertility resulting from low organic matter and nutrient contents [2, 3], poor aggregate stability, and low water-holding capacity [4]. However, for certain crops, such as onions or asparagus, sandy soils are considered fertile soils [5]. These soils cover 6% of the Earth’s surface [2], and some are being intensively cultivated in many countries to meet the high demand for food [6]. Sandy soils rely heavily on external inputs such as organic and inorganic fertilizers to restore soil productivity by increasing their nutrient level and consequently engaging in agriculture [7]. However, the use of inorganic fertilizer can cause environmental pollution by releasing N_2_O into the atmosphere, increasing NH_3_ volatilization and N leaching [8]. This situation increases the need for environmentally sound technologies that can improve soil nutrient management and fertility.

Biochar, a recent development in agricultural management [9], has attracted attention as an environmentally sound and sustainable soil management method to regulate soil nutrient cycles, reduce nitrogen effluents [10], and increase the fertility of agricultural land [11, 12]. Biochar is usually obtained by pyrolysis of various biomass materials (such as wood and wood-related residues, manure and litter, peat, organic waste, and agricultural waste) [13] at different production conditions [14]. As a soil amendment, biochar can mitigate soil N_2_O emissions [15], reduce NH_3_ volatilization in soil [16], and increase soil N retention by reducing the need for fertilizer [17–19]. Furthermore, recent studies have revealed that biochar can enhance the uptake of N, P, and K by plants [20, 21]. However, other studies have also reported negative effects of biochar associated with increased N leaching due to soil structure deterioration [22, 23], accelerated soil NH_3_ volatilization due to raised soil pH, and increased soil N_2_O emissions through enhanced nitrification [24]. These contradictory results show that the effects of biochar are still ambiguous.

Not all soil types can benefit from biochar application to the same extent [25]. For example, sandy-textured soils are more likely to benefit from biochar amendments in the long run than other textured soils [25–28]. Even for sandy-textured soils, the effects of biochar addition can differ based on the interaction between biochar characteristics and the properties of sandy-textured soils [29]. Soil nutrient availability after biochar application essentially depends on the biochar characteristics and biochar application rate [30]. Some primary studies reported higher levels of nutrients in biochars obtained from manure and biosolids than those derived from straw- and wood-based feedstocks [31, 32], resulting in increased nutrient availability, extractable P, microbial biomass N, and reduced NO_3_^−^ leaching of sandy-textured soils [28, 33]. However, some other studies have shown that the same biochar types have no effect on nutrient availability [36, 41]. Review studies have highlighted that a higher biochar application rate might increase soil NH_3_ volatilization and the magnitude of soil N_2_O emissions [8, 30]. In contrast, Shakoor, Shahzad [34] found that the biochar application rate had no effect on N_2_O emissions. Variations in the effect of biochar on soil nutrients can also be caused by pyrolysis temperature [35–37].

The existing heterogeneity in results in the literature on the size and magnitude of biochar’s impact on nutrient cycling in sandy-textured soils highlights the need for a systematic review to synthesize and better comprehend the underlying causes. Although three previous studies [8, 38, 39] have investigated similar aspects, our study brings forward new contributions both in content and methodology, complementing existing studies. For example, these reviews focused on biochar’s impact on the soil nutrient cycle, providing state-of-the-art insights. In contrast, our research is grounded in a systematic review, strictly following Collaboration for Environmental Evidence (CEE) guidelines at every stage [40]. The review study conducted by Zhang, Jing [38] mainly addressed the biochar effect on soil microbial activity and nutrient uptake in field studies, while our study includes different experimental designs such as field, greenhouse, and laboratory. Studies conducted by Biederman and Harpole [39] and Liu, Zhang [8] concentrated primarily on soil nutrient cycling (in general) following biochar application, in contrast, ours exclusively investigates changes in sandy-textured soils, covering a broad spectrum of nutrient cycling components in such soils. Methodologically, we introduce an in-depth analysis of publication bias, which is often overlooked in ecological reviews [41]. Since CEE guidelines recommend updates every 5 years [40], our study could serve as a valuable update to previously published two review studies [8, 39]. This review helps to reduce uncertainty and enhances clarity for policymakers about the effect of biochar on the soil nutrient cycle. They can use the results of this review as a roadmap to facilitate policy recommendations for biochar applications as a soil amendment. The results could be valuable to biochar producers, farmers, and other stakeholders that are interested in improving soil conditions through biochar application. Our review also discerned research gaps in current studies and provides a roadmap for further research.

### Stakeholder engagement

A systematic review of biochar’s effect on soil ecosystem services was first brought forward in a meeting of the BASTA project, funded by the Research Foundation Flanders, in December 2019 (BASTA stands for “*Biochar’s added value in sustainable land use with targeted applications*”) [42]. The main aim of that project was the production of biochar from various residual biomasses and their application in different agricultural settings (composting, anaerobic digestion, manure storage, growing media, and open field). Stakeholders of the project (academia, biochar producers, research institutes, agencies, policymakers) are aware of the lack of synthesis on biochar’s soil ecosystem services and they support the systematic review.

The advisory committee, which was set up during the development of the protocol, was interested in various soil ecosystem services such as the water cycle, the nutrient cycle, climate regulation, and biomass/crop production. The advisory committee also contributed to the creation of search terms and search databases. Because the search terms and databases were comprehensive, the number of results retrieved became too vast to fit within the applicable time and money constraints. Hence, in the full-text screening phase, the advisory committee proposed focusing only on the nutrient cycle. This choice was based on its importance for the BASTA project. All deviations from the protocol are explained explicitly in each subsection where the deviations occurred.

### Review objective

The primary focus of this review was to systematically review and synthesize studies of the effect of biochar on the nutrient cycle of sandy-textured soils. To this end, we formulated the following research question: *What is the direction and magnitude of biochar’s impact on nutrient cycling provided by sandy-textured soils?* The research question components were structured based on the PICO model: population, intervention, comparator, and outcome. The *population* in this study consists of sandy-textured soil types. The *intervention* is the soil amendment using biochar, where the control of no biochar amendment serves as the *comparator*. Finally, the *outcome* is a change in sandy-textured soils’ nutrient cycle, which was measured by comparing the treatment (with biochar) to the control (without biochar). To fully understand the potential heterogeneity in the impact of biochar on SPPs, we also examined potential effect modifiers as explained in sub-section *Potential Effect Modifiers and Reasons for Heterogeneity*.

In this review, we defined sandy-textured soils as soils consisting of at least 50 percent sand, using the soil texture categorization by the United States Department of Agriculture (Table 1) [43]. Sandy-textured soils were chosen as the BASTA project will conduct experiments on this type of soil and fill the current research gap. Furthermore, our analysis focused exclusively on biochar experiments conducted on the topsoil layer (0–0.3 m soil depth) to examine the impact on this uppermost layer of soil. Hence, the scope of this study is limited to reviewing the changes in the topsoil nutrient cycle following biochar application. The results of this review clarify the interaction between biochar and sandy-textured soils and its effect on the nutrient cycle, which can be beneficial for further analysis of soil nutrients.

**Table 1:** Soil types chosen for the study

Common names of soils	Sand (%)	Silt (%)	Clay (%)	Textural class
Sandy soils (coarse-textured)	86–100	0–14	0–10	Sand
	70–86	0–30	0–15	Loamy sand
Loamy soils (moderately coarse-textured)	50–70	0–50	0–20	Sandy loam
Loamy soils (moderately fine-textured)	45–80	0–28	20–35	Sandy clay loam

## Methods

Based on our prior protocol [44], the guidelines of the Collaboration for Environmental Evidence [40], and the ROSES reporting standards (Additional file 1) [45], we used a systematic review, including meta-analyses, to synthesize the existing evidence base on the effects of biochar characteristics on sandy-textured soils’ nutrient cycle. We also identify the factors that influence the effect of biochar on the nutrient cycle of sandy-textured soils (such as experimental design, geographic location, climate type, duration of the experiment, soil type, soil depth, and soil treatment before biochar).

### Deviation from protocol

**Fig. 1:**
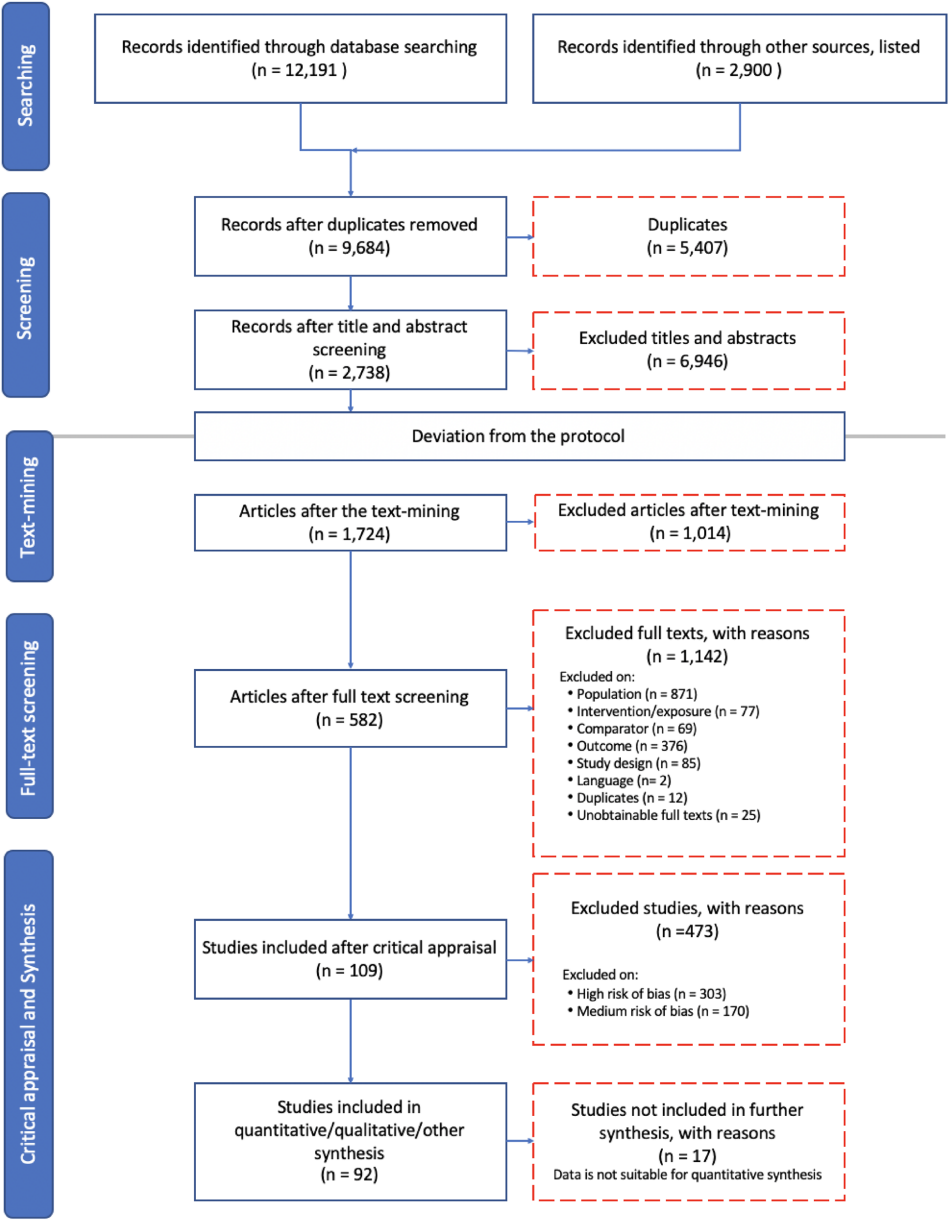
ROSES flow diagram [73], showing the search results, screening processes, critical appraisal, and the final number of articles included in the systematic review. Blue indicates that articles have gone to the next stage; red dotted lines imply that articles/studies have been removed at that stage

As mentioned in the Stakeholder Engagement section, we focused only on one ecosystem service—that is, the nutrient cycle—instead of four. Considering the large number of articles (> 2,500) to be screened during the full-text screening (Fig. 1), it was decided—after consultation with the co-authors and advisory committee—to reduce the number of papers by focusing only on the nutrient cycle instead of four ecosystem services. Text-mining made this possible (see Screening for more information). The research question was changed accordingly. Since these adjustments were made after the first phase of the screening process, we did not make any changes to the search strings. Instead, we restricted the second screening stage to keywords related to the nutrient cycle based on the relevant search strings. Deviations from the protocol are explicitly elaborated under the specific sections (see “Searching for Articles”, “Screening”, “Eligibility”, “Study validity assessment”, and “Data Synthesis and”).

### Searching for articles

#### Search string

Topic searches were used that searched by title—abstract—keywords (TITLE—ABS- KEY). Three of the four PICO components (population, intervention, and outcome) were combined to build search terms. Within each component, search terms were combined using ‘OR’ operators. To combine the three PICO components, ‘AND’ operators were used. Truncation characters (* and $) were used to make the search more expansive. To construct the search string (Table 2), an initial scoping exercise was carried out on the Web of Science (WoS) ‘Core collection’ database, which was complemented with related synonyms by the advisory committee. The institutional subscription of UHasselt was used to collect the search results.

**Table 2:** Search terms (formatted in Web of Science)

Intervention	Population	Outcome
(TS = (biochar* OR "biochar amendment$" OR agrichar OR "black carbon$" OR "biochar-mediated" OR charcoal OR "biochar field study"))	(TS = ("hydrological control*" OR "hydraulic properties*" OR "hydrologic* cycle" OR “water retention” OR “clean water” OR “hydrological control” OR “water leak*” OR “water leach*” OR "water availabl*" OR "soil water availability" OR "readily available water" OR evaporation OR "water runoff" OR "plant available water" OR "WHC" OR "soil humidity" OR "field capacity" OR "nutrient loss" OR "soil moisture" OR "water holding capacity" OR "water content" OR "water infiltration" OR "water cycle" OR "nutrient availabl*" OR "available nutrient$" OR nutrient$ OR "nutrient cycle" OR "nitrogen availabil*" OR "nutrient exchange capacit*" OR "available nitrogen" OR NPK OR "nutrient leak*" OR "nutrient leach*" OR "soil nutrients" OR "available phosphorus" OR "phosphorus availabil*" OR "available potassium" OR "potassium availabil*" OR "climate regula*" OR "climate mitigation" OR "climate change mitigation" OR "climate chang*" OR "global warming" OR “carbon sequestration” OR "soil carbon sequestration" OR “carbon capture and storage” OR "emission$" OR "CCS" OR “ammonia emission$*” OR “methane emission$*” OR "nitrate emission$" OR "nitrous-oxide emission$" OR "$${{\text{NH}}}_{4}$$" OR "$${{\text{NO}}}_{3}$$" OR "$${{\text{CH}}}_{4}$$" OR "$${{\text{N}}}_{2}{\text{O}}$$" OR "$${{\text{CO}}}_{2}$$" OR "greenhouse gas emission$" OR "crop growth" OR "crop yield" OR "crop production" OR "biomass growth" OR "biomass yield" OR "biomass production" OR "raw material" OR "plant growth" OR "field" OR yield OR "increase" OR "improve*" OR "decrease" OR "mitiga*" OR "growth" OR "alleviat*" OR "effect$" OR "impact"))	(TS = ("sand* soil$" OR "sandy soil" OR “sand* loamy soil$” OR “loamy sandy soil” OR “contaminated* soil$” OR "heavy metal* soil$" OR "sandy loam soil" OR "sandy loam" OR ultisol OR "agricultural soil" OR "loamy sand" OR "sandy clay loam" OR "cropping system" OR “sandy calcareous soil”))
Search formula = Intervention + Population + Outcome		

* and $ mean truncation characters

The search terms were designed to retrieve all publications on biochar’s effect on the nutrient cycle, water cycle, climate regulation, and crop/biomass yield of sandy-textured and contaminated sandy soils. No changes were made to the search terms, as the restriction to the scope of the nutrient cycle occurred after the first screening of the records retrieved using those terms. Both peer-reviewed publications and gray literature (not submitted to peer-reviewed journals) were retrieved to minimize publication bias [46]. The following sources were searched: bibliographic databases (for peer-reviewed publications), organizational/institutional websites, and web-based searches (for gray literature, which can include publications, organizational reports, theses, etc.). The search terms displayed in Table 2 are mostly applicable to bibliographic databases as they have more advanced search functions. When the search terms were applied to organizational websites and Internet-based searches, the search formula and Boolean operators were simplified because some had limited search interfaces. During the protocol development, the comprehensiveness of the search was tested against 15 benchmark articles, and the results demonstrated the adequacy of our search strings as outlined in the previously published protocol [44]. Search strings used in each source were stored and are provided in Additional file 2.

#### Search language

The search was conducted in English and search results in other languages were not considered for this review. As English is the common language within the review team, it provided dual consistency checking in screening, full-text screening, quality appraisal, and data extraction.

#### Bibliographic databases

The following bibliographic databases were used to collect publications:

- Web of Science “Core collection”: https://www.webofknowledge.com
- Scopus: https://www.scopus.com/
- AGRICOLA: https://www.agricola.nal.usda.gov/
- AGRIS: https://www.agris.fao.org/
- ProQuest Environmental Sciences and Pollution Management: https://search.proquest.com/advanced
- EBSCO Open Dissertations: https://biblioboard.com/opendissertations/
- Networked Digital Library of Theses and Dissertations: http://www.ndltd.org/
- Open access theses and dissertations: https://oatd.org/

#### Organizational websites

The following organizational websites were searched to obtain potential additional studies that were not covered by the bibliographic databases. As mentioned above, some organizational websites did not have a very advanced search function. Thus, some manual searches were executed using the search terms defined in Table 2. Furthermore, six of these organization websites returned no results or no related results (Additional file 2).

- International Biochar Initiative: https://biochar-international.org/biochar/
- UK Biochar Research Center: https://www.biochar.ac.uk/research.php?id=10&r=a
- US Biochar Initiative: https://biochar-us.org/biochar-introduction
- European Biochar Certificate: https://www.european-biochar.org/en/home
- Sonoma Biochar Initiative: https://sonomabiocharinitiative.org/
- Israel Biochar Research Network: https://sites.google.com/site/ibrnisraelbiocharnetwork/home
- Biochar for sustainable soils: https://biochar.international/
- Ithaka Institute: http://www.ithaka-institut.org/en/home
- New Zealand Biochar Research Centre: https://www.massey.ac.nz/massey/learning/colleges/college-of-sciences/research/agriculture-environment-research/biochar-research-centre/biochar-research-centre_home.cfm
- Environmental Protection Agency [18]: https://www.epa.gov/
- Research Institute for Organic Agriculture: https://knowledge4policy.ec.europa.eu/organisation/research-institute-organic-agriculture_en
- Research Institute for Agriculture, Fisheries and Food (ILVO, Belgium, Flanders): https://www.ilvo.vlaanderen.be/EN/Home
- Netherlands Organisation for Applied Scientific Research (TNO, The Netherlands): https://www.tno.nl/en/
- Wageningen University & Research (The Netherlands): https://www.wur.nl/en/wageningen-university.htm
- Julius Kühn Institute—Federal Research Centre for Cultivated Plants (Germany): https://www.julius-kuehn.de/en/
- Mercator Research Institute on Global Commons and Climate Change (Germany): https://www.mcc-berlin.net/en/index.html
- Thünen Institute—Federal Research Institute for Rural Areas, Forestry and Fisheries (Germany): https://www.thuenen.de/en/about-us/the-institute/
- Agroscope (Switzerland): https://www.agroscope.admin.ch/agroscope/en/home
- James Hutton Institute (United Kingdom): https://www.hutton.ac.uk/
- Rothamsted Research (United Kingdom): https://www.rothamsted.ac.uk/
- UK Centre for Ecology & Hydrology (United Kingdom): https://www.ceh.ac.uk/

#### Web-based searches

In addition to the bibliographic database and organizational website searches, web-based searches were also conducted. Contrary to what is stated in the protocol, we only used Google Scholar, mainly because it yielded very similar results to Google. We performed this test by randomly selecting 100 results from each dataset and comparing them against each other. To achieve this, we initially exported the search results to an Excel sheet. We used the Excel randomization function, assigning a 9-digit random number between 0 and 1 to each cell. Subsequently, we chose 100 lowest-ranking results that were randomly selected. Google Scholar returned many results that were related and unrelated to the review objective; therefore, following [46] and [47], we only downloaded the first 1000 relevant results using Publish or Perish software, as stated in the protocol. The software assisted in retrieving relevant articles and adding them to the reference list in Endnote. Citations or patents were not considered in Google Scholar. Finally, we did not include the reference lists of relevant articles for the additional searches due to the large number of hits retrieved from all sources.

#### Search record database

Endnote X9 was used to import the search results. When it was not possible to import a certain document into the software, a record was generated manually. After finalizing all searches, the reference files were merged and checked for duplicates. Before removing any of the duplicates, they were rechecked and compared with regard to title, abstract, and year of publication. Once all duplicates were removed, the final version was checked with Endnote’s deduplication tool.

### Article screening and study eligibility criteria

#### Screening process

The screening processes involved two steps, both of which were performed by two reviewers independently. In the first step, the results were filtered by title and abstract. All titles and abstracts were double-screened. Before the actual screening began, both reviewers screened 100 randomly selected articles from the search results to ensure consistency. If a reviewer had any doubts about excluding a paper, it was marked and later discussed with the other reviewer to come to an agreement. The involvement of an adjudicating reviewer was not needed, since the reviewers could reach a consensus. The level of agreement with respect to consistency was verified using the Kappa statistic [47]. All disagreements were discussed in detail between reviewers prior to starting the actual screening.

The second step involved a review of the full text of the remaining articles. This is the stage at which we deviated from the protocol, as the vast number of results conflicted with our time and budget constraints. Instead of reviewing all results remaining after the first stage, we only screened results that were related to the nutrient cycle of sandy–textured soils. To implement this, we employed a text-mining method with the keywords related to the nutrient cycle (Additional file 3) using *tidyverse, magrittr, and pdftools packages of R version 3.3.0* [48]*.* Before applying text-mining, we determined the cut-off level of eight counts of nutrient cycle keywords by manually screening 150 randomly selected papers that remained after the first stage of screening and then comparing them to the text-mining results for consistency. After the text-mining, papers that did not yield eight counts of nutrient cycle keywords were excluded, reducing the number of articles by 1014 (Fig. 1). The manual full-text screening of the remaining articles was based on the eligibility criteria provided in the following section, which was slightly different from the previously published protocol [44], as stated in detail in the sub-section of Eligibility Criteria. Articles (co-)authored by one of the reviewers were assigned to another reviewer so that no reviewer had to assess their own work. As in the first phase of screening, consistency checking was performed before screening using 10 percent of the remaining articles. All articles left out at the manual full-text screening stage were recorded, together with an explanation for their omission (Additional file 4).

#### Eligibility criteria

The eligibility criteria were based on the PICO model and are summarized in Table 3.

**Table 3:** Overview of eligibility criteria

Criterion	Included	Excluded	Screening stage I	Screening stage II	Justification
Population	Sandy-textured soils with 50% or more sand in the soil composition	Other soil textures or soil types not given	X	X	Marginal soils, such as sandy-textured soils, are widely used in agricultural processes and seem to benefit from biochar. The heterogeneity in the results of primary studies is the reason why this review focuses on these soils
Intervention	Biochar obtained by pyrolysis and/or gasification of various biomass materials	Biochar made from materials other than biomass or technologies other than pyrolysis and/or gasification	X	X	In accordance with the definition of biochar adopted for this review, the included biochar samples were specifically derived from biomass through pyrolysis and/or gasification processes
Comparator	Control sites or plots without biochar that correspond to intervention sites or plots	Control sites or plots that do not correspond to intervention sites or plots		X	To ensure comparability between comparator and intervention
Outcome	Positive, negative, or no changes in different soil ecosystem processes of the nutrient cycle of sandy-textured soils	Other soil ecosystem processes of sandy-textured soil		X	These soil ecosystem processes of the nutrient cycle were identified as relevant by the advisory committee of this review, being also the partners in the project funding this review (BASTA, Research Foundation – Flanders, grant S000119N)
Study design	Lab (incubation), greenhouse, and field experiments	Review studies		X	Primary study designs provide observations for qualitative and quantitative synthesis
Publication type	Research articles, research reports, dissertations, etc	Review studies	X	X	In order to consistently account for primary data only, review studies were excluded
Language	English	Other languages	X	X	English was the common language within the review team, so it provided dual consistency at all stages of the screening

Screening stage I means screening of the title and abstract, Screening stage II means screening of the full text

##### Population

To establish eligible populations, we excluded studies that conducted experiments on soils not classified as sandy-textured soils (Table 1) and studies that did not focus on the topsoil layer (0–0.3 m). Contrary to the protocol [44], if the soil type upon which an experiment was performed was not defined, it was excluded from this review. Due to the vast number of articles, we were unable to contact every author when the soil type was not specified.

##### Intervention

The eligible intervention was biochar, produced from biomass and used in agriculture as a soil amendment. Some studies have used terms such as charcoal and black carbon as synonyms for biochar; however, in production and application, black carbon and charcoal can differ from biochar. For instance, black carbon can be made by burning fossil fuels as well as biomass [49], while charcoal is mainly produced to provide affordable energy to rural areas [50]. However, we included the terms ‘black carbon’ and ‘charcoal’ in the search terms and checked, at the full-text screening stage, whether they were made from organic feedstocks with the intended application as a soil amendment. We also included the term Agrichar, as it is used in some organizational websites and is closely related to biochar.

##### Comparator

Control sites or plots without any intervention—that is, no biochar added—were used as a comparator. Control sites or plots that were treated with fertilizers, manure, or compost were also included if the intervention plots received the same amount of fertilizers, manure, or compost.

##### Outcomes

The outcomes considered were: positive, negative, or no changes in different components of the nutrient cycle of sandy-textured soils. Changes in major soil nutrients were considered for this study, such as nitrogen (N), phosphorus (P), potassium (K), and other soil mineral processes. Following discussion with the advisory committee, it was decided that the various components of the nutrient cycle should be referred to as soil and plant properties (SPPs) relevant to the nutrient cycle.

##### Study designs

Experimental primary studies, especially laboratory experiments, greenhouse experiments, and field experiments using control and treatment groups, were included in the review. Earlier review studies were accounted for by screening their reference lists.

### Study validity assessment

All studies included after the full-text screening were critically appraised to assess their internal and external validity and to evaluate their suitability for the data synthesis. Studies were appraised for the different types of biases by being categorized as ‘low,’ ‘moderate,’ and ‘high’ risk of bias.

The criteria were created based on the critical appraisal tool of v0.3 developed by CEE [51], and previously published systematic review studies in the journal Environmental Evidence [52] to assess the internal validity of the studies and consider many possible biases: selection bias, performance bias, attrition bias, and reporting bias. In this study, we adapted these frameworks to the research question of our review and deviated from the protocol by making some minor changes to the criteria in Additional file 5: Table S5.1. For example, studies that lacked randomized experiments were classified as a 'high' risk of bias. This classification was made because such studies have the potential to introduce confounding factors into the analysis. This decision was taken after consultation with experts in the field. Studies were summarized as a ‘moderate’ or ‘high’ risk of bias if they were evaluated as ‘moderate’ or ‘high’ risk of bias for at least one of the critical appraisal criteria. Our main reason for excluding studies with a ‘moderate’ risk of bias from the data synthesis was that they would increase the amount of missing data related to effect modifiers, and this might cause sampling bias, limiting the generalizability of the results [53]. Thus, contrary to the protocol, only studies with a ‘low’ risk of bias were used to extract the necessary data for data synthesis [44]. All information related to the critical appraisal (e.g., excluded studies) was recorded in Excel and presented in Additional file 6.

As with screening, consistency checking was also carried out for the critical appraisal. Again, all studies were independently evaluated by the same two appointed reviewers. In case of disagreements regarding any of the criteria, it was discussed between reviewers until a consensus was reached.

### Data coding and extraction strategy

Data were extracted from studies assessed as having a ‘low’ risk of bias. When each study reported estimates for different SPPs or multiple estimates for the same SPPs, all estimates were placed in individual rows with the mean value of the given result. Essential data were extracted based on the data coding table provided in the protocol [44], which was complemented with further soil and biochar data during the data extraction (for example, soil and biochar bulk density, pH, and dry weight) (Additional file 7: Table S7.1). This additional data was necessary to conduct the meta-regression analysis and subsequent calculations, including the conversion of measurement units. Each study’s full text was read, and data were manually extracted and entered into a spreadsheet. If the data were only presented in tables or graphs, they were extracted using WebPlotDigitizer (WPD) [54]. We excluded studies for which the data was impossible to decipher using WPD. As mentioned in the protocol, data extraction was managed by one reviewer (the first author) instead of two. However, prior to conducting the meta-analysis, a third co-author, an expert in the field, rigorously reviewed the extracted data. To guarantee data accuracy and reliability, five random articles were chosen for detailed cross-checking.

#### Potential effect modifiers and reasons for heterogeneity

To better understand possible heterogeneity in the effects found by different studies, possible effect modifiers were retrieved and recorded in the spreadsheet. The list of effect modifiers shown in Table 4 was compiled in consultation with the advisory committee. These effect modifiers were published in the peer-reviewed protocol of this review [44]. We made minor updates on the effect modifiers listed in the protocol with some new moderators (Table 4).

**Table 4:** Potential effect modifiers

Existing modifiers	New modifiers
Experimental country	Soil pH
Experimental design (i.e., greenhouse, lab, field)	Biochar pH
Experimental condition	Biochar application with manure
Climate type	Biochar with compost
Duration of experiment	
Feedstock for biochar	
Biochar carbon content	
Pyrolysis temperature	
Biochar application rate	
Type of soil	

During the data extraction processes, we realized that these new modifiers can have an effect on response variation, so we included them in the list of possible modifiers as well. The effect of these effect modifiers on the variation in the outcome is investigated by means of the meta-regression analysis.

### Data synthesis and presentation

#### Data preparation

Since nutrient cycling in the soil is a complex system, we tried to extract all related measurements and, after discussion with experts, grouped them into nine major SPPs based on the data availability, as shown in Table 5. This decision was taken at the data extraction stage after the protocol was published. We believe that the combination of these nine SPPs provides a reasonable representation of nutrient cycling. However, before combining them, we checked the divergence of the outcomes of different measurement methods. For example, there are different soil phosphorus measurement methods, such as Olsen P and Mehlich 1, 3 extraction, which means that the results can differ greatly, in which case the grouping would lead to inaccurate results. Thus, we analyzed the statistical difference among these methods (Additional file 8) and found that the difference was not statistically significant.

**Table 5:** Grouped major SPPs relevant to the nutrient cycle

Grouped (SPPs)	Soil and plant properties (SPPs)
Soil total NPK	K concentration, K content, N concentration, N content, P concentration, P content, total K, total N, total P
Soil mineral nitrogen	Ammonification rate, available ammonium, available nitrate nitrogen, exchangeable ammonium, extractable ammonium, N mineralization, nitrate nitrogen rate, nitrification, soil ammonium, soil ammonium concentration, soil ammonium content, soil nitrate nitrogen, soil nitric nitrogen
Plant nutrient level	Shoot N, Shoot P, Shoot K, K uptake, P uptake, N uptake, plant N
N_2_O emission	Nitrous oxide emission
Soil NPK availability	Available N, available P, available K, dissolved K, dissolved P, exchangeable K, extractable K, extractable P
Potential CEC	Potential CEC
Effective CEC	Effective CEC
Nutrient use efficiency	Nutrient use efficiency
Mineral N leaching	Cumulative ammonium leaching, cumulative nitrate nitrogen leaching, leached ammonium, leached nitrate nitrogen, nitrate leaching

Soil total NPK is the total content of NPK nutrients, while soil NPK availability is the nutrients in the soil that are readily available to plants. Soil mineral nitrogen includes processes such as nitrification, ammonification, as well as ammonium and nitrate, which are forms of plant-available nitrogen if they are exchangeable or in soil solution. The nutrients (NPK) absorbed by a plant and the nutrient content in the roots and shoots of the plant are considered as plant nutrient level. The difference between the two types of CEC is their measurement methods. For example, effective CEC can be measured at soil pH, whereas potential CEC is usually measured at a pH of 7 by very different methods. Thus, it is not recommended to merge them into one CEC. NUE can be defined as the crop yield or biomass per input unit (that is, fertilizer or biochar), while mineral N leaching is the leaching of ammonium or nitrate to groundwater.

In addition, the results in different units were converted to the same units for each SPP for analysis purposes. Furthermore, some studies have reported biochar application rates in different measurement units (such as kg/ha, kg/pot, or g/kg or in percentage). All were converted into t/ha in order to standardize measurement units. The data were cleaned up before analysis using the *tidyverse package of R version 3.3.0* [48].

#### Descriptive statistics and narrative synthesis

Data from the ‘low’ risk of bias studies were also used for the narrative synthesis and descriptive statistics of evidence. We have made tables and figures to show the number of publications per year and per experimental country, experimental designs, types of experiments (control, treatment), and duration of experiments.

### Quantitative synthesis

#### Effect size and its variance

In addition to narrative synthesis, a quantitative synthesis—that is, a meta-analysis—was performed to assess the effects of biochar on the nutrient cycle of sandy-textured soils. Studies with incomplete or missing information that could not be retrieved were excluded from the meta-analysis. For each SPP (Table 5), we studied the effect of biochar (treatment) compared to no biochar (control). In total, nine meta-analyses were performed. All calculations were carried out using the *metafor package in R version 3.3.0* [48, 55]. For each comparison between biochar and no biochar, we calculated the response ratio (RR), which is the favored method for calculating the effect size in ecological studies [39, 56]:

1 $${\text{RR}}= \frac{\overline{{X}_{b}}}{\overline{{{\text{X}}}_{nb}}}$$

where $\overline{{X}_{b}}$ is the mean value of the soil nutrient cycle with the biochar treatment and $\overline{{{\text{X}}}_{nb}}$ is the mean value of the soil nutrient cycle with the control of no biochar. We use the natural logarithmic transformation, $LnRR=ln\left(RR\right)$, to normalize the data [57]. The biochar treatment is considered to have no effect when the $lnRR=1$, a positive effect when $lnRR>1$, and a negative effect when $lnRR <1$. The variance of LnRR was calculated as follows:

2 $${\text{v}}=\frac{{{\text{SD}}}_{{\text{b}}}^{2}}{({{\text{n}}}_{{\text{b}}}*{{\text{X}}}_{{\text{b}}}^{2})}+ \frac{{{\text{SD}}}_{{\text{nb}}}^{2}}{({{\text{n}}}_{{\text{nb}}}*{{\text{X}}}_{{\text{nb}}}^{2})}$$

where ${SD}_{b}$ and ${SD}_{nb}$ are the standard deviations (SD) of treatment and control, respectively, and ${n}_{b}$ is the sample size of the treatment and ${n}_{nb}$ is the control sample size [57]. Where it was not possible to extract SD, the standard error (SE) was used to calculate SD [41]. According to Lajeunesse [58], Eqs. 1 and 2 are biased when dealing with small to medium sample sizes. Consequently, the following equations, grounded in the second-order Taylor expansion, have been proposed to mitigate these biases [59].

3 $${{\text{LnRR}}}_{2}={\text{ln}}\left(\frac{\overline{{{\text{X}}}_{{\text{b}}}}}{\overline{{{\text{X}}}_{{\text{nb}}}}}\right)+ \frac{1}{2}(\frac{{{\text{CV}}}_{{\text{b}}}^{2}}{{{\text{n}}}_{{\text{b}}}}- \frac{{{\text{CV}}}_{{\text{nb}}}^{2}}{{{\text{n}}}_{{\text{nb}}}})$$

4 $${{\text{v}}({\text{LnRR}})}_{2}=\frac{{{\text{CV}}}_{{\text{b}}}^{2}}{{{\text{n}}}_{{\text{b}}}}+ \frac{{{\text{CV}}}_{{\text{nb}}}^{2}}{{{\text{n}}}_{{\text{nb}}}}+\frac{{{\text{CV}}}_{{\text{b}}}^{4}}{{2{\text{n}}}_{{\text{b}}}^{2}}+ \frac{{{\text{CV}}}_{{\text{nb}}}^{4}}{{2{\text{n}}}_{{\text{nb}}}^{2}}$$

where CV *(sd/m)* is the coefficient of variation. If the SDs were reported then we used Eq. 3 and 4 to calculate effect sizes and variances. However, most studies did not include data on SD and SE. To address this, we used a method known as "missing cases," following the suggestion of Nakagawa, Noble [60], and employed Eqs. 5 and 6. In this approach, we imputed the pooled CV using data from studies that did report SDs.

5 $${{\text{LnRR}}}_{3}={\text{ln}}\left(\frac{\overline{{{\text{X}}}_{{\text{b}}}}}{\overline{{{\text{X}}}_{{\text{nb}}}}}\right)+ \frac{1}{2}\left(\frac{{\left[{\sum }_{i=1}^{k}({n}_{bi}{CV}_{bi}\right)/{\sum }_{i=1}^{k}{n}_{bi}]}^{2}}{{{\text{n}}}_{{\text{b}}}}- \frac{{\left[{\sum }_{i=1}^{k}({n}_{nbi}{CV}_{nbi}\right)/{\sum }_{i=1}^{k}{n}_{nbi}]}^{2}}{{{\text{n}}}_{{\text{nb}}}}\right)$$

6 $${{\text{v}}({\text{LnRR}}}_{3})=\frac{{\left[{\sum }_{i=1}^{k}({n}_{bi}{CV}_{bi}\right)/{\sum }_{i=1}^{k}{n}_{bi}]}^{2}}{{{\text{n}}}_{{\text{b}}}}+ \frac{{\left[{\sum }_{i=1}^{k}({n}_{nbi}{CV}_{nbi}\right)/{\sum }_{i=1}^{k}{n}_{nbi}]}^{2}}{{{\text{n}}}_{{\text{nb}}}}+ \frac{{\left[{\sum }_{i=1}^{k}({n}_{bi}{CV}_{bi}\right)/{\sum }_{i=1}^{k}{n}_{bi}]}^{4}}{{2n}_{b}^{2}}- \frac{{\left[{\sum }_{i=1}^{k}({n}_{nbi}{CV}_{nbi}\right)/{\sum }_{i=1}^{k}{n}_{nbi}]}^{4}}{{2n}_{nb}^{2}}$$

Each study may report multiple effect sizes for the same SPP [61], violating the traditional independency assumption [62]. We applied a multilevel model using the *rma.mv* function in the *metafor R package* to account for the dependency of effect sizes Specifically, we use a three-level model that accounts for the sampling variance of individual effect sizes (level 1), the variance between effect sizes from the same study (level 2), and the variance between studies (level 3) [63] using the following formula:

7 $${y}_{i}= {\beta }_{0}+ {s}_{\left(2\right)ij}+ {u}_{(3)j}+ {\epsilon }_{ij}$$

where ${\beta }_{0}$ is the overall estimate (or meta-analytic mean), ${y}_{ij}$ is the effect size $i$ nested in study $j$, ${s}_{\left(2\right)ij}$ accounts for within-study heterogeneity (level 2), ${u}_{(3)j}$ accounts for between-study heterogeneity on (level 3), and ${\epsilon }_{ij}$ is the corresponding sampling error (level 1). We compared a three-level model with a two-level model using the *anova function* in* R*, where the results showed that the three-level model is a better fit for our data. The model is estimated with multilevel model with random effects (ML-REML), as it provides unbiased estimates of the variance parameters [55]. We further account for dependence due to multiple observations from the same study by clustering standard errors at the level of primary studies [64, 65]. We used inverse variance weighting to assign a greater weight to more precisely estimated studies [57].

We used funnel plots and PET (the precision-effect test) and PEESE (the precision-effect estimate with standard error) tests to check for publication bias, as this method is suggested for ecological studies [41, 66]. Publication bias happens when studies with non-significant or minimal effect sizes (small studies) are unpublished, resulting in a non-normal distribution of LnRR [41, 67]. In the absence of publication bias, all studies would be symmetrically distributed around the pooled effect size within the funnel plot [64]. However, in the presence of publication bias, the funnel plot would be asymmetrical, with smaller studies showing large effect sizes being more likely to be published, while smaller studies without significant or large effects may be missing [64].

PET-PEESE deals with small study effects that are the potential indicators of publication bias. The PET method is based on a regression model where the SE of the study’s effect size is used to correct for publication bias:

8 $${y}_{i}= {\beta }_{0}+ {\beta }_{1}{SE}_{i}+ {s}_{\left(2\right)ij}+ {u}_{(3)j}+ {\epsilon }_{ij}$$

Unlike stated in the formula, we used the square root of the inverse of effective sample size ($\sqrt{1/\widetilde{ {n}_{i}}})$ instead of SE because it is recommended for the studies where the effect size is calculated based on response rate [41]. LnRR includes both treatment and control means in its point estimate, leading to a correlation between these estimates and their sampling standard errors [41], which causes a funnel asymmetry [68]. Therefore, when the effect size is computed in the form of LnRR, it has been proposed to use ‘effective sample size’ as a moderator instead of SE [41] and it also accounts for unbalanced sampling [69]. The square root of the inverse of the effective sample size is given by

9 $$\sqrt{1/\widetilde{ {n}_{i}}}= \sqrt{{n}_{1i}{+ n}_{2i}/{n}_{1i}{n}_{2i}}$$

where ${n}_{1i}$ is the sample size of the control, while ${n}_{2i}$ is the sample size of the treatment. When the true effect size is zero [64], the PET method works best. PEESE is also regressed in a similar way as PET. The difference is that, instead of SE (in our case ($\sqrt{1/\widetilde{ {n}_{i}}})$), the squared SE—that is, the variance (in our case ($1/\widetilde{ {n}_{i}}$) [41])—is used as the predictor [64]. If no evidence for publication bias is found, a ML-REML model can be applied [64].

We employed state-of-the-art model selection, namely randomized LASSO (least absolute shrinkage and selection operator) to examine the impact of effect modifiers on the overall effect. LASSO is a statistical technique used for variable selection and regularization in regression analysis. It is specifically designed to handle high-dimensional data where the number of predictors is larger than the number of observations. By applying a penalty to the regression coefficients, LASSO encourages some coefficients to be shrunk to zero, effectively identifying the most significant predictors [70]. This method plays a crucial role in preventing overfitting and enhancing the interpretability of the regression model. The randomized LASSO model solves the variable selection problems by resampling the data and computing a LASSO on each resampling [70, 71]. We quantified variable importance by running the LASSO model for five different model sizes (1–5 regressors) per SPP (except for NUE due to a limited number of unique papers) and generating selection probabilities for each predictor (effect modifiers). For the variables selected, we calculated the coefficient sign through ordinary least squares; the results are visualized in Additional file 12. When the selected variable had a higher probability, the model variable was found to have a stronger explanation for the biochar effect on SPPs, while the weaker effect was seen when the probability sign had a lower percentage. Consistently high probabilities indicate that the underlying variable is a robust predictor of the effect size for the respective SPP. The variables with zero-percent probability were read as unimportant predictors. We addressed publication bias in LASSO models by incorporating the square root of the effective sample size in the LASSO model. If the measure of precision is not a robust predictor, there is additional evidence that publication bias might not be present, and vice versa if the precision is robust. We implemented LASSO in *glmnet* package in *R*, and it is only based on fixed effects models [72].

We used funnel plots with 1/SE (precision) for lnRR as measures of uncertainty to visualize outliers in the dataset (Additional file 11), as suggested by Nakagawa, Lagisz [41]. Outliers that seemed to contribute to excessive noise were further investigated. Subsequently, we used a conventional boxplot method to remove these outliers from the dataset. The removal process was guided by the calculation of the first quartile, the third quartile, and the interquartile range (IQR) of 1/SE. Data points falling below the first quartile or above the third quartile by a factor of 10 times the IQR were considered outliers and removed from the analysis. A sensitivity analysis was performed to assess the presence and absence of outliers on the direction and magnitude of the effect sizes (Additional file 11).

## Review findings

### Review descriptive statistics

#### Literature searches and screening

Searches were carried out in bibliographic databases in April 2021, which returned 12,191 records.

The majority of records from bibliographic databases resulted from the EBSCO Open Dissertations, Web of Science core collection, Scopus, and AGRICOLA, with 4407, 2410, 2458, and 1612 results, respectively (Fig. 1). Other sources, such as organizational websites suggested by the advisory committee and Google Scholar, were searched in May 2021 and provided 300 and 2600 recordings, respectively (Additional file 2). We used Endnote X9 to check for duplicates, which resulted in 3371 recordings. Afterward, we conducted a manual duplicate check and found 2036 extra duplicates. After all duplicates were removed, 9684 unique records remained. Once the title and abstract screening had been implemented, 2738 studies remained. Because the amount for the full-text screening was very large, we decided to focus on one ecosystem service, namely the nutrient cycle, instead of four by applying text-mining, as we described in the section ‘screening process.’ After the text-mining, we were able to reduce the number to 1724 articles, of which the full text was screened. Full-text screening reduced the number of articles by 1142 for the reasons listed in Fig. 1. Subsequently, 582 remaining articles were critically appraised, of which 109 articles were eligible for qualitative and quantitative synthesis (see Additional file 6). Of these, 17 were not suitable for the quantitative synthesis, which meant that 92 studies were used for the data extraction and meta-analysis.

#### Study validity assessment

Most of the studies were assigned low validity due to a high risk of bias (303 articles; 52.1%). The second-largest group of studies was given moderate validity due to a moderate risk of bias (170 articles; 29.2%). The remaining studies were of high validity with a low risk of bias (109 articles; 18.7%).

**Fig. 2:**
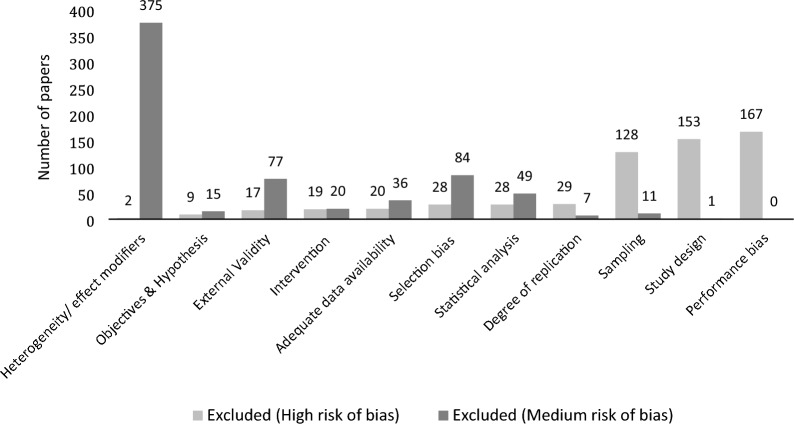
Excluded studies with high and moderate risk of bias (in percentage)

Of the studies that had a high risk of bias, most suffered from performance bias (27.8%) with confounding factors being present, or it was hard to judge whether confounders exist (Fig. 2). Because the study design was not based on proper experiments (that is, control vs. treatment), a further 25% of the studies were at high risk of bias. Another 21% of high risk of bias were due to the fact that the sampling method of the studies was not appropriate to collect data on the population in question. The remaining studies lacked clear statistical analyses (5%), mismatch of intervention and comparator (5%), inadequate data availability (3%), and intervention description (3%).

One hundred and seventy articles had a moderate risk of bias, mainly due to a lack of consideration of heterogeneity/effect modifiers (55.6%), the discrepancy between intervention and comparator (selection bias) (12.4%), and a lack of external validity (for example, the study population, in particular, cannot be generalized to sandy soils) (11.4%). Insufficient data availability (5.3%) and minimal description of statistical analysis (7.3%) were also reasons for a moderate risk of bias (Fig. 2). As mentioned earlier, only studies with a low risk of bias and high validity were used for both qualitative and quantitative analysis, thereby deviating from the protocol.

#### Publication year

**Fig. 3:**
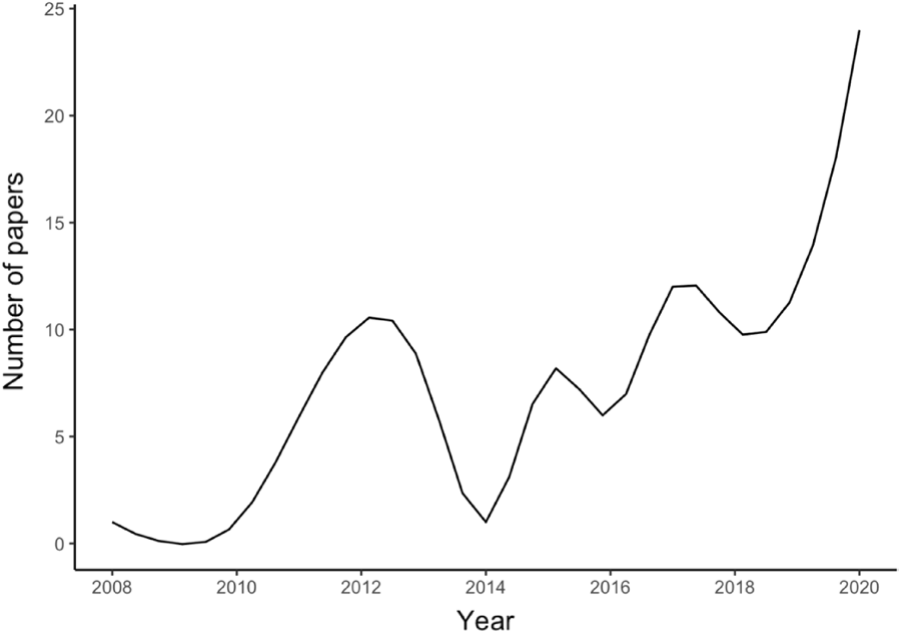
Number of publications by years

Included articles were published from 2008 to 2021. Over time, the number of publications increased—that were more than 10 times as many publications in recent years as at the beginning of this decade (Fig. 3).

### Narrative synthesis

The narrative synthesis was based on 92 articles with a low risk of bias. These included articles yielded 1609 data observations. A database of these studies with qualitative and quantitative data is available in Additional file 9. In the narrative synthesis, we cover the following aspects: study location, study design and population of interest, intervention and comparator, outcome, potential effect modifiers, and sources of heterogeneity.

#### Study location

**Fig. 4:**
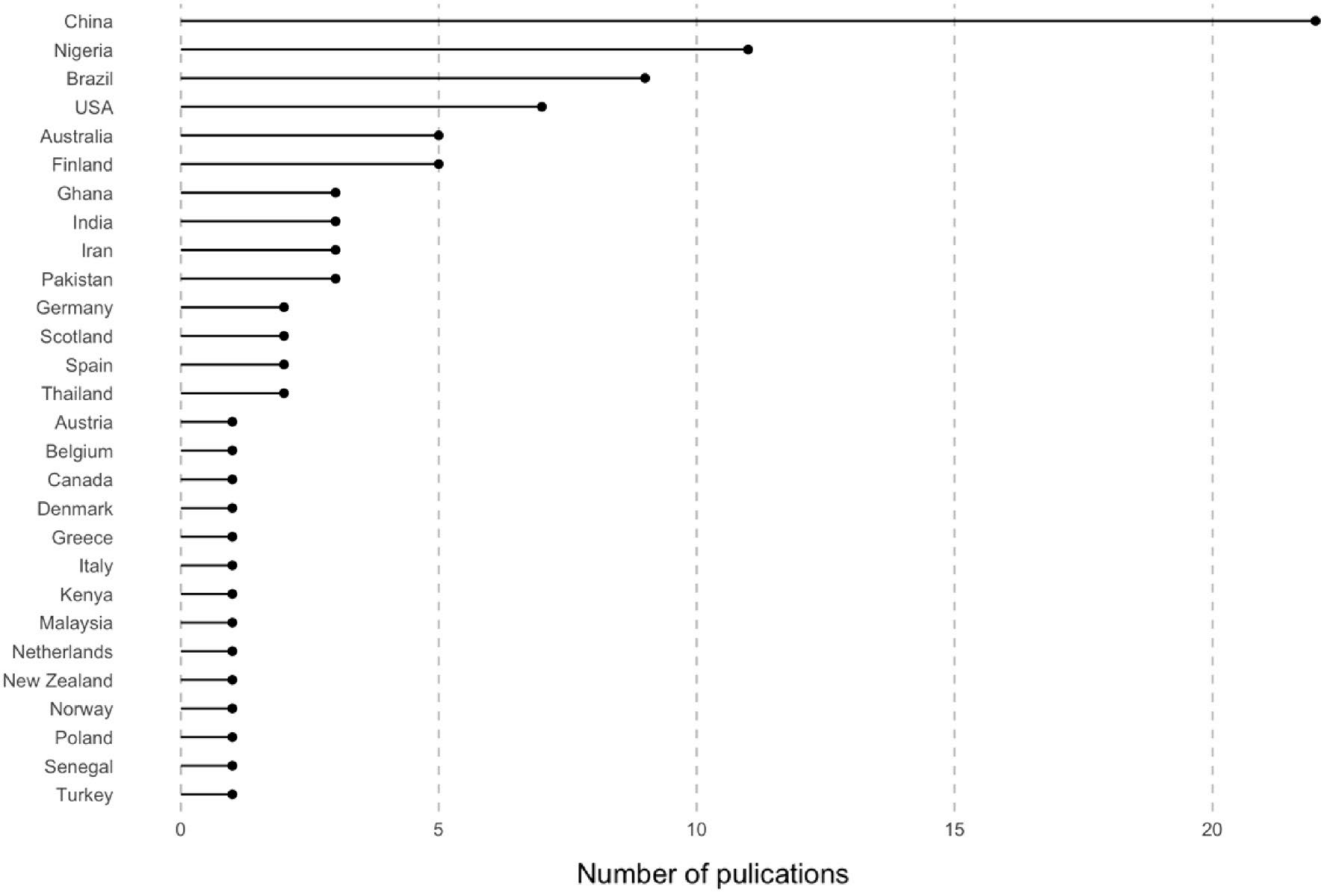
Number of publications by experimental country

Included studies were conducted across 28 countries. Asia is the continent where the highest number of studies were conducted (37.6%; Additional file 10: Table 10.1), most of which were in China (22 studies; 23.6%). A small number of contributions came from other Asian countries (such as Iran and India, which had three publications each). The second-most publications came from the European continent (20.4%), where many countries made small contributions, although Finland stands out with five studies (Fig. 4). Africa accounted for 17.2% of studies, 11 of which were conducted in Nigeria. Furthermore, 9.7% of studies were carried out in South America, all of which came from Brazil (nine studies). Finally, 8.6% of studies were conducted in North America (mostly the USA) and 6.5% in Oceania.

#### Study design and population of interest

**Fig. 5:**
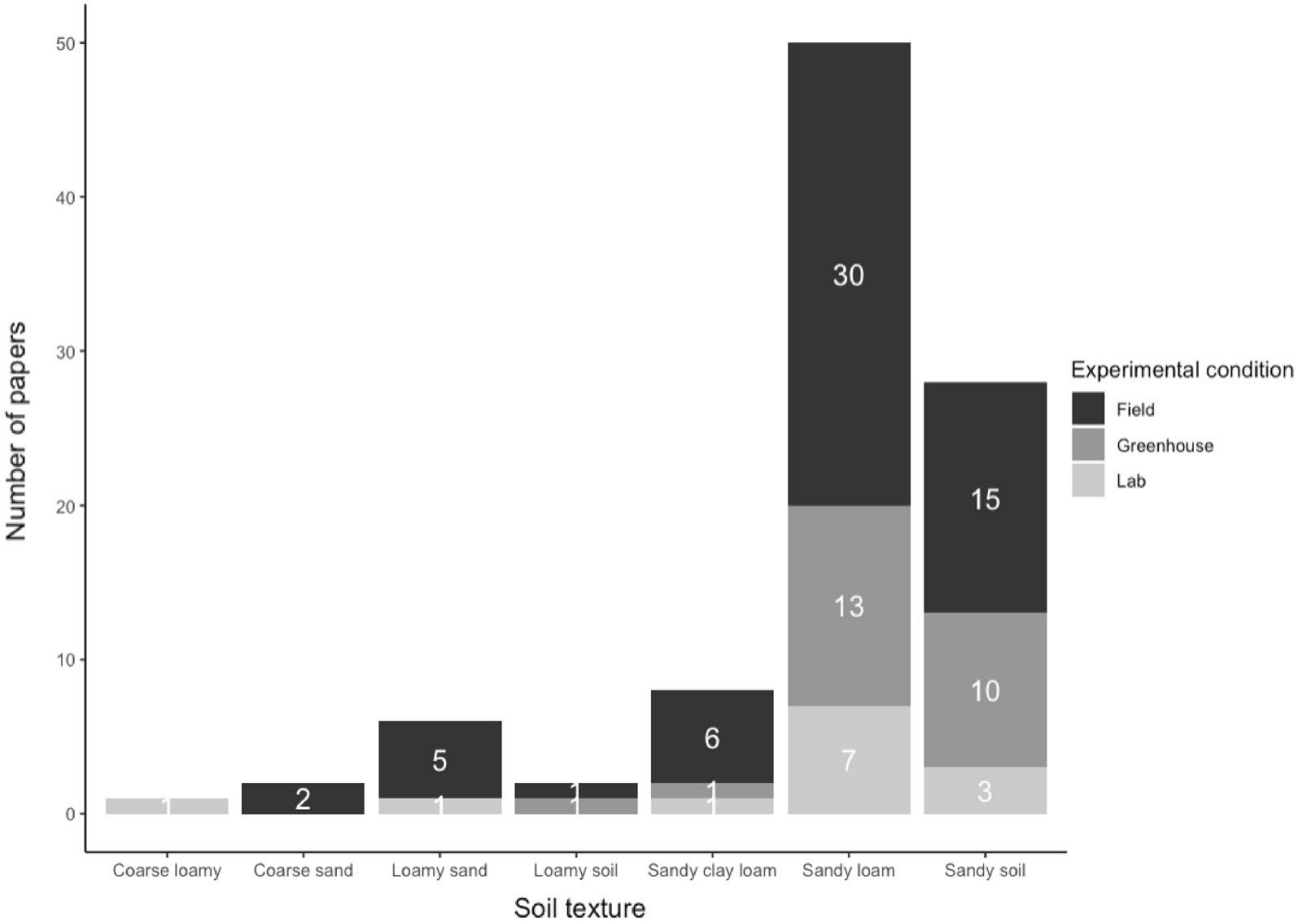
Proportion of population of interest in different study designs

Of the 92 articles retained after critical appraisal, 56 were based on field experiments (60.9%), making it the most frequent study design, often performed in sandy loam (32%) and sandy soil (16%). The remaining field experiments were conducted in sandy clay loam (6.5%), loamy sand (5.4%) and, to a lesser extent, in coarse sand and loamy soil. Greenhouse experiments were the second-most applied study design with 25 articles (27.2%), mainly used for sandy loam (13.9%) followed by sandy soil (10.8%). The remaining 11 articles (11.9%) were lab-based studies, for which sandy loam (7.5%) and sandy soil (3.2%) were the most studied. Given that the largest number of studies were carried out in sandy loam and sandy soils, their proportion in each experimental design is much higher than for other types of soils (Fig. 5).

#### Intervention and comparator

**Fig. 6:**
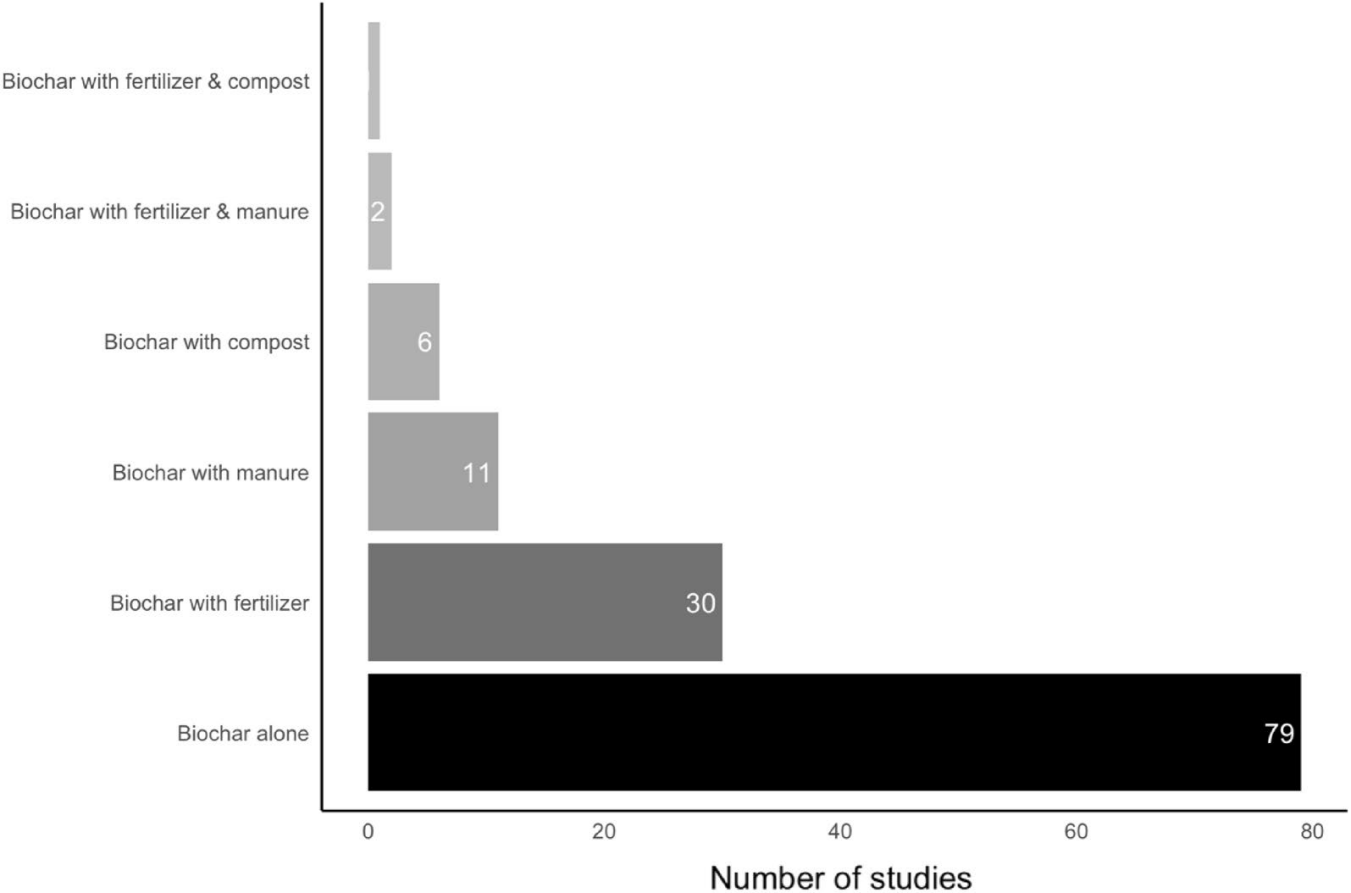
Type of treatments with their corresponding controls

There were six types of controls, with corresponding treatments. Ninety-two unique articles yielded a total of 129 counts for types of controls and treatments. Controls with no amendment and treatment alone were the most applied comparator and treatment (61.2%). A control with no amendment means that the control site has not been treated with any other treatments and biochar only means that only biochar is applied to untreated soils (Fig. 6). The second-most common type of comparator and treatment was control with fertilizer (23.2%), treated with fertilizer only and no biochar, and biochar with fertilizer, followed by control with manure without biochar and biochar with manure (8.5%). There were also experimental studies (4.7%) where the control was treated with compost and biochar with compost being its intervention. The remaining studies (2.4%) applied control with fertilizer and manure, and control with fertilizer and compost, and their associated treatments. The effect of these treatments varies across different SPPs.

#### Outcome

**Fig. 7:**
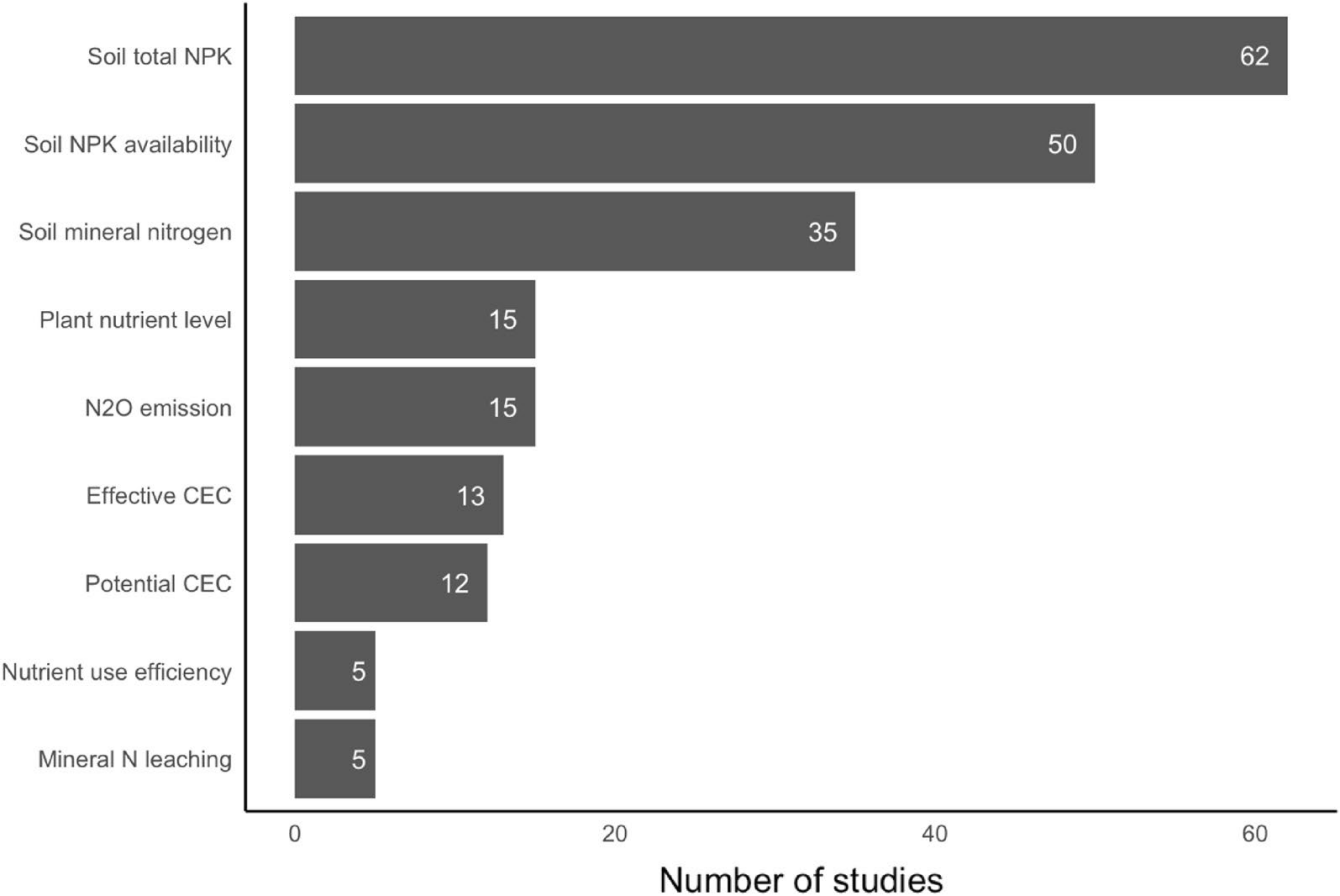
Number of articles for each SPP

In total, there were 49 soil properties that were grouped by their relevance into nine SPPs: mineral N leaching, effective CEC, potential CEC, N_2_O emission, NUE, plant nutrient level, soil mineral nitrogen, soil NPK availability, and soil total NPK. Soil total NPK was studied most (28.9%), followed by soil NPK availability (23.7%) and soil mineral nitrogen (16.6%; Fig. 7). There were as many articles on plant nutrient level as on N_2_O emissions (7.1%), while there were 13 articles on effective CEC and 12 on potential CEC. Less studied SPPs were NUE and mineral N leaching (both representing 2.4% of articles).

#### Effect modifiers and sources of heterogeneity

According to the data, some effect modifiers were reported in almost every paper, such as geographic location, experimental design, types of feedstock, and pyrolysis temperature. Annual temperature and biochar application rate were overlooked a few times. The least reported effect modifiers were the type of climate and annual precipitation. Soil pH, biochar pH, soil bulk density and soil dry weight were barely reported. Hence, to provide consistency in the analysis, if there were more than 50% missing values for any of the effect modifiers, they were no longer considered effect modifiers in the analysis (Additional file 9).

### Quantitative synthesis

#### Description of the data from primary studies included in the meta-analysis

Most observations (1341) were retrieved from 83 peer-reviewed while 268 observations from nine articles were grey literature, comprised of five dissertations (120 observations), three master’s theses (132 observations), and one university repository (16 observations). A significant number of observations came from Asia (467 observations), followed by Africa (412). The remaining observations came from Europe (259), South America (231), and Oceania (153). The fewest observations were supplied from North America [86].

Various types of feedstocks were used for the production of biochar, which were classified into three categories: agricultural residue, manure/digestate, and woody biomass [74]. Among these, agricultural residues dominated both in the number of articles [52] and in the number of data sets (844, after aggregation). Woody biomass was also often studied, with 585 observations aggregated from 42 articles (Table 6). On the contrary, only 14 articles considered manure/digestate, with 180 observations. Articles were also categorized with regard to pyrolysis temperature: ‘low’ when the pyrolysis temperature is less than or equal to 400 ℃, ‘medium’ when the temperature is between 400 and 600 ℃, and ‘high’ when the temperature is equal to or greater than 600 ℃ [38]. In total, 52 articles were conducted on medium pyrolysis temperature, with 823 observations; low pyrolysis temperature was studied in 30 articles, yielding 549 aggregated data sets, and high pyrolysis temperature was not applied in many experimental studies, with 173 data sets from 14 articles. There were four articles with 64 missing data sets (Table 6). Altogether, this represents more than 92 unique articles, because several unique articles have not studied just one type of pyrolysis temperature or type of feedstock, but several.

**Table 6:** Description of the observations included in the meta-analysis

Variables	Categories	Subcategories	Unique papers (number of observations)
Paper type	Peer-reviewed	Peer-reviewed	83 (1,341)
	Gray literature	Dissertations	5 (120)
		Master’s theses	3 (132)
		University repository	1 (16)
Experimental design	Field experiment	Field	53 (893)
	Controlled experiments	Greenhouse	19 (505)
		Laboratory	11 (211)
Experimental continents	Africa	–	16 (412)
	Asia	–	35 (468)
	Europe	–	19 (254)
	North America	–	8 (85)
	Oceania	–	6 (149)
	South America	–	9 (231)
Feedstock type	Agricultural residue	–	52 (844)
	Manure/digestate	–	14 (180)
	Wood-based	–	42 (585)
Pyrolysis group (°C)	≤ 400	Low	30 (549)
	400 < ; > 600	Medium	52 (823)
	≥ 600	High	14 (173)
Biochar application rate (t/ha)	< 10	–	51 (664)
	10–30	–	49 (536)
	30–50	–	17 (194)
	50 <	–	19 (215)
Experiment duration (days)	< 60	–	14 (246)
	60–160	–	28 (503)
	160–402	–	23 (384)
	402–730	–	20 (318)
	730 <	–	9 (152)
Types of treatments	Biochar alone	–	79 (1,020)
	Biochar with compost	–	6 (33)
	Biochar with manure	–	11 (127)
	Biochar with fertilizer	–	30 (401)
	Biochar with fertilizer & compost	–	1 (6)
	Biochar with fertilizer & manure	–	2 (12)
Tillage application	Not tilled	–	64 (1,047)
	Tilled	–	29 (552)
Irrigation application	Not irrigated	–	51 (1,053)
	Irrigated	–	42 (556)
Base fertilizer application before and after biochar	Fertilizer applied	–	42 (589)
	Fertilizer not applied	–	51 (1,010)
SPPs	Soil total NPK	–	61 (506)
	Soil mineral nitrogen	–	35 (247)
	Plant nutrient level	–	15 (106)
	N_2_O emission	–	15 (53)
	Soil NPK availability	–	50 (468)
	Potential CEC	–	12 (37)
	Effective CEC	–	13 (49)
	NUE	–	5 (23)
	Mineral N leaching	–	5 (44)

For analysis purposes, the biochar application rate is also divided into four groups based on data availability, such as ‘ < 10’, ‘10–30’, ‘30–50’ and ‘ > 50’ t/ha. Biochar application rates ‘ < 10’ and ‘10–30’ were the most commonly applied rates, the former being applied in 51 articles with 664 observations and the latter in 49 articles with 536 aggregated observations. The remaining two other categories, ‘30–50’ and ‘ > 50’, generated 194 observations from 17 articles and 215 observations from 19 articles, respectively (Table 6). Given that the duration of the experiment varied in each study, we classified them into five groups as follows: ‘ < 60,’ ‘60–160,’ ‘160–402,’ ‘402–730,’ and ‘ > 730’ days. The most common experiment duration was ‘60–160’ days, studied in 28 articles (503 data sets). Subsequent experiments of ‘160–402’ and ‘402–730’ days were with data sets of 388 and 319, respectively (Table 6). Observations 246 and 152 were assigned to the shortest and longest experimental periods of ‘ < 60’ and ‘ > 730’days, respectively.

There were 42 articles with 556 observations that applied irrigation before and after biochar application, while 51 articles reported no irrigation application. Some studies also mentioned the use of tillage, which was only applied in field experiments, and 29 articles reported the use of tillage during the experiment with 552 observations (Table 6). Several control soils were treated with inorganic or organic fertilizer before being used in the experiment. Specifically, 42 articles (589 observations) indicated fertilizer as a base treatment. These effect modifiers, in turn, can affect the overall effect of biochar on SPPs, which we discuss in the following sections.

#### The effect of biochar on SPPs related to nutrient cycle

**Fig. 8:**
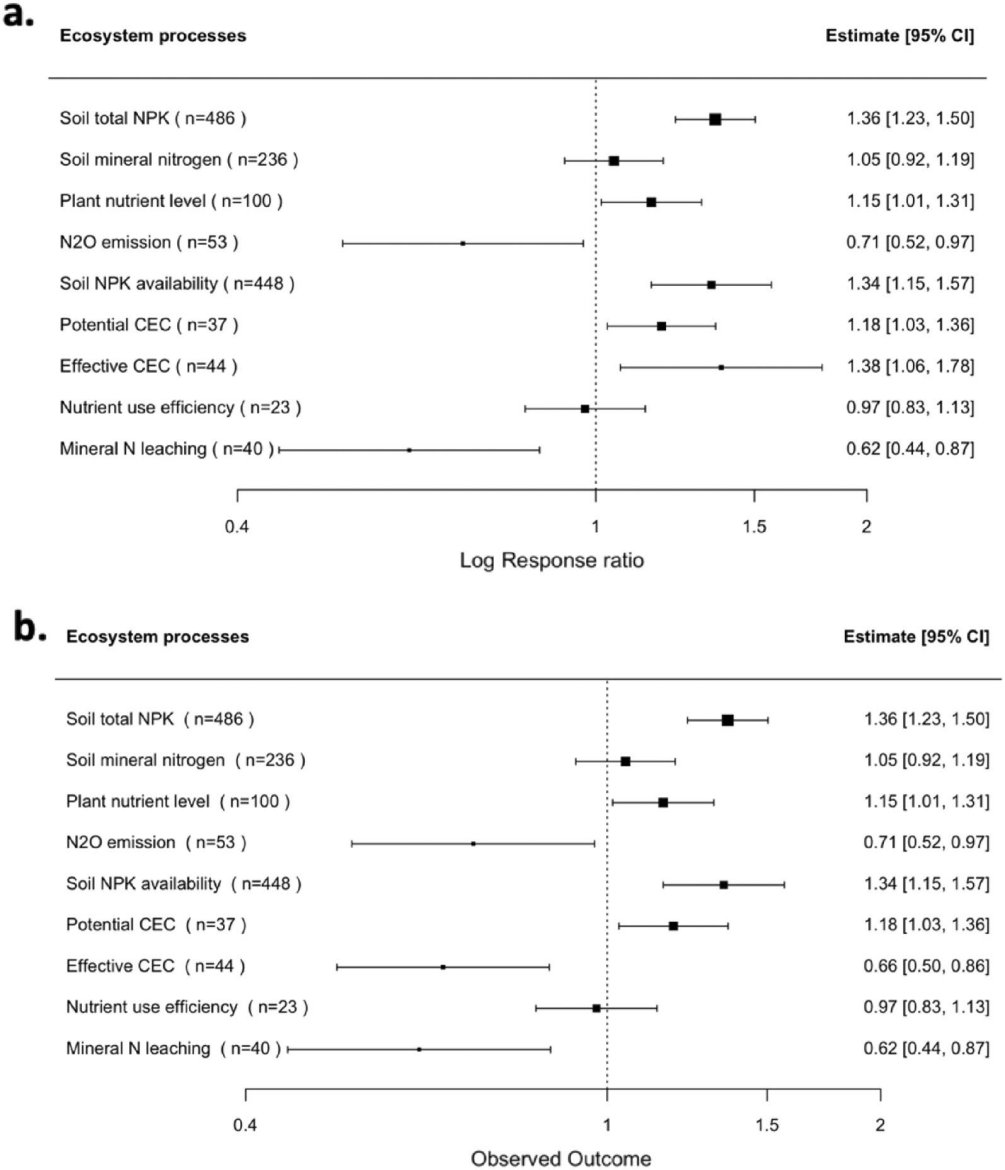
The response of SPPs to biochar application, n means the number of observations: **a** estimated meta-averages without correction for publication bias; **b** estimated meta-averages corrected for publication bias (PET & PEESE model). An effect is significant (P < 0.05) if its 95% confidence interval (CI) does not include 1. The biochar treatment is considered to have no effect when the $lnRR=1$, a positive effect when $lnRR>1$, and a negative effect when $lnRR <1$. Confidence intervals are not symmetrical around the effect sizes because they were back-transformed from the log response ratio

The results of nine meta-analyses are shown in Fig. 8 (one meta-analysis for each of the nine SPPs). Of the total observations used in the meta-analysis, 4% were detected as outliers based on the criterion set in the method section of “Effect Size and its Variance” (Additional file 11). When we removed outliers to analyze the sensitivity of the results, the mean effect sizes did not vary from positive to negative, from significant to non-significant, or vice versa, for any of the SPPs reported here. Therefore, we consider the results to be robust to the outlier removal and we only provide the results without outliers.

Figure 8 presents the estimated meta-averages for the nine SPP without correction for publication bias (Fig. 8a) and with correction for publication bias (Fig. 8b). Visual inspection of funnel plots and the PET-PEESE models suggest that publication bias is of minor importance in the literature (Additional file 13). This is also consistent with the observation that the estimated meta-average and confidence intervals are similar for the models with and without correction for publication bias (Fig. 8). An exception applies for Effective CEC, where the estimate changes from 38% (CI [6%, 78%]) to − 34% (CI [− 50%, − 14%]).

Other SPPs of sandy-textured soils had an effect after biochar amendment before and after the publication bias correction. When the CI of the effect size did not overlap with zero, it was deemed significantly different from zero. For example, the soil total NPK of sandy-textured soils increased by 36% (CI [23%, 50%]) after being treated with biochar. Soil mineral nitrogen was also augmented after biochar application by 5%; however, it was not significantly different from a null (CI [− 8%, 19%]). This is followed by plant nutrient levels, which had an increase of 15% (CI [1%, 31%]) in sandy-textured soils induced with biochar. N_2_O emissions were reduced significantly by 29% (CI [− 48%, − 3%]) after biochar, resulting in decreased emissions into the atmosphere. Soil NPK availability was the second highest responded SPP of sandy-textured soils following biochar application (34%, CI [15%, 57%]), followed by potential CEC (18%, CI [3%, 36%]). Since no publication bias was detected in the response of these SPPs, the responses with correction for publication bias support the findings without correction for publication bias (Figs. 8a and b).

No significant difference was found between control and biochar treatments for NUE (− 3%, CI [− 17%, 14%]). Finally, mineral N leaching from sandy-textured soils was reduced by 38% (CI [− 56%, − 13%]), contributing to less groundwater contamination. The results were practically the same before and after correction for publication bias. It should be noted that meta-analyses of some SPPs—such as NUE and mineral N leaching—are based only on a few primary studies, so the corresponding results should be read with caution.

**Fig. 9:**
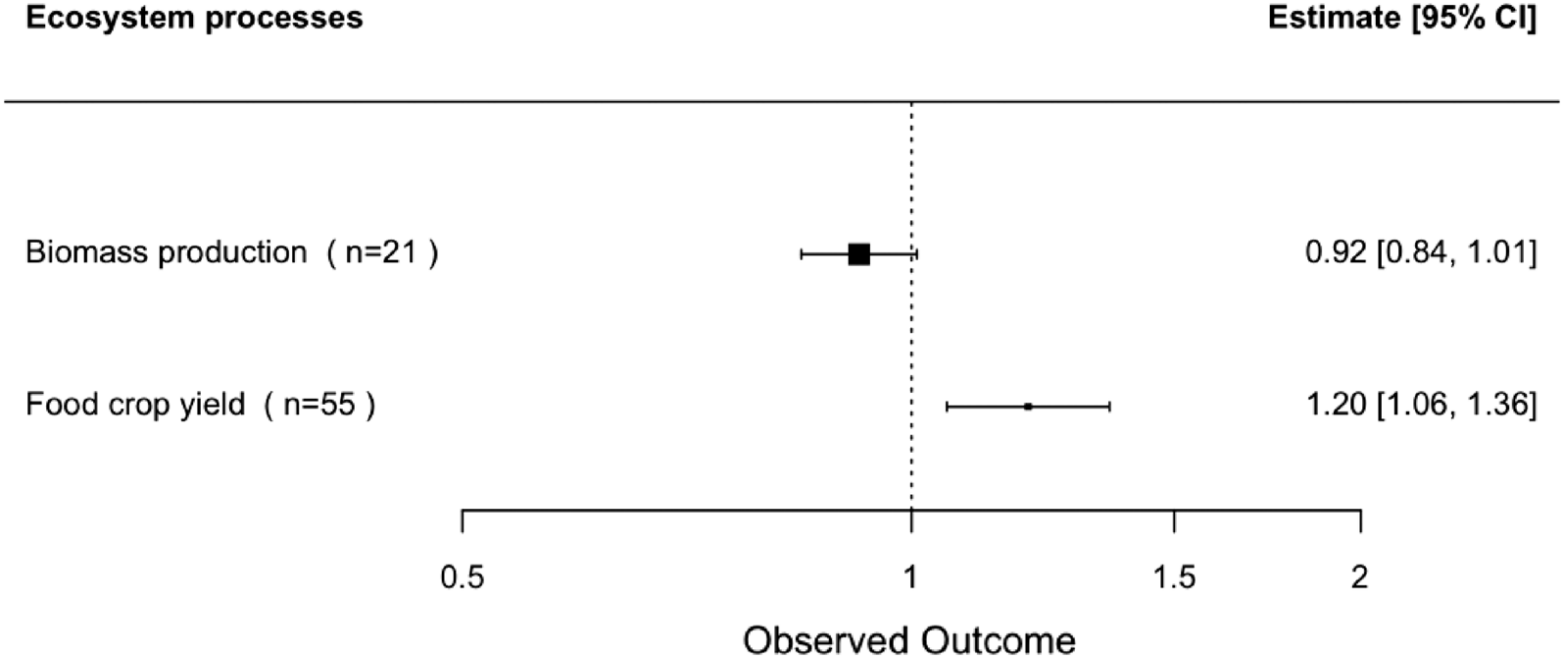
The response of food crop yield and biomass production to biochar application, n means the number of observations. An effect is significant (P < 0.05) if its 95% confidence interval (CI) does not include 1. The biochar treatment is considered to have no effect when the $lnRR=1$*,* a positive effect when $lnRR>1$, and a negative effect when $lnRR <1$. The reason why confidence intervals are not symmetrical around the effect sizes is that they were transformed back from the log response ratio

In addition to SPPs, we also performed a supplementary analysis to assess the effect of biochar on food crop yield and biomass production based on the observations from the same papers. Food crop yields are annual food crops such as cereals, maize, wheat, and rice, as well as fruits and vegetables, while biomass production is defined here as the shoot of a plant and all types of plants that are produced as above-ground biomass and do not include the harvested portion of food crops. As shown in Fig. 9, food crop yield increased by 20% (CI [6%, 36%]) in sandy-textured soils treated with biochar, but biomass production showed no effect (− 8%, CI [− 16%, 1%]). No publication bias effect was detected.

Provided that 60% of the included studies did not report SD or SE to calculate the variations of the total effect sizes, they were imputed with the pooled CV using data from studies that did report SDs as described in the methods section of ‘*Effect size and its variation calculation.’* A sensitivity analysis was conducted by removing all observations with variations determined based on pooled CV, which changed some of the average treatment effects to non-significant (Additional file 11: Fig. S11.11). This is because fewer observations result in less power, and the loss of significance is not surprising. If the variations had not been determined (based on the pooled CV) when SD or SE was absent, huge amounts of important data would have been missed in the analysis, and we would have been limited to drawing a conclusion based on a few datasets.

#### Explaining heterogeneity: meta-regression analysis of each SPP

We analyzed the reasons for variations in the response of each SPP to biochar application using the effect modifiers mentioned in the sub Sect. "Potential Effect Modifiers and Reasons for Heterogeneity". We present the results of the LASSO analysis if the variables selected in a minimum of one out of five LASSO models as explained in the sub-section of Effect Size and its Variance. The visualization of the full variable selection is given in Additional file 12. Based on our analysis, the following moderators were the main predictors explaining the heterogeneity in the responses of SPPs to biochar application: experimental continent, duration, biochar application rate, fertilizer application rate, soil pH, and publication year. In the sections below, we discuss the key predictors for the response of each SPP based on Table 7, and the results are visualized.

**Table 7:** Coefficient signs following Randomized LASSO selection for each SPPs

	Soil total NPK	Soil mineral nitrogen	Plant nutrient level	N_2_O emission	NPK availability	Potential CEC	Effective CEC	NUE	Mineral N leaching
Study location and Climate									
Continent	±		±		+	+	+		
Annual temperature									
Annual precipitation									
Experimental condition									
Experimental design					–				
Experimental duration		–		±			+		
Biochar application rate	+	+	+	-	±	+			–
Biochar with fertilizer									
Fertilizer application rate	–					–			±
Biochar with manure									
Manure application rate									
Biochar properties									
Feedstock types									
Pyrolysis temperature			+						–
Soil status									
Soil texture		–			+				
Soil pH		+		–		–	–		
Soil minimum depth									
Soil maximum depth							+		
Fertilizer before biochar									
Manure before biochar									
Irrigation application									
Tillage application									
Publication status and bias									
Publication year	+					–	+		-
Square root of effective sample size		+	+	+	+	+	+		

The direction of effect: “−” means that the effect of the predictor was negative in all selected models; “ + ” means the predictor was positive in all selected models. The effect direction was consistent in the LASSO analysis; that is, the effect of the predictor was always positive or always negative. To determine the coefficient sign for the selected variables, we employed ordinary least squares (79) regression. However, we could not perform LASSO analyses due to a scarcity of available papers on NUE and mineral N leaching

##### Soil total NPK

**Fig. 10:**
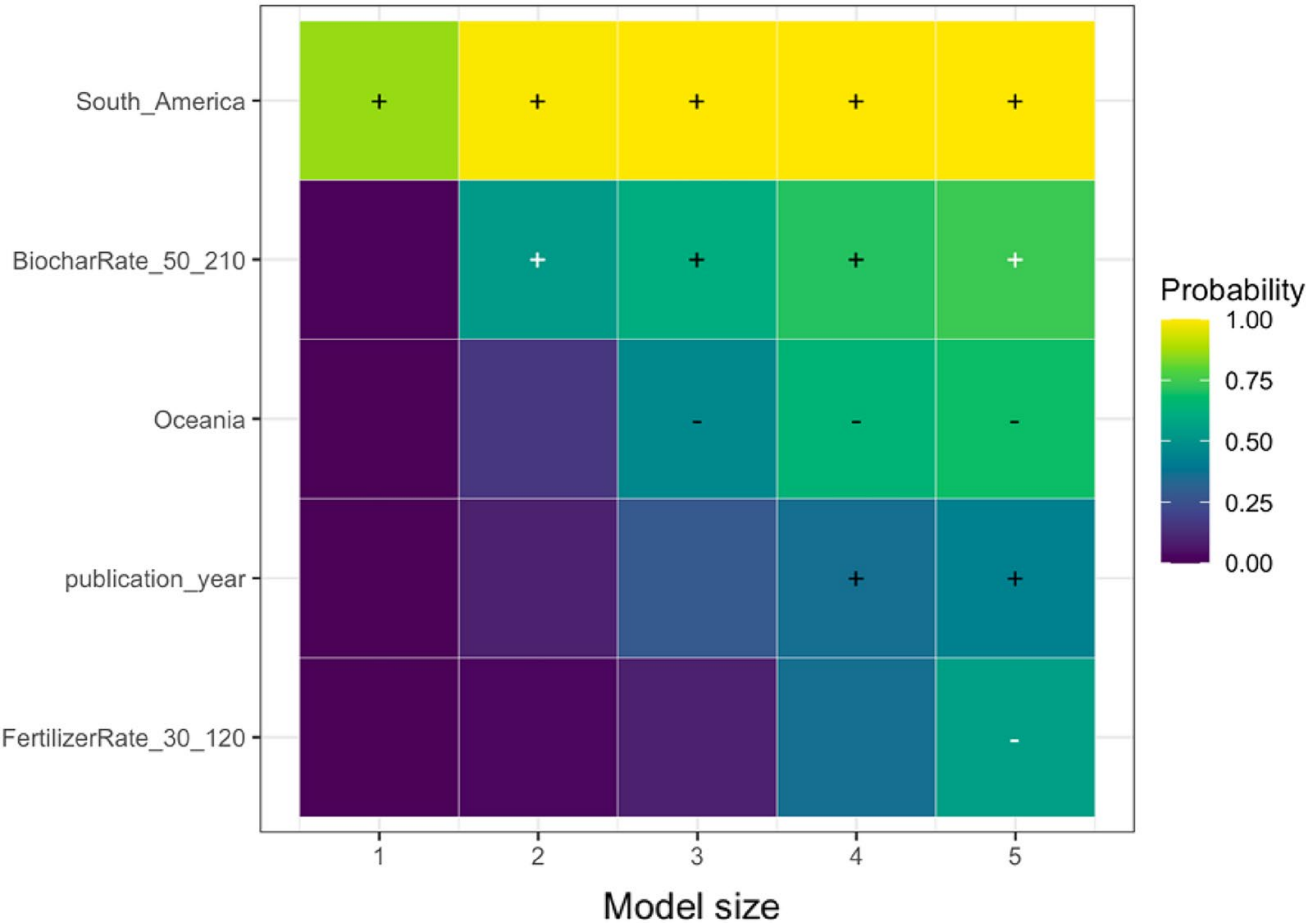
The results of randomized LASSO on the key predictors in soil total NPK response. The importance of a variable is quantified by running the LASSO model for five different model sizes (1–5 regressors). This visualization shows the variables selected in a minimum of one out of five LASSO models

According to the LASSO analysis, the most important predictors affecting the response of total soil NPK were biochar experimental continent, biochar application rate, fertilizer application rate, and publication year (Table 7). Specifically, the experimental continent, such as South America, stood out as the most important and key predictor, positively influencing the response. In contrast, the response of soil total NPK was negative in Oceania. This variation can be attributed to the region’s specific climate and environmental factors, notably rainfall patterns and temperature fluctuations. South America’s climate has appeared to enhance total NPK in sandy-textured soils following biochar application. Biochar application rate, particularly the highest application rate, was the second important predictor for the response of soil total NPK (Fig. 10), with a positive impact on the response. Several studies showed that higher biochar application rates led to an increase in total NPK content in sandy-textured soils [8], contributing to their improved quality and fertility. A higher application rate of biochar enriches soil nutrient content through mechanisms such as increased soil pH, increased organic matter, and improved CEC [75, 76]. LASSO analysis also underlined the significance of medium-level fertilizer application with biochar as a predictor, demonstrating its negative impact on the response of soil total NPK (Fig. 10). This could be attributed to the relatively lower fertilizer application rate as some studies have demonstrated significant benefit of higher fertilizer application rates in combination with biochar for soil NPK content [77, 78]. Publication year was also highlighted as an influential predictor, indicating a positive trend in soil total NPK response to biochar application over time.

##### Soil mineral nitrogen

The significant predictors of soil mineral nitrogen response to biochar application were soil pH, biochar application rate, soil texture, and experimental duration (Table 7). A positive response in soil mineral nitrogen to biochar application was observed when the soil pH ranged from 5.6 to 6.5. This finding aligns with the results reported by [80, 81]. In sandy-textured soils, higher pH levels contribute to reducing soil acidity, creating a favorable environment for processes like mineralization and nitrification, ultimately leading to increased soil mineral nitrogen. According to LASSO, a higher biochar application rate increased soil mineral nitrogen, likely due to enhanced microorganism activity promoting greater N mineralization and biochar components with a high C:N ratio stimulating N immobilization [80, 82].

**Fig. 11:**
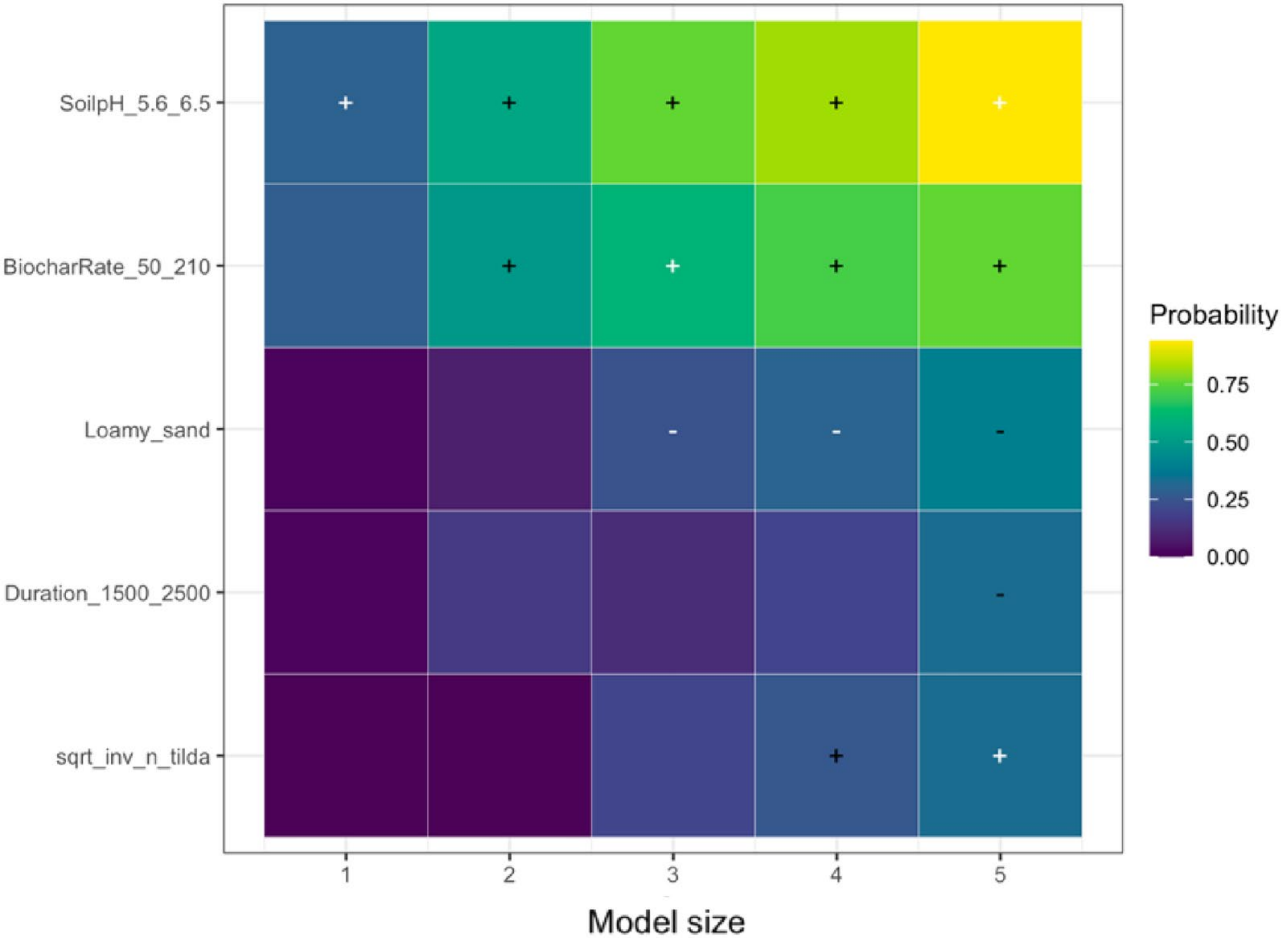
The results of randomized LASSO on the key predictors in soil mineral nitrogen response. The importance of a variable is quantified by running the LASSO model for five different model sizes (1–5 regressors). This visualization shows the variables selected in a minimum of one out of five LASSO models

Loamy sand had a negative impact on the response (Fig. 11), which could be a result of limited improvement in soil temperature, moisture, and aeration, which, in turn, failed to stimulate soil mineral nitrogen with biochar application [83–85]. Furthermore, the influence of biochar on soil mineral nitrogen appears to diminish over an extended duration (Fig. 11). As explained by Singh and Cowie [86], this diminishing effect can be attributed to the depletion of labile soil organic carbon [87]. We addressed publication bias with the square root of the effective sample size in the LASSO model, and according to the analysis, publication bias was also detected.

##### Plant nutrient level

**Fig. 12:**
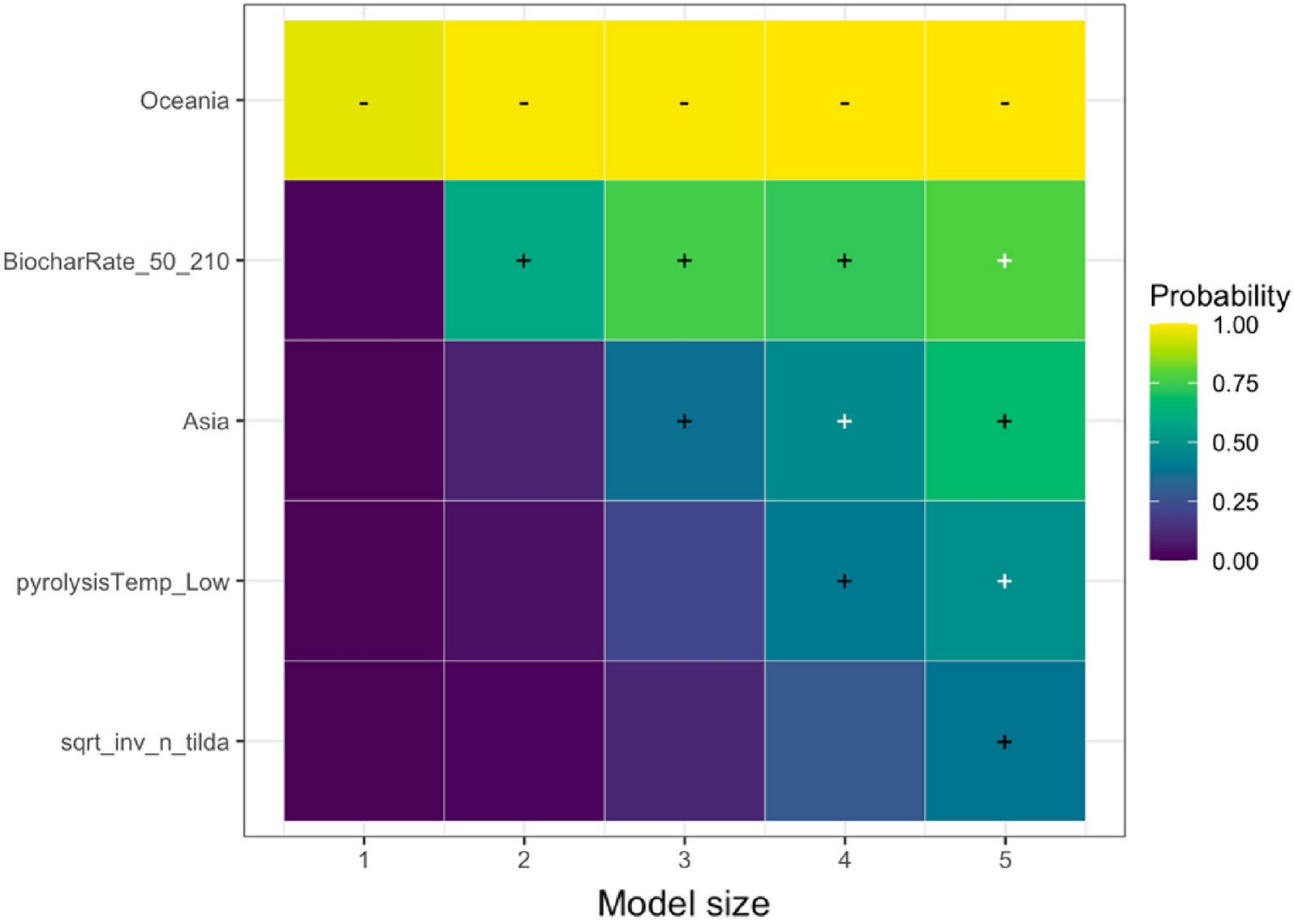
The results of randomized LASSO on the key predictors in plant nutrient level response. The importance of a variable is quantified by running the LASSO model for five different model sizes (1–5 regressors). This visualization shows the variables selected in a minimum of one out of five LASSO models

The key predictors influencing the response of plant nutrient levels were the experimental continent, biochar application rate, and pyrolysis temperature (Table 7). The negative influence of biochar on plant nutrient uptake in Oceania and its positive response in Asia can be attributed to regional environmental conditions (Fig. 12). Biochar application rates, mainly higher rates, had a positive effect on the response of plant nutrient levels (Fig. 12). In contrast, some studies found that higher application rates may significantly reduce nutrient uptake [88, 89]. However, Liu, Zhang [90] highlighted biochar’s ability to maximize N uptake in soils with poor structure, such as sandy soils. The benefit of biochar application to plant nutrient levels in sandy soils can be explained by biochar’s ability to improve soil structure and water-holding capacity [91]. LASSO analysis also identified low pyrolysis temperature as having a positive impact on the response (Fig. 12). Biochar produced at low temperatures can lead to biochar with higher nutrient content that can support nutrient release over time [92], making it beneficial for plant nutrient uptake. The results also indicate the presence of publication bias that is positively impacting on the response.

##### *N*_*2*_*O emission*

**Fig. 13:**
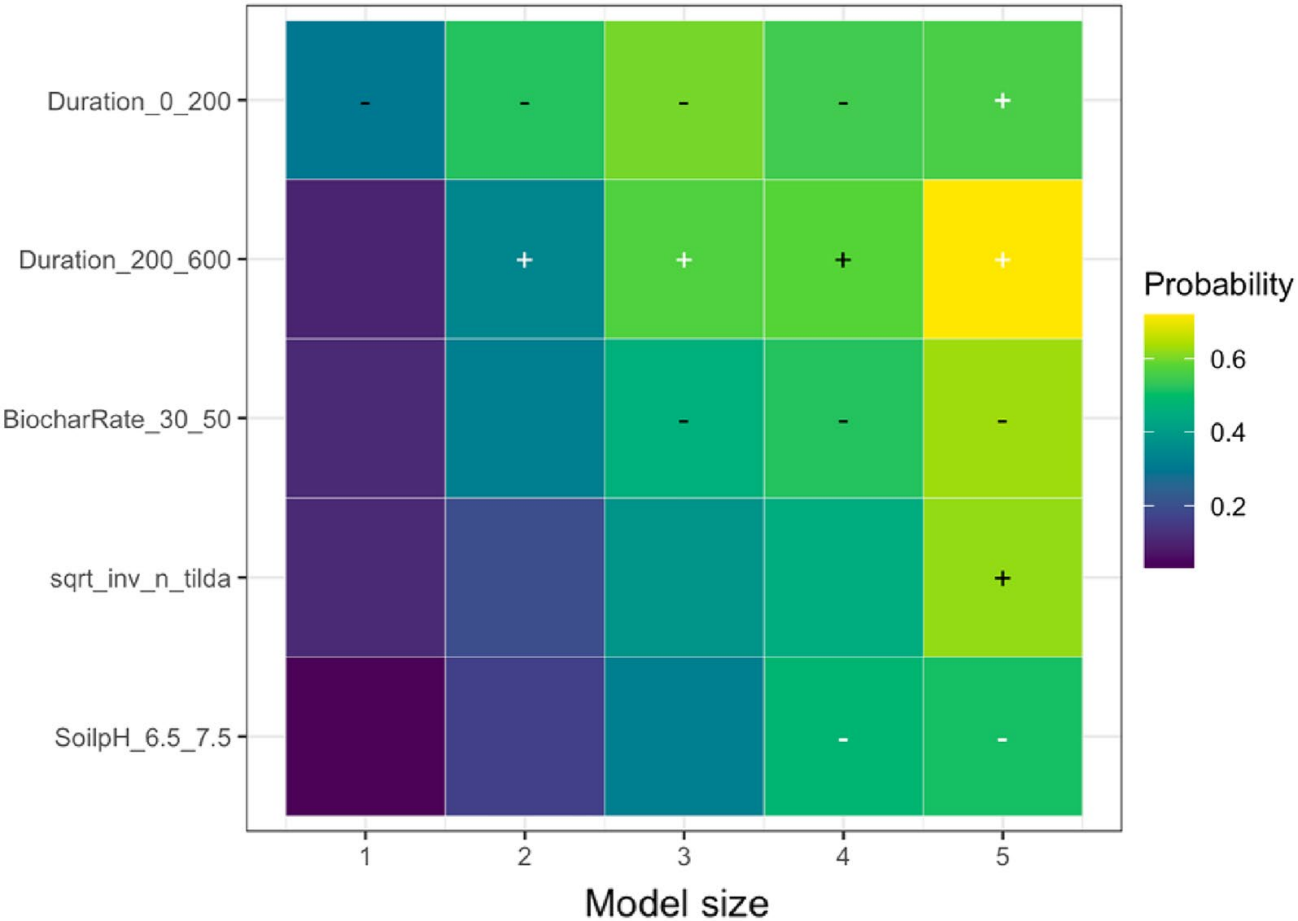
The results of randomized LASSO on the key predictors in N_2_O emission response. The importance of a variable is quantified by running the LASSO model for five different model sizes (1–5 regressors). This visualization shows the variables selected in a minimum of one out of five LASSO models

Experiment duration, biochar application, and soil pH were selected as influential predictors by LASSO (Table 7) for the N_2_O emission response. Biochar had a negative effect on the short-term response of N_2_O emissions but showed a positive effect in the long term (Fig. 13). This pattern could be related to the volatile content of biochar, which could potentially explain the short-term N_2_O emissions in sandy soils amended with biochar [93]. The application of biochar at a medium rate (30–50 t/ha) resulted in a decrease in the response of N_2_O emissions (Fig. 13). This outcome can be explained by the influence of biochar on various soil N transformation processes in sandy soils, namely enhanced mineralization and nitrification and a reduction in denitrification [94, 95]. The N_2_O emissions in soil occur mainly as a microbial process, involving nitrifiers that oxidize ${{\text{NH}}}_{4}^{+}$ under aerobic conditions and denitrifiers that reduce ${{\text{NO}}}_{3}^{-}$ under anaerobic conditions [90]. As it can be seen in Fig. 13, a decrease in N_2_O emission following biochar is observed in soils with higher pH (6.5–7.5), consistent with findings from the meta-analysis conducted by Liu, Zhang [90]. Biochar reduces N_2_O emissions in high-pH soils by promoting nitrification, converting ${{\text{NH}}}_{4}^{+}$ to ${{\text{NO}}}_{3}^{-}$, as elevated pH levels improve aeration and microbial activity [94]. The results also indicated a slightly positive effect of publication bias on response (Fig. 13).

##### Soil NPK availability

**Fig. 14:**
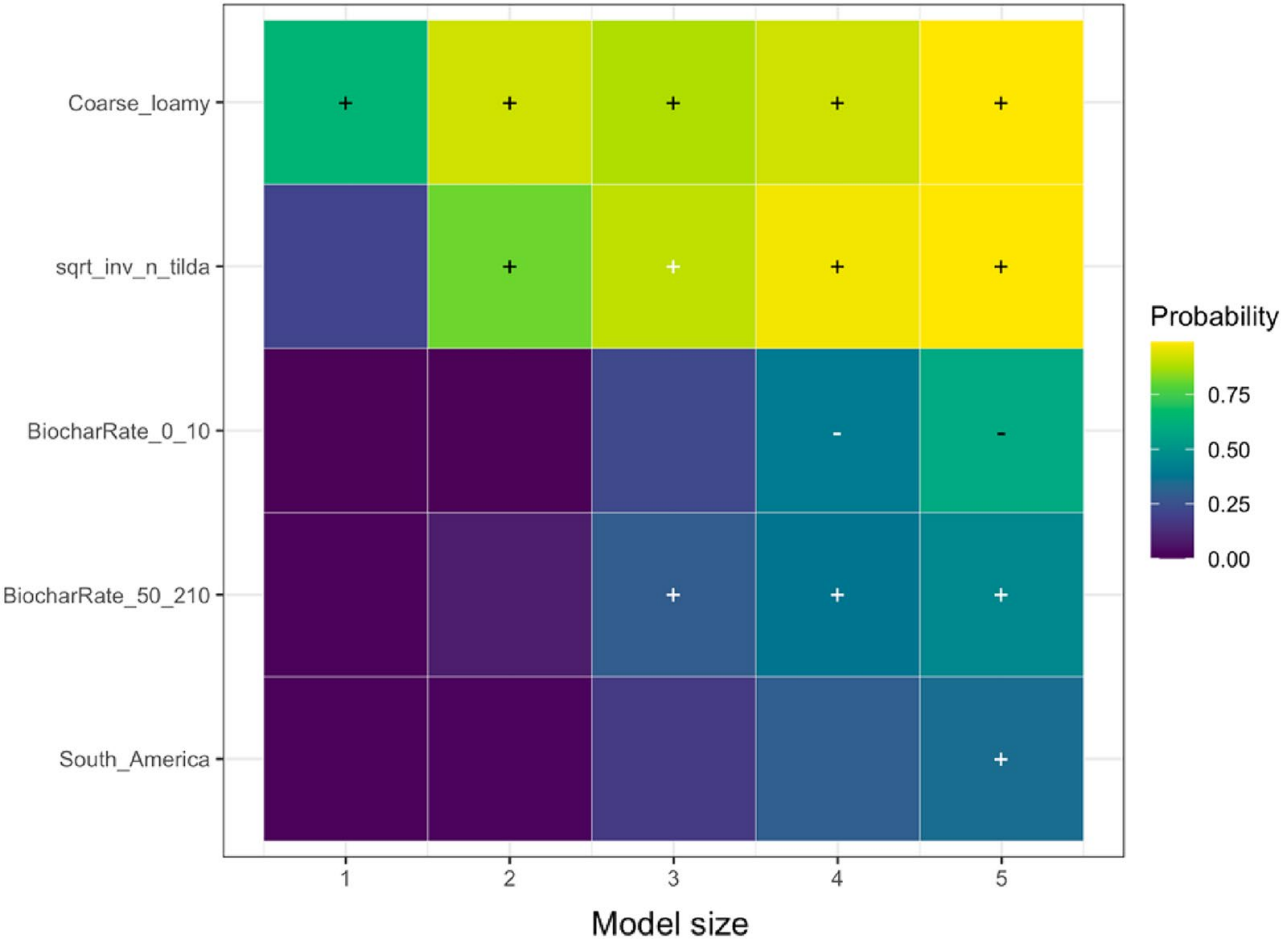
The results of randomized LASSO on the key predictors in NPK availability response. The importance of a variable is quantified by running the LASSO model for five different model sizes (1–5 regressors). This visualization shows the variables selected in a minimum of one out of five LASSO models

Soil texture, biochar application rate, and the experimental continent were the most influential and robust predictors of soil NPK availability response (Table 7). Soil texture, namely coarse loamy, was one of the most important predictors with a positive impact on the response of NPK availability following biochar (Fig. 14). The porous nature of coarse loamy soils may allow biochar to be integrated into the soil structure effectively, promoting nutrient retention and reducing nutrient leaching [89]. A low biochar application rate negatively impacted the NPK availability response, while a high application rate had a positive effect (Fig. 14). Sandy-textured soils have lower nutrient retention capacity [96], leading nutrients to leach more easily. High biochar application rates may increase the nutrient-holding capacity of sandy soils, preventing leaching and making them available to plants [89]. Furthermore, higher application rates might provide a greater surface area for nutrient adsorption, which may allow biochar to adsorb and hold more nutrients [97], making them available in the soil. LASSO analysis also identified a positive response of NPK availability to biochar application in South America (Fig. 14). Additionally, LASSO revealed the square root of the effective sample size as an influential predictor with a positive effect, signifying the impact of publication bias on the response.

##### Potential CEC

**Fig. 15:**
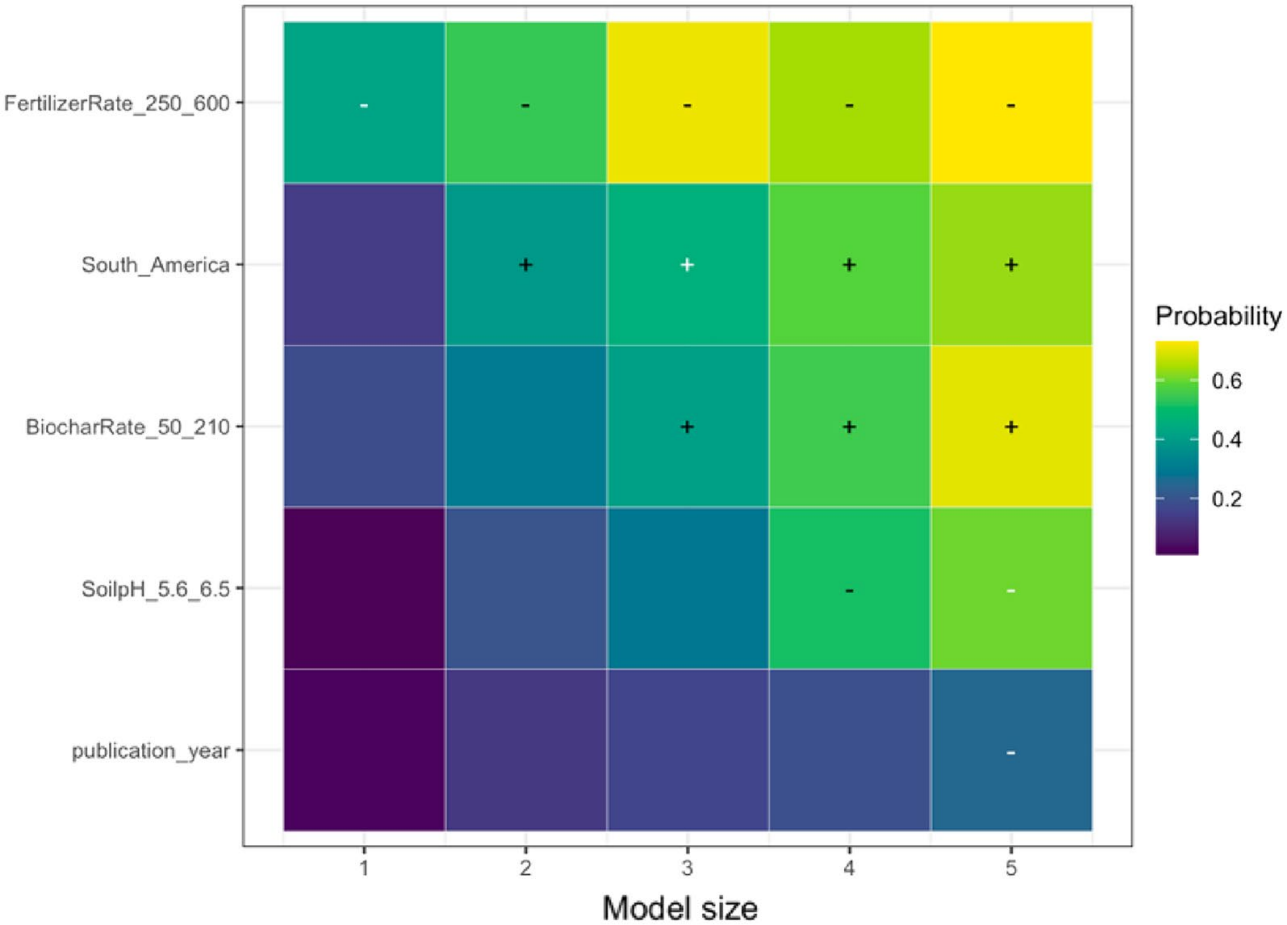
The results of randomized LASSO on the key predictors in potential CEC response. The importance of a variable is quantified by running the LASSO model for five different model sizes (1–5 regressors). This visualization shows the variables selected in a minimum of one out of five LASSO models

According to LASSO, fertilizer application rate with biochar, experimental continent, biochar application rate, and soil pH were among the most important and robust predictors for potential CEC response (Table 7). Higher rates of fertilizer application with biochar had an adverse impact on the response of potential CEC (Fig. 15). A reduction in potential CEC may result from declines in calcium (${{\text{Ca}}}_{2}^{+})$ and magnesium (${{\text{Mg}}}_{2}^{+})$ after higher fertilizer application rates [98], or disruption in the balance of base cations in the sorption composite [99]. Additionally, a higher application rate of fertilizer with biochar may cause a dilution effect that may further diminish potential CEC [100]. On the other hand, only biochar application at a higher rate was favorable for the response of potential CEC in sandy-textured soils (Fig. 15) This can be linked to different properties of the biochar, as they can vary in composition, surface area, and charge characteristics [100]. Some biochars may have a higher affinity for retaining certain cations, while others may not be as effective. The experimental continent, especially South America, was a crucial predictor and positively influenced the response. As can be seen in Fig. 15, the response of potential CEC was negative at lower soil pH “5.6–6.5” after biochar application. This is mainly because soil pH is strongly associated with soil CEC [100]. As soil pH decreases, it becomes acidic, and the number of negative charges on the colloids decreases [101], thereby decreasing potential CEC. LASSO also identified publication year as a predictor with a negative impact on the response.

##### Effective CEC

**Fig. 16:**
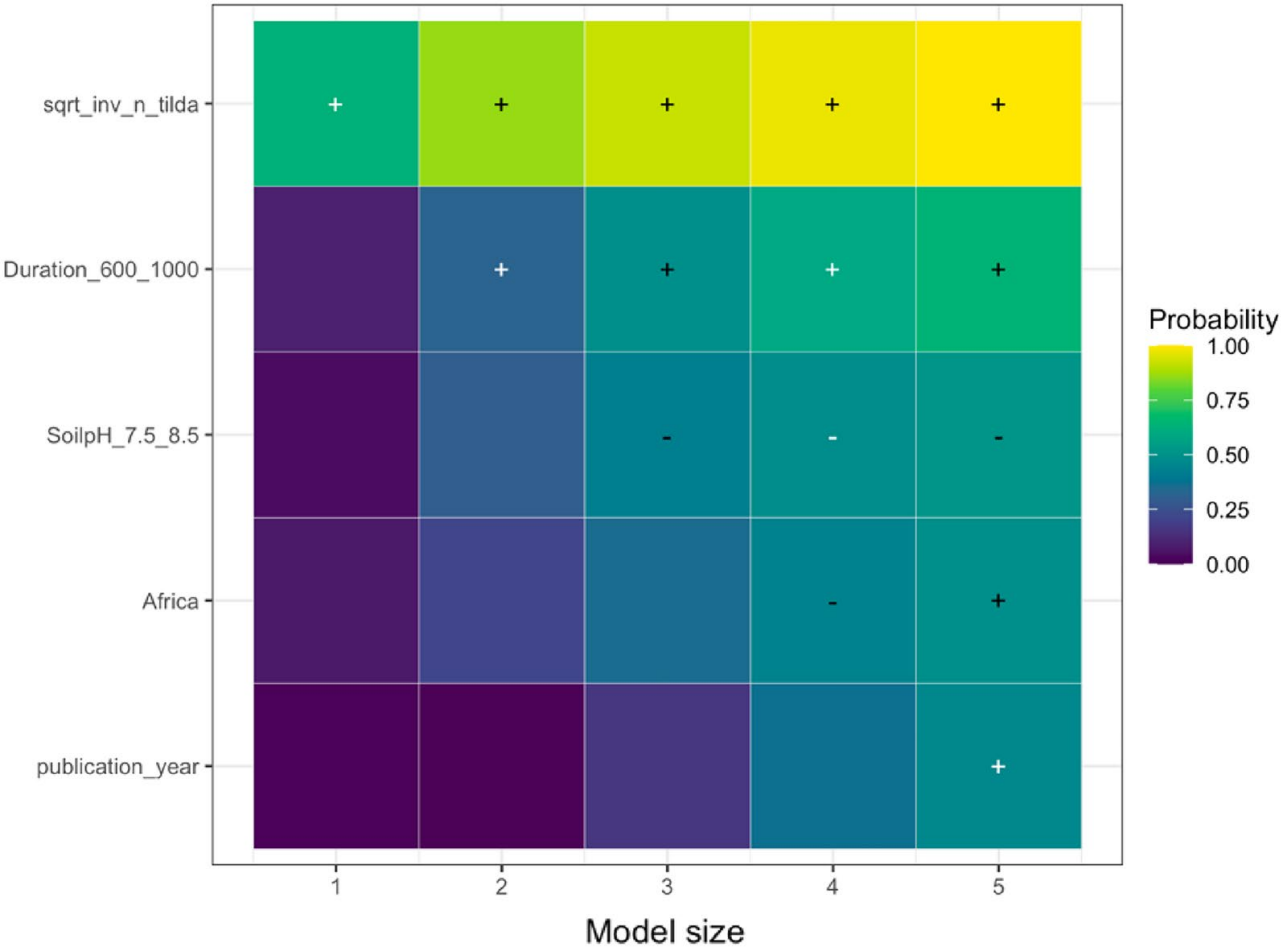
The results of randomized LASSO on the key predictors in effective CEC response. The importance of a variable is quantified by running the LASSO model for five different model sizes (1–5 regressors). This visualization shows the variables selected in a minimum of one out of five LASSO models

Randomized LASSO models selected different predictors as important, such as experimental country, duration, soil pH, and publication year (Table 7). The square root of effective sample size emerged as the most influential, with a positive effect on the response of effective CEC (Fig. 16), suggesting a likelihood of publication bias. The results show that effective CEC is positively affected at longer experimental durations (Fig. 16). This can be attributed to the aging processes that biochar undergoes over time, which may increase its ability to retain and exchange cations [102]. Furthermore, microbial activity plays a crucial role in altering the surface chemistry of biochar [103], improving its ability to retain cations and consequently increasing CEC. The response of effective CEC yielded an intriguing result by showing a negative trend in soils with higher pH levels (7.5–8.5). This outcome may be attributed to variations in biochar properties and application rates. In Africa, the effect of biochar on effective CEC has yielded inconclusive results, with both positive and negative outcomes. Further research is required to clarify the impact of biochar on effective CEC in sandy soils in this region. Furthermore, the selection of publication year as an important predictor with a positive effect suggests a positive shift in the response over time.

##### Mineral N leaching

**Fig. 17:**
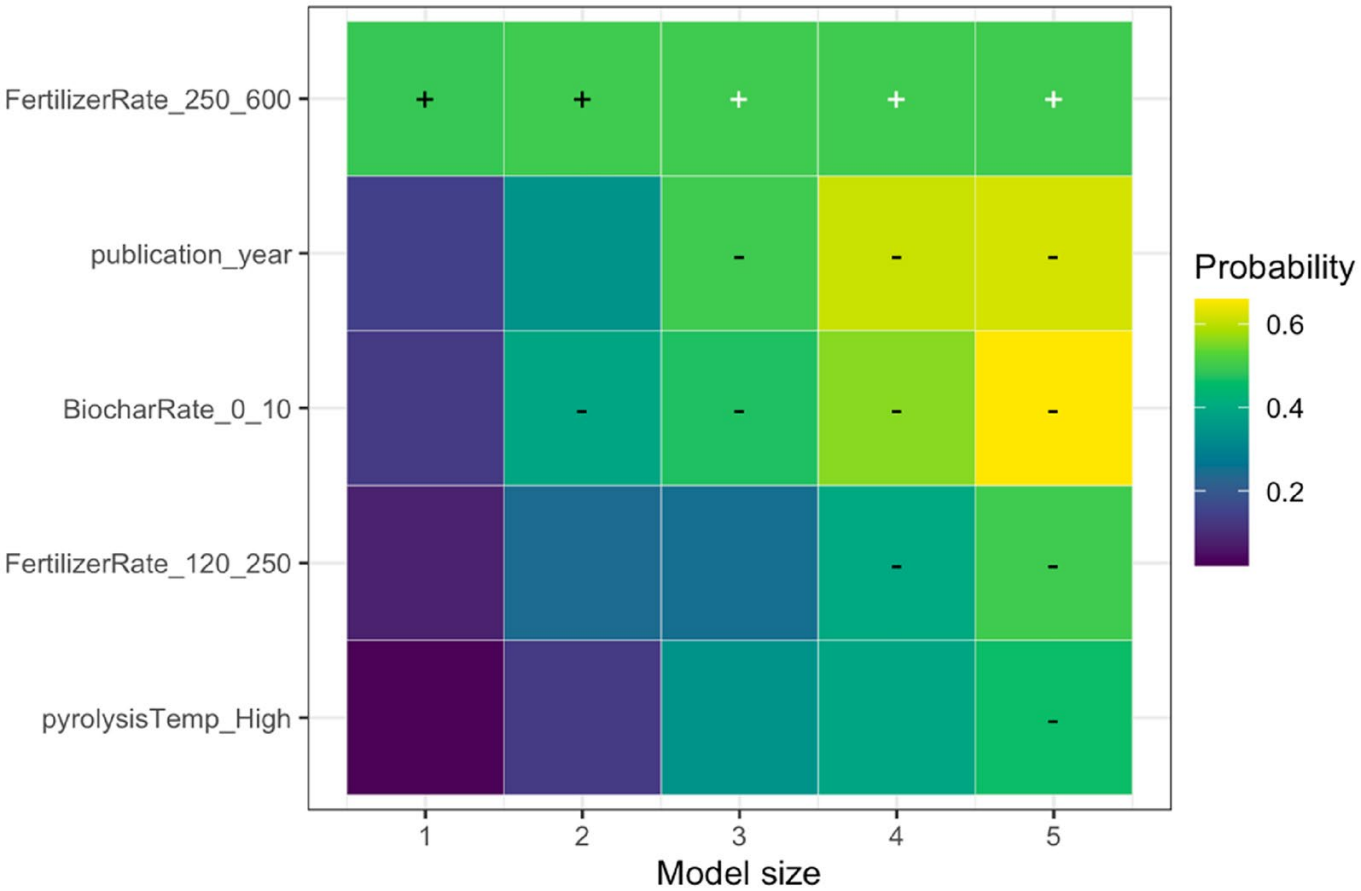
The results of randomized LASSO on the key predictors in mineral N leaching response. The importance of a variable is quantified by running the LASSO model for five different model sizes (1–5 regressors). This visualization shows the variables selected in a minimum of one out of five LASSO models

The key predictors contributing to the heterogeneity in the overall response of mineral N leaching to biochar application were found to be the biochar application rate, pyrolysis temperature, fertilizer application rate with biochar, and publication year. Higher fertilizer rates with biochar increased mineral N leaching, while lower rates had the opposite effect (Fig. 17). The influence of higher fertilization rates on nutrient leaching depends on the balance between nutrient uptake by plants and nutrient losses due to leaching. At high fertilizer levels, plants may not be able to utilize all of the nutrients applied, resulting in a surplus that may leach into groundwater. Our analysis indicated that low biochar application rates reduced mineral N leaching (Fig. 17), although other meta-analyses have demonstrated the benefits of higher biochar application rates [89]. This discrepancy may be attributed to the surface properties of biochar, which enable it to adsorb ions in the soil solution [89]. The electrostatic and capillary forces on the biochar surface contribute to reducing nutrient leaching from soils. Soils amended with biochar can adsorb ${{\text{NO}}}_{3}^{-}$ through its anion exchange sites, thus decreasing nitrogen losses. Additionally, biochar may increase soil water-holding capacity due to its large specific surface area and high porosity, reducing soil water percolation and the nitrogen contained in it [104] [105]. As per the analysis, biochar produced at high temperatures reduced the response to mineral N leaching, which may be due to the ability of biochar at high temperatures to retain ${{\text{NO}}}_{3}^{-}$-${\text{N}}$ effectively, preventing its leaching into groundwater [30]. Nevertheless, our findings are in contrast to the results presented by Liu, Zhang [90], who suggest the potential advantages of low-pyrolyzed biochar for reducing N leaching. Our study specifically examines biochar's ability to mitigate N leaching in sandy-textured soils, so direct comparisons with other studies may not be appropriate. Publication year was another predictor identified in our analysis (Fig. 17), and it showed a decreasing trend in response over the years.

## Review limitations

### Limitations of the review methods

Throughout the systematic review processes, we sought to minimize potential biases regarding the review methodology. The regular consultations with the advisory committee and other experts facilitated the identification of relevant and reliable studies and the reduction of likely biases. We created comprehensive search strings with the aim of applying them to diverse search sources. However, the full search strings provided in Table 2 could only be used in some bibliographic databases. When applied to others, such as NDLTD (Networked Digital Library of Theses and Dissertations) and OATD (Open access theses and dissertations), simplified search strings had to be used (Additional file 2; Table 7) and the same was true for all organization websites. Hence, we were forced to simplify the search string to "Biochar OR Charcoal OR Agrichar" for most of them (Additional file 2: Table S8). However, compared to other systematic reviews, the number of sources searched in this review was considerable [8, 38, 39]. Therefore, we argue that the existence of publication bias due to a lack of comprehensiveness is negligible. Our review was limited to the English language and, although most of the peer-reviewed journals were in English, we discovered during the screening process that some peer-reviewed and gray literature was published in other languages. However, at the full-text screening stage, only two articles (out of 1137) were found to be ineligible due to language. This result indicates that there is a very small chance of language bias.

During both screening stages (title and abstract and full-text screening), all studies were independently screened. In cases where there was uncertainty about excluding studies, both reviewers worked together to discuss and reach a consensus. The implementation of the screening procedures independently aimed to mitigate potential limitations linked to the screening process, including the risk of biased decisions, and to enhance the overall robustness of the screening.

As mentioned in the section on deviations from the protocol, we applied text-mining after the title and abstract screening to reduce the number of studies for full-text screening. Although the text-mining followed rigid procedures, setting cut-off thresholds to exclude irrelevant papers may have led to the exclusion of some relevant studies as well [106]. We tried to minimize this bias by manually assessing 10% of the studies excluded at text-mining (randomly selected), which suggests there were few or no falsely excluded studies.

It was also sometimes necessary to transform the data when there were several potential interventions or comparators in order to prevent duplicate extractions or dependent data. For example, we averaged data when the data were provided for several years, months, or days. However, the effects of biochar application to the soil may have been different over time.

### Limitations of the evidence base

Many articles (303) were excluded due to low validity or high risk of bias, mainly because of performance, sampling, and selection bias. Performance bias was the dominant reason for attributing a high risk of bias, mainly because studies were not based on randomization. Randomization decreases the likelihood of confounding factors [107]. When the intervention and comparison sites were not well matched, or when the sampling method was not appropriate to collect data on the population of interest, the studies struggled with the risk of selection bias or improper sampling. In this case, studies were excluded from the review. One hundred and seventy publications were attributed a ‘moderate’ risk of bias, either because most of the effect modifiers were missing (Fig. 2) or there was the risk of selection bias due to a mismatch between treatment and control. Based on the review protocol, both studies with ‘low’ and ‘moderate’ risk of bias were supposed to be used for the data extraction. However, only the former was included for data extraction and in the analyses, while the latter was excluded after discussion with the advisory committee. Due to the significant number of ‘low’ risks of bias studies, our focus remained on this category. The significant number of studies with a 'moderate' and a 'high' risk of bias prevented us from extracting data during the review, thus not allowing the implementation of additional sensitivity analyses. It is possible that some relevant studies were lost as a result of this decision. Also, the outcome might have been different if the studies with a ‘moderate’ and ‘high’ risk of bias had been included. However, studies with a ‘moderate’ and ‘high’ risk of bias could have introduced bias and caused missing data. Therefore, we believe such studies would have been excluded from the quantitative syntheses and, hence, we believe it is reasonable to assume the overall effect of biochar on SPPs would not change and results obtained without bias accurately reflect the true effect of biochar on SPPs.

### Limitations in generalizing the results

Of the 109 articles included in this review, 17 were excluded at the data extraction stage and they were not included in both qualitative and quantitative synthesis largely due to data readability issues (the data were not readable with webplotdigitizer). Other than stated in the protocol [44], we only used the 92 ‘low’ risk of bias articles for both quantitative and qualitative synthesis, as we believed this could ensure consistency and comparability in the analyses. The overall effect of biochar on nine SPPs was computed in quantitative synthesis (Fig. 8) and we observed that biochar had a significant positive effect on soil total NPK, plant nutrient level, NPK availability, N_2_O emission, potential CEC, and mineral N leaching. However, the number of articles and observations among these SPPs differed. For example, soil total NPK had the highest number of articles and observations (61 articles and 505 observations), while mineral N leaching had a lower number of articles and observations (5 articles and 44 observations). Nevertheless, prior review studies have found that biochar addition to sandy-textured soils decreased N leaching by 44% [8], so we deem our results regarding the reduction of mineral N leaching to be reliable. Biochar had no influence on soil mineral nitrogen and NUE of sandy-textured soils. However, the number of observations for NUE in this review was relatively low. Therefore, it is possible that with a higher number of observations, an effect may have been found. Although NUE was not affected by biochar treatment in this study, food crop yields showed a contrasting response to biochar application. Again, this could be due to the lack of studies for NUE in this review, which future studies should address. However, for soil mineral nitrogen, the number of studies and observations was large enough, so we can say with reasonable certainty that biochar has no effect on it.

The PET-PEESE method we used to correct for publication bias is one of the recommended techniques for ecological review studies [41, 64]. However, PET-PEESE may perform poorly when the number of included studies is small (< 20) and heterogeneity is very high. The number of unique articles recorded for some of the SPPs violated this rule, but the number of observations exceeded 20 for all SPPs (Additional file 13). Therefore, we assume that PET-PEESE is the right approach for our case. After correction for publication bias, effective CEC of sandy-textured soils did not appear to benefit from biochar treatment. To further our understanding of the potential benefits of biochar on effective CEC of sandy-textured soils, future review studies should explore this topic more, using a larger number of observations.

Estimating the variance based on the pooled CV when SD or SE was not given may raise concerns about the credibility of the results. A sensitivity analysis was performed by removing all observations from these variances based on the pooled CV and the results differed considerably, as almost two-thirds of the observations were excluded from the analysis (Additional file 11). However, Nakagawa, Noble [60] proposed this method as one of the approved methods when the variance could not be calculated in a straightforward way. Furthermore, using this method meant we could avoid losing valuable studies for our review.

## Conclusion

According to our results, the nutrient cycle of sandy-textured soils is changed by biochar application by increasing soil total NPK, plant nutrient level, NPK availability and potential CEC. At the same time, biochar application decreases N_2_O emissions and mineral N leaching. The results are also in reasonable agreement with previously performed meta-analyses [8, 38, 39]. However, we have focused on the effects of biochar on the nutrient cycle of sandy-textured soils, with the ability to compare different SPPs. In addition to SPPs, an increase in food crop yields is noted, while biomass production was not affected. The limited number of articles and observations for certain SPPs, such as N_2_O emissions, potential CEC, NUE, and mineral N leaching, limits the ability to draw strong conclusions about their effects.

The change in these SPPs after biochar application depends on a number of factors. For example, in particular, experimental continent, biochar application rate, soil pH, and publication year (Table 7) were the predictors that explained the heterogeneity in the response of many SPPs to biochar application. Unfortunately, randomized LASSO was not feasible for analyzing NUE due to insufficient observations, which could compromise the reliability of the results. Some predictors were not selected for SPP responses, likely due to missing values or their limited impact. Variables like average precipitation, annual temperature, and fertilizer application before or after biochar lacked variability, making their effect on the overall response uncertain. LASSO results also showed that soil management practices during and after biochar addition, like tillage and irrigation, had minimal impact on SPP responses. The key strength of our systematic review, aiming to provide globally applicable outcomes, faced a hurdle due to the uneven distribution of observations across different regions.

### Implications for management and policy

Policy discussions on biochar have shown hesitancy, primarily arising from the prevailing uncertainty regarding biochar’s effects on ecosystem services [109]. Prior investigations into biochar’s role in the soil nutrient cycle highlighted the variability of relationships between some key components (N_2_O emission, N leaching, plant N uptake, and soil NH_3_ volatilization) due to diverse soil and biochar characteristics [8, 38, 39]. However, these studies did not specifically address the impact of biochar on the nutrient cycle in sandy-textured soils. While some of our findings align with previous reviews, our review distinguishes itself through its systematic approach and the provision of the most recent findings. According to our review, biochar offers a suite of benefits for addressing the nutrient cycle problem. From enhancing nutrient retention and soil fertility to reducing nutrient runoff and greenhouse gas emissions, biochar can play an important role in promoting sustainable nutrient management practices in future agriculture. In practice, the potential generalizability of our findings regarding biochar’s impact on nutrient dynamics of sandy-textured soils functions as a strategic guide for agricultural policymakers and farmers, assisting their decision-making that addresses the complexities of biochar, such as different biochar types, soil management during and after biochar application, and optimal biochar application rates.

More specifically, the findings of this review provide policymakers with valuable insights into the potential of integrating biochar into agricultural practices offering a sustainable approach to mitigating the impacts of nitrate pollution on both a local and global scale. Based on our findings, biochar application diminishes N_2_O emissions, particularly when employing a shortened experimental duration alongside a moderate biochar application rate. Additionally, a lower rate of biochar application has demonstrated a notable decrease in mineral N leaching from sandy-textured soils. These abilities of biochar showcase not only its potential to remedy environmental pollution but also its promising offer to address groundwater pollution in various countries [108]. This review highlights biochar's ability to stimulate NPK availability in coarse-textured soils and intensify overall NPK content and potential CEC in sandy-textured soils at higher application rates. These findings not only show that biochar has potential to improve environmental sustainability but also underscores biochar’s promising potential in contributing to agricultural productivity.

### Implications for future research

The geographical spread of the studies included in this review revealed that more research is needed on the effect of biochar on the nutrient cycling of sandy-textured soils. In general, the evidence is especially lacking in continents such as North and South America and Oceania (Additional file 10: Table S10.1). Relevant experimental studies were lacking for mineral N leaching, NUE, and potential and effective CEC of sandy-textured soils. Moreover, most experiments were carried out on either sandy soil or sandy loam, while very few studies were conducted on coarse loamy, coarse sand, and loamy soil. Unfortunately, some predictors such as climate type, compost application with biochar, or before biochar could not be included in the analyses due to the large amount of missing data in all SPPs. Therefore, future research should focus on filling these gaps. We encourage future experimental studies to incorporate true replication and randomization methods to enhance the generalizability of their findings. Finally, we recommend that the research community presents the results in a readable form of figures and tables to further facilitate the use of data in meta-analyses.

Given the complexity of studying all components of the soil nutrient cycle in one review study, it is likely that the response of other nutrient cycle components besides SPPs has not been specifically examined in this study. For instance, the responses of the microbial activity or soil mineralization to biochar application in sandy-textured soils, which are important indicators of soil quality and components of soil nutrient cycles [110, 111], are missing in this review. Besides, biochar characteristics such as biochar porosity, its CEC, and its specific surface area can significantly affect the nutrients of sandy-textured soils [31, 112], which have not been investigated in this review. Furthermore, plant and/or crop ID was not included in the analysis of this study. Thus, future review studies could address these missing components of the soil nutrient cycle and biochar features to complement this review.

## Supplementary Information

**Additional file 1. **ROSES systematic review report.

**Additional file 2. **Search strategy and results. Provides a description of the search strategy and results of the literature searches. For each source, we provided full details on the search date(s), search strings used, search settings and restrictions, subscriptions (if applicable), and the number of returns.

**Additional file 3. **Papers excluded after text-mining. Provided further keywords used in text-mining.

**Additional file 4. **The results of full-text screening included both excluded and included articles. Separate lists of unobtainable articles and duplicates in full-text screening.

**Additional file 5. **Adjusted critical appraisal criteria.

**Additional file 6. **The results of critical appraisal. Provides the critical assessment criteria and evaluation of each paper.

**Additional file 7. **Updated data coding table for data extraction.

**Additional file 8. **Statistical analysis of variation in the results of different measurement methods of SPPs.

**Additional file 9. **Data-extraction sheet. Contains the coding of extracted data for all articles included in both qualitative and quantitative synthesis.

**Additional file 10. **Descriptive statistics. Contains further descriptions of data for narrative synthesis.

**Additional file 11. **Sensitivity analyses. The effect of outlier removal and estimated SD from P value removal on the total outcome.

**Additional file 12. **Meta-regression results. Contains the results of LASSO analyses for each SPP.

**Additional file 13. **Correction for publication bias. Contains funnel plots for each SPP and PET-PEESE results.

**Additional file 14. **Consistency check results for all screening stages (title & abstract screening and full-text screening) and critical appraisal.

**Additional file 15. **R code. Contains all codes used for meta-analysis, meta-regression and correction for publication bias.

## Data Availability

All data analyzed during this study are included in this published article and its Additional files.

## Abbreviations

NUE: Nutrient use efficiency
CEC: Cation exchange capacity
SPPs: Soil and plant properties
NPK: Nitrogen-phosphorus-potassium
SD: Standard deviation
SE: Standard error
