# Supplementary material for: Biochar improves the nutrient cycle in sandy-textured soils and increases crop yield: a systematic review
Source: Environ Evid. 2024 Feb 22;13:3. doi: 10.1186/s13750-024-00326-5 (PMC11376106; doi:10.1186/s13750-024-00326-5)
Supplement: Supplementary file 2 — Additional file 2. Search strategy and results. Provides a description of the search strategy and results of the literature searches. For each source, we provided full details on the search date(s), search strings used, search settings and restrictions, subscriptions (if applicable), and the number of returns. [file 13750_2024_326_MOESM2_ESM.docx]

**Search sources and results**

**Table 2.1.** Databases searchesand results

| Web of Science “Core collection” | Searched 06/04/2021, 2410 results. Done automatically |
| --- | --- |
| Scopus: | Searched 06/04/2021, 2458 results. Done automatically |
| AGRICOLA | Searched 06/04/2021, 1612 results (Title based). Done automatically |
| AGRIS | Searched 21/04/2021, 36 results, 7 of them dublicates and 1 is not related – 28 of them, all done manually |
| ProQuest Environmental Sciences and Pollution Management | Searched 21/04/2021, 829 results, all done manually |
| EBSCO Open Dissertations | Searched 22/04/2021, 4407 results, all done manually |
| Networked Digital Library of Theses and Dissertations | Searched 23/04/2021, 481 results, but 44 of them were dublicates and 157 were not related, only related ones were 280, all done manually |
| Open access theses and dissertations | Searched 27/05/2021, 582 results; 392 results were not related, 4 results were not found, 167 results were extracted manually. |
| **Total** | **12191** |

**Table 2.2.** Organizational website search

| International biochar initiative | Searched 03/05/2021, found 7 results, done manually |
| --- | --- |
| UK Biochar research center | Searched 03/05/2021, found 50 results, done manually |
| US Biochar initiative | Searched 03/05/2021, no result |
| The European biochar certificate | Searched 03/05/2021, no result |
| Sanoma Biochar Initiative | Searched 03/05/2021, 22 results |
| Israel Biochar Research Network | Searched 03/05/2021, 21 results |
| Biochar for sustainable soils | Searched 03/05/2021, no related results |
| Ithaka Institute | Searched 03/05/2021, 36 results |
| New Zealand Biochar Research Center | Searched 03/05/2021, no related results |
| Environmental Protection Agency (USA) | Searched 03/05/2021, 10 results |
| Research Institute for Organic Agriculture | Searched 03/05/2021, no related results |
| Research Institute for Agriculture, Fisheries and Food (ILVO, Belgium, Flanders) | Searched 03/05/2021, 3 results |
| Netherlands Organization for Applied Scientific Research | Searched 03/05/2021, 7 results |
| Wageningen University & Research (The Netherlands) | Searched 03/05/2021, 98 results |
| Julius Kühn Institute—Federal Research Centre for Cultivated Plants (Germany): | Searched 03/05/2021, 8 results |
| Mercator Research Institute on Global Commons and Climate Change (Germany): | Searched 03/05/2021, 4 results |
| Thünen institute—Federal Research Institute for Rural Areas, Forestry and Fisheries (Germany): | Searched 03/05/2021, 3 results |
| Agroscope (Switzerland) | Searched 03/05/2021, 20 results |
| James Hutton Institute (United Kingdom): | Searched 03/05/2021, 11 results |
| Rothamsted Research (United Kingdom): | Searched 04/05/2021, no related results |
| **Total** | **300** |

**Table 2.3.** Web-based search

| Google scholar | Searched 20/05/2021, 2600 results |
| --- | --- |

**Table 2.4.** Search queries for databases searches

| ***Web of Science*** |
| --- |
| (TS = (biochar* OR "biochar amendment$" OR agrichar OR "black carbon$" OR "biochar-mediated" OR charcoal OR "biochar field study")) AND (TS = ("hydrological control*" OR "hydraulic properties*" OR "hydrologic* cycle" OR “water retention” OR “clean water” OR “hydrological control” OR “water leak*” OR “water leach*” OR "water availabl*" OR "soil water availability" OR "readily avail- able water" OR evaporation OR "water runoff" OR "plant avail- able water" OR "WHC" OR "soil humidity" OR "field capacity" OR "nutrient loss" OR "soil moisture" OR "water holding capacity" OR "water content" OR "water infiltration" OR "water cycle" OR "nutrient availabl*" OR "available nutrient$" OR nutrient$ OR "nutrient cycle" OR "nitrogen availabil*" OR "nutrient exchange capacit*" OR "available nitrogen" OR NPK OR "nutrient leak*" OR “nitrogen leach*” OR “phosphorus leach*” OR “potassium leach*” OR "nutrient leach*" OR "soil nutrients" OR "available phospho- rus" OR "phosphorus availabil*" OR "available potassium" OR "potassium availabil*" OR "climate regula*" OR "climate mitiga- tion" OR "climate change mitigation" OR "climate chang*" OR "global warming" OR “carbon sequestration” OR "soil carbon sequestration" OR “carbon capture and storage” OR "carbon capture" OR "emission$" OR "CCS" OR “ammonia emission$*” OR “methane emission$*” OR "nitrate emission$" OR "nitrous-oxide emission$" OR "NH4" OR "NH3" OR "CH4" OR "N2O" OR "CO2" OR "greenhouse gas emission$" OR "crop growth" OR "crop yield" OR "crop produc- tion" OR "biomass growth" OR "biomass yield" OR "biomass production" OR "raw material" OR "plant growth" OR "field" OR yield OR "increase" OR "improve*" OR "decrease" OR "mitiga*" OR "growth" OR "alleviat*" OR "effect$" OR "impact")) AND (TS = ("sand* soil$" OR "sandy soil" OR “sand* loamy soil$” OR “loamy sandy soil” OR “contaminated* soil$” OR "heavy metal* soil$" OR "sandy loam soil" OR "sandy loam" OR ultisol OR "agricultural soil" OR "loamy sand" OR "sandy clay loam" OR "cropping system" OR “sandy calcareous soil”)) |
| ***Scopus*** |
| TITLE-ABS-KEY ( biochar*  OR  "biochar amendment$"  OR  agrichar  OR  "black carbon$"  OR  "biochar-mediated"  OR  charcoal  OR  "biochar field study" )  AND  TITLE-ABS-KEY ( "hydrological control*"  OR  "hydraulic properties*"  OR  "hydrologic* cycle"  OR  "water retention"  OR  "clean water"  OR  "hydrological control"  OR  "water leak*"  OR  "water leach*"  OR  "water availabl*"  OR  "soil water availability"  OR  "readily avail- able water"  OR  evaporation  OR  "water runoff"  OR  "plant avail- able water"  OR  "WHC"  OR  "soil humidity"  OR  "field capacity"  OR  "nutrient loss"  OR  "soil moisture"  OR  "water holding capacity"  OR  "water content"  OR  "water infiltration"  OR  "water cycle"  OR  "nutrient availabl*"  OR  "available nutrient$"  OR  nutrient$  OR  "nutrient cycle"  OR  "nitrogen availabil*"  OR  "nutrient exchange capacit*"  OR  "available nitrogen"  OR  npk  OR  "nutrient leak*"  OR  "nutrient leach*"  OR  "soil nutrients"  OR  "available phosphorus"  OR  "phosphorus availabil*"  OR  "available potassium"  OR  "potassium availabil*"  OR  "climate regula*"  OR  "climate mitigation"  OR  "climate change mitigation"  OR  "climate chang*"  OR  "global warming"  OR  "carbon sequestration"  OR  "soil carbon sequestration"  OR  "carbon capture and storage"  OR  "carbon capture"  OR  "emission$"  OR  "CCS"  OR  "ammonia emission$*"  OR  "methane emission$*"  OR  "nitrate emission$"  OR  "nitrous-oxide emission$"  OR  "NH4"  OR  "NH3"  OR  "CH4"  OR  "N2O"  OR  "CO2"  OR  "greenhouse gas emission$"  OR  "crop growth"  OR  "crop yield"  OR  "crop produc- tion"  OR  "biomass growth"  OR  "biomass yield"  OR  "biomass production"  OR  "raw material"  OR  "plant growth"  OR  "field"  OR  yield  OR  "increase"  OR  "improve*"  OR  "decrease"  OR  "mitiga*"  OR  "growth"  OR  "alleviat*"  OR  "effect$"  OR  "impact" )  AND  TITLE-ABS-KEY ( "sand* soil$"  OR  "sandy soil"  OR  "sand* loamy soil$"  OR  "loamy sandy soil"  OR  "contaminated* soil$"  OR  "heavy metal* soil$"  OR  "sandy loam soil"  OR  "sandy loam"  OR  ultisol  OR  "agricultural soil"  OR  "loamy sand"  OR  "sandy clay loam"  OR  "cropping system"  OR  "sandy calcareous soil" )  AND  ( LIMIT-TO ( LANGUAGE ,  "English" ) ) |
| ***Agricola*** |
| (Biochar) AND (Hydrological Control Hydraulic Properties Hydrological Cycle Water Retention Clean Water Hydrological Control Water Leak Water Leach Water Availability Evaporation Evapotranspiration Water Runoff WHC Soil Humidity Field Capacity Nutrient Loss Soil Moisture Water Holding Capacity Water Content Water Infiltration Water Cycle Nutrient Availabl Available Nutrient Nutrient? Nutrient Cycle Nitrogen Availabil Nutrient Exchange Capacity Available Nitrogen NPK Nutrient Leak Nutrient Leach Soil Nutrients Available Phosphorus Phosphorus Availabil Available Potassium Potassium Availabil Climate Regula Climate Mitigation Climate Change Mitigation Carbon Sequestration Carbon Capture Emission Ammonia Emission Methane Emission Nitrate Emission Nitrous-Oxide Emission NH4 NH3 CH4 N2O CO2 Greenhouse Gas Emission Crop Growth Crop Yield Crop Production Biomass Growth Biomass Yield Biomass Production Raw Material Plant Growth Field Yield) AND (Sandy soil Sandy Loam? Loamy Sand? Comtaminated Soil?)   - Title to all was selected to all search bars |
| ***Agris*** |
| (Biochar OR (Biochar AND Amendment) OR Agrichar OR Charcoal OR (BlackAND Carbon)) AND ((Hydrological AND Control) OR (Hydrological AND Cycle) OR (Water AND Retention) OR(Clean AND Water) OR (Water AND Leak) OR(Water AND Leach) OR (Water AND Availability) OR Evaporation OR (Water AND Runoff) OR(Nutrient AND Loss) OR (Soil AND Moisture) OR (Water-holding ANDCapacity) OR (Nutrient AND Availability) OR (Available AND Nutrient) OR Nutrient OR (Nutrient AND Cycle) OR(Available AND Nitrogen) OR (Nutrient AND Leaking) OR (Nutrient ANDLeaching) OR (Available AND Phosphorus) OR(Phosphorus AND Availability) OR (Climate AND Regulation) OR (ClimateAND Mitigation) OR (Climate-change AND Mitigation) OR Climate* OR(Global AND Warming) OR (Carbon AND Sequestration) OR (Carbon AND Capture) OR Emission OR (Crop AND Yield) OR (Crop AND Production) OR (Biomass AND Yield) OR (Biomass AND Production) OR (Raw AND Material) OR (Plant AND Growth)) AND ((Sandy AND Soil) OR (Sandy AND Loamy AND Soil) OR (Loamy AND Sandy) OR (Contaminated AND Soil) OR (Sandy AND Loam) OR (Sandy AND Loam)) |
| ***ProQuest*** |
| (SU = (biochar* OR biochar amendment OR agrichar OR black carbon OR charcoal)) AND (SU = (hydrological control OR hydraulic properties OR hydrological cycle OR water retention OR clean water OR hydrological control OR water leak OR water leach OR water availabl OR soil water availability OR readily available water OR evaporation OR water runoff OR plant available water OR WHC OR soil humidity OR field capacity OR nutrient loss OR soil moisture OR water holding capacity OR water content OR water infiltration OR water cycle OR nutrient availabl* OR available nutrient OR nutrient OR nutrient cycle OR nitrogen availabil* OR nutrient exchange capacit* OR available nitrogen OR NPK OR nutrient leak* OR nutrient leach* OR soil nutrients OR available phosphorus OR phosphorus availabil* OR available potassium OR potassium availabil* OR climate regula* OR climate mitigation OR climate change mitigation OR climate change OR global warming OR carbon sequestration OR soil carbon sequestration OR carbon capture and storage OR carbon capture OR emission OR CCS OR ammonia emission OR methane emission OR nitrate emission OR nitrous-oxide emission OR NH4 OR NH3 OR CH4 OR N2O OR CO2 OR greenhouse gas emission OR crop growth OR crop yield OR crop production OR biomass growth OR biomass yield OR biomass production OR raw material OR plant growth OR field OR yield OR increase OR improve* OR decrease OR mitiga* OR growth OR alleviat* OR effect OR impact)) AND (SU = (sand* soil OR sandy soil OR sand* loamy soil OR loamy sandy soil OR contaminated* soil OR heavy metal* soil OR sandy loam soil OR sandy loam OR ultisol OR agricultural soil OR loamy sand OR sandy clay loam OR cropping system OR sandy calcareous soil)) |
| ***EBSCO*** |
| (TI(Biochar OR “Biochar amendment” OR Agrichar OR “Black carbon” OR Charcoal)) AND (TI(“Hydrological control” OR “Hydraulic properties” OR “Hydrological cycle” OR “Water retention” OR “Clean water” OR “Hydrological control” OR “Water leak” OR “Water leach” OR “Water availabl*” OR Evaporation OR “Water runoff” OR “Soil humidity” OR “Field capacity” OR “Nutrient loss” OR “Soil moisture” OR “Water holding capacity” OR “Water content” OR “Water infiltration” OR “Water cycle” OR “Nutrient availability” OR “Available nutrient” OR Nutrient* OR “Nutrient cycle” OR “Nitrogen availabil*” OR “Nutrient exchange capacit*” OR “Available nitrogen” OR NPK OR “Nutrient leak*” OR “Nutrient leach*” OR “Soil nutrients” OR “Available phosphorus” OR “Phosphorus availabil*” OR “Available potassium” OR “Potassium availabil*” OR “Climate regula*” OR “Climate mitigation” OR “Climate change mitigation” OR “Climate change” OR “Global warming” OR “Carbon sequestration” OR “Soil carbon sequestration” OR “Carbon capture and storage” OR “Carbon capture” OR Emission* OR “Ammonia emission” OR “Methane emission” OR “Nitrate emission” OR NH4 OR NH3 OR CH4 OR N2O OR CO2 OR “Greenhouse gas emission” OR “Crop growth” OR “Crop yield” OR “Crop production” OR “Biomass growth” OR “Biomass yield” OR “Biomass production” OR “Raw material” OR “Plant growth” OR Field* OR Yield* OR Increase OR Improve* OR Decrease OR Mitiga* OR Growth OR Alleviat* OR Effect OR Impact)) AND (TI “Sandy soil” OR “Sandy loamy soil” OR “Loamy sandy soil” OR “Contaminated soil” OR “Heavy metal soil” OR “Sandy loam soil” OR “Sandy loam” OR Ultisol OR “Loamy sand”)) |
| ***NDLTD*** |
| (subject: "biochar" OR "Charcoal" OR "Black carbon") AND (subject: "Water*" OR "Climate*" OR "Carbon*" OR "Sequestration" OR "Nutrient*" OR "Nitrogen*" OR "Crop*" OR "Biomass*" OR "Increase" OR "Decrease") |
| ***OATD*** |
| “biochar OR charcoal OR agrichar” |

**Table 2.5.** Search queries for organizational website searchers

| ***International biochar initiative*** |
| --- |
| “biochar OR charcoal OR agrichar” |
| ***UK Biochar research center*** |
| “biochar OR charcoal OR agrichar” |
| ***US Biochar initiative*** |
| “biochar OR charcoal OR agrichar” |
| **The European biochar certificate** |
| “biochar OR charcoal OR agrichar” |
| **Sanoma Biochar Initiative** |
| “biochar OR charcoal OR agrichar” |
| **Israel Biochar Research Network** |
| “biochar OR charcoal OR agrichar” |
| **Biochar for sustainable soils** |
| “biochar OR charcoal OR agrichar” |
| **Ithaka Institute** |
| “biochar OR charcoal OR agrichar” |
| **New Zealand Biochar Research Center** |
| (biochar OR charcoal OR black carbon) AND (crop yield OR biomass OR climate OR water OR nutrient) |
| **Environmental Protection Agency (USA)** |
| “biochar OR charcoal OR agrichar” |
| **Research Institute for Organic Agriculture** |
| “biochar OR charcoal OR agrichar” |
| **Research Institute for Agriculture, Fisheries and Food (ILVO, Belgium, Flanders)** |
| “biochar OR charcoal OR agrichar” |
| **Netherlands Organization for Applied Scientific Research** |
| “biochar OR charcoal OR agrichar” |
| **Wageningen University & Research (The Netherlands)** |
| “biochar OR charcoal OR agrichar” |
| **Julius Kühn Institute—Federal Research Centre for Cultivated Plants (Germany):** |
| “biochar OR charcoal OR agrichar” |
| **Mercator Research Institute on Global Commons and Climate Change (Germany):** |
| “biochar OR charcoal OR agrichar” |
| **Thünen institute—Federal Research Institute for Rural Areas, Forestry and Fisheries (Germany):** |
| “biochar OR charcoal OR agrichar” |
| **Agroscope (Switzerland)** |
| “biochar OR charcoal OR agrichar” |
| **James Hutton Institute (United Kingdom):** |
| “biochar OR charcoal OR agrichar” |
| **Rothamsted Research (United Kingdom):** |
| “biochar OR charcoal OR agrichar” |

**Table 2.6.** Web-based searches

| **Google scholar – 2600 results** |
| --- |
| Allintitle: Biochar + (Hydrological Control \| Hydraulic Properties \| Hydrological Cycle \| Water Retention \| Clean Water \| Hydrological Control \| Water Leak \| Water Leach \| Water Availability \| Evaporation \| Evapotranspiration \| Water Runoff \| WHC \| Soil Humidity \| Field Capacity \| Nutrient Loss \| Soil Moisture \| Water Holding Capacity \| Water Content \| Water Infiltration \| Water Cycle \| Nutrient Availability \| Nutrient \| Nutrient Cycle \| Nitrogen Availability \| Nutrient Exchange Capacity \| Available Nitrogen \| NPK \| Nutrient Leak \| Nutrient Leach \| Soil Nutrients \| Available Phosphorus \| Phosphorus Availability \| Available Potassium \| Climate Regulation \| Climate Mitigation \| Climate Change \| Mitigation Carbon Sequestration \| Carbon Capture \| Emission \| Ammonia \| Methane \| Nitrate \| Nitrous-Oxide \| NH4 \| NH3 \| CH4 \| N2O \| CO2 \| Greenhouse Gas \| Crop Growth \| Crop Yield \| Crop Production \| Biomass Growth \| Biomass Yield \| Biomass Production \| Raw Material \| Plant Growth \| Field Yield) + (Sandy soil \| Sandy Loam \| Loamy Sand \| Comtaminated Soil)) |
