# Supplementary material for: Biochar improves the nutrient cycle in sandy-textured soils and increases crop yield: a systematic review
Source: Environ Evid. 2024 Feb 22;13:3. doi: 10.1186/s13750-024-00326-5 (PMC11376106; doi:10.1186/s13750-024-00326-5)
Supplement: Supplementary file 5 — Additional file 5. Adjusted critical appraisal criteria. [file 13750_2024_326_MOESM5_ESM.docx]

**Table 5.1.** Critical appraisal criteria

| **Factor** | **Low risk of bias** | **Moderate risk of bias** | **High risk of bias** |
| --- | --- | --- | --- |
| Study design | Experimental studies (treatment vs control) | Experimental studies (including concurrent controls) | Quasi – experimental studies, case studies and observational studies |
| Objectives/hypothesis | Clear relation between objectives and methodology | The relation between objectives and methodology are not very clear | No relation between objectives and methodology |
| Sampling | Sampling method is suitable for gathering data on the population of interest | The method used for sampling is appropriate for data gathering on population of interest | Sampling method is not suitable for the population of interest |
|  | Replicates are proper (interventions are replicated) | Replicates are proper |  |
|  | Intervention and comparator areas are well-matched (soil conditions are the same) | Intervention and comparator areas are well-matched (soil conditions do not differ profoundly) |  |
|  | Confounding factors not present (both intervention and comparator are treated equally) | Confounding factors not present (both intervention and comparator are treated equally) |  |
| Consideration of heterogeneity/effect modifiers | Precisely determined/considered effect modifiers | Effect modifiers are partially considered | Effect modifiers not considered or determined |
| Statistical analysis and data availability | Clear description of statistical analysis and results | Statistical analysis and results are clear enough | No appropriate statistical analysis and results |
