# Supplementary material for: Biochar improves the nutrient cycle in sandy-textured soils and increases crop yield: a systematic review
Source: Environ Evid. 2024 Feb 22;13:3. doi: 10.1186/s13750-024-00326-5 (PMC11376106; doi:10.1186/s13750-024-00326-5)
Supplement: Supplementary file 7 — Additional file 7. Updated data coding table for data extraction. [file 13750_2024_326_MOESM7_ESM.docx]

**Table 7.1.** Data coding. It is updated during the data extraction processes

| **#** | **Category** | **Variable** | **Description** |
| --- | --- | --- | --- |
| 1 | Study ID | First Author | Mainly last name of the first author |
|  |  | Publication year | Year the article is published |
|  |  | Numbering | Code for each data point in the study |
|  |  | EsID | ID number for each effect size |
|  |  | Publication title | The title of each included publication |
| 2 | Type of paper | Peer-reviewed | If the paper is published in a peer-reviewed journal |
|  |  | Gray literature | If it is a publication in a website, or a dissertation/thesis, or conference summaries |
| 3 | Study location | Study area | The area where the study has been conducted |
|  |  | Longitude and latitude | The longitude and the latitude of study area |
|  |  | Country | The country where the study area is located |
|  |  | Type of climate | Classification of climate of the country |
|  |  | Average precipitation | Expressed in mm: Average precipitation in study area |
|  |  | Annual temperature | Expressed in Celsius scale: Annual temperature in study area or in study design |
| 4 | Experimental condition | Condition | Listing if it is randomized replicates or different |
|  |  | Experimental design | If it is based on open field, greenhouse, or lab |
|  |  | Design characteristics | Plot area, number of plots, or pots (if it is greenhouse or lab experiment) |
|  |  | Experimental setup | Control (without amendment, or with fertilizer or with manure or with compost), treatment (with biochar, or fertilizer + biochar, or manure + biochar, or compost + biochar) |
|  |  | Duration of experiment | Expressed in days |
|  |  | Biochar application rate | Expressed in t/ha |
|  |  | Fertilizer application rate | Expressed in kg/ha |
|  |  | Manure application rate | Expressed in kg/ha |
|  |  | Compost application rate | Expressed in kg/ha |
| 5 | Biochar properties | Feedstock used | The feedstocks used for pyrolysis will be grouped as “woody biomass”, “manure \| digestate”, and “agricultural residue” |
|  |  | Pyrolysis temperature | Expressed in the Celsius scale. Pyrolysis temperature is grouped into low (≤ 400 °C), medium (400 – 600 °C), and high (≥ 600°C) |
|  |  | Carbon rate | Expressed in g per kg (g/kg) and mass percentage |
|  |  | Biochar pH | pH level of biochar |
| 6 | Soil status | Type of soil | Type of sandy soil used for the experiment |
|  |  | Soil pH | pH level of experimental soil |
|  |  | Soil bulk density | Bulk density of experimental soil |
|  |  | Soil sample depth | The minimum and maximum soil depth at which biochar is applied; expressed in cm |
|  |  | Soil treatment before biochar | NPK fertilizer, manure, or other treatments |
|  |  | Soil condition | Soil is tilled or irrigated during the experiment |
| 7 | Soil ecosystem properties | Measurement | List of ESS measured in the study |
|  |  | Measurement unit | Unit used to measure ESS |
| 8 | Changes in ecosystem services | Control (without amendment, or with manure, or with fertilizer) | Means, SD, SE, OR P-value |
|  |  | Treatment (with biochar only, or with manure + biochar, or fertilizer + biochar) | Means, SD, SE, OR P-value |
