# Supplementary material for: Biochar improves the nutrient cycle in sandy-textured soils and increases crop yield: a systematic review
Source: Environ Evid. 2024 Feb 22;13:3. doi: 10.1186/s13750-024-00326-5 (PMC11376106; doi:10.1186/s13750-024-00326-5)
Supplement: Supplementary file 8 — Additional file 8. Statistical analysis of variation in the results of different measurement methods of SPPs. [file 13750_2024_326_MOESM8_ESM.docx]

| **Table 8.1.** Different measurement methods of soil phosphorus | |
| --- | --- |
| **P Extraction method** | **Description** |
| Mehlich 3 extraction | **widely used extractant for evaluating plant available phosphorus (P) in soils** and may be quantified using colorimetric or inductively coupled plasma (ICP) spectroscopic methods. |
| Olsen P extraction | is the official method used in Southern Brazil to evaluate soil available P. In this method, **labile P is extracted by acid dissolution**, which preferentially attacks P pools associated with calcium compounds and, to a less extent with aluminum and iron compounds. |
| Biologically based P (BBP) method | I think this method is used to evaluate the P fraction in the soil. But Is it the same as Olsen P method??? |
| Acetic acid extraction | The acetic acid phosphorus extraction method which has the best correlation with the **Olsen method.** Simplified acetic acid method was the best simple P extraction method in the field level test |
| Bray 1 method (can be grouped as Bay method) | **A 1 gram scoop of air-dried soil and 10 milliliters of extractant are shaken for 5 minutes**. The amount of phosphorus extracted is determined by measuring the intensity of the blue color developed in the filtrate when treated with molybadate-ascorbic acid reagent. |
| Bray and Kurtz method | The small amounts of soil P extracted by water (mainly P in dissolved forms) and difficulties related to chemical analysis limit the use of water as an extractant. Bray and Kurtz (1945) **suggested a combination of HCl and NH_4_F to remove easily acid soluble P forms, largely Al- and Fe-phosphates**. |
| Bray II extraction | **The P-Bray 1 Method** removes a fraction of the “adsorbed” phosphorus (Al-P, Fe-P, Mn-P and Ca-P but less efficient) while **the P-Bray 2 Method** is best suited to acid soils where rock phosphate has been the primary P fertilizer source and/or the major portion of P exists in the soil in various forms of calcium phosphate |
| Mehlich 3 extraction – Bray 1 can be similar | **The Mehlich 3 method** is a weak acid soil extraction procedure that has the advantage of being applicable for a number of elements. The extract is composed of 0.2 M glacial acetic acid, 0.25 M ammonium nitrate, 0.015 M ammonium fluoride, 0.013 M nitric acid, and 0.001 M ethylene diamine tetraacetic acid (EDTA). |
| Molybdenum blue colorimetric method 🡪 **not an extraction method (many of them used Sodium bicarbonate method)** | This method is based on the formation of phosphomolybdate complex with the added molybdate followed by the reduction of the complex with hydrazine hydrate in aqueous sulphuric acid medium. |
| Sodium bicarbonate method | Extraction of Phosphate from soil by 0.5 M sodium bicarbonate at pH 8.5 |
| 0.05-mol L−1 HCl and 0.025-mol L−1 (1/2 H2SO4) solutions and measured by spectrophotometer | **Acid extraction (strong)** |

In order to check if there is a significant difference between the extraction methods, we ran regression analysis where we could see how different extraction methods would effect on the values. To check if there are any statistically significant differences between the means of P extraction methods, we applied the anova. This method also helps understand if the means of LnRR (response variable) differ significantly across the factor (P -extraction methods)

Here are the linear regression results:


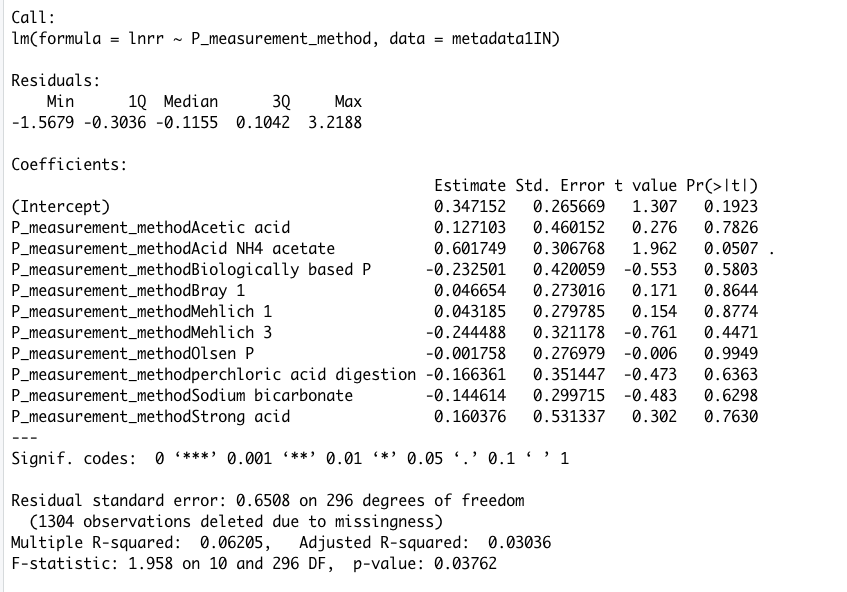


Anova results:


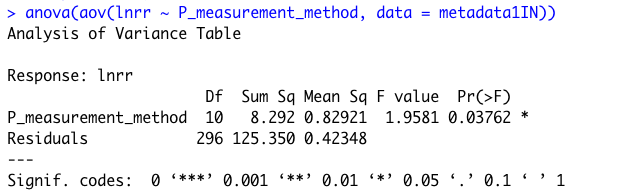


We also ran emmeans function to estimate marginal means for linear regression analysis


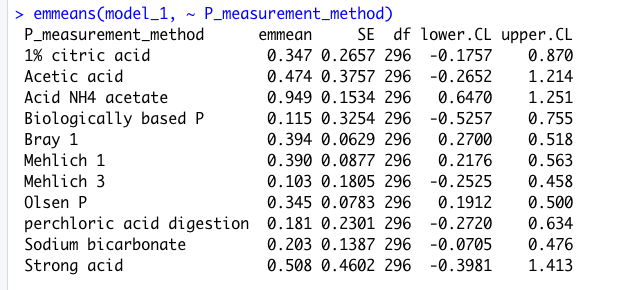


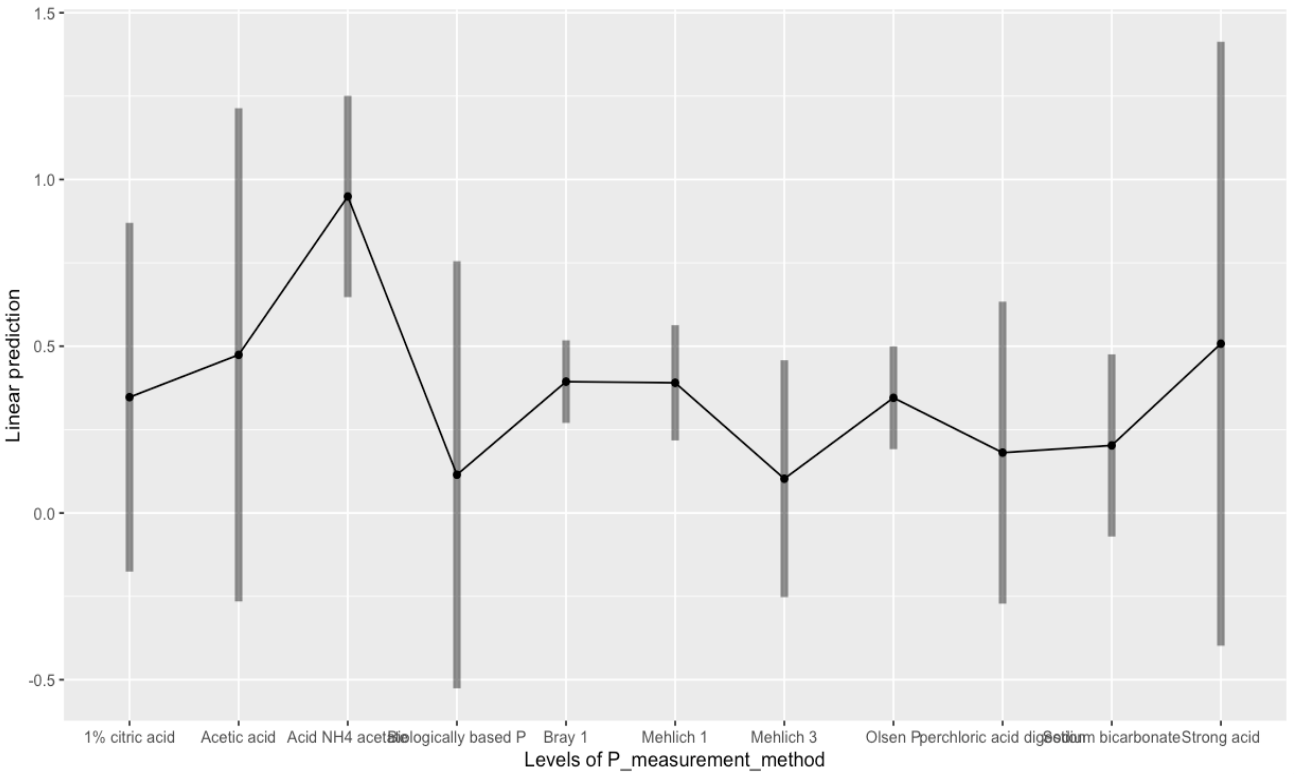


From this graph we can see that the significant difference between groups are negligible, while only one of the extraction methods which is Acid Ammonium acetate slightly different. We also checked this applying Tukeys Post hoc method and the results of post hoc approach was the same. Based on that, all the P extraction methods merged as one because there is no high significantly difference in different method results
