# Supplementary material for: Biochar improves the nutrient cycle in sandy-textured soils and increases crop yield: a systematic review
Source: Environ Evid. 2024 Feb 22;13:3. doi: 10.1186/s13750-024-00326-5 (PMC11376106; doi:10.1186/s13750-024-00326-5)
Supplement: Supplementary file 10 — Additional file 10. Descriptive statistics. Contains further descriptions of data for narrative synthesis. [file 13750_2024_326_MOESM10_ESM.docx]

**Descriptive statistics**


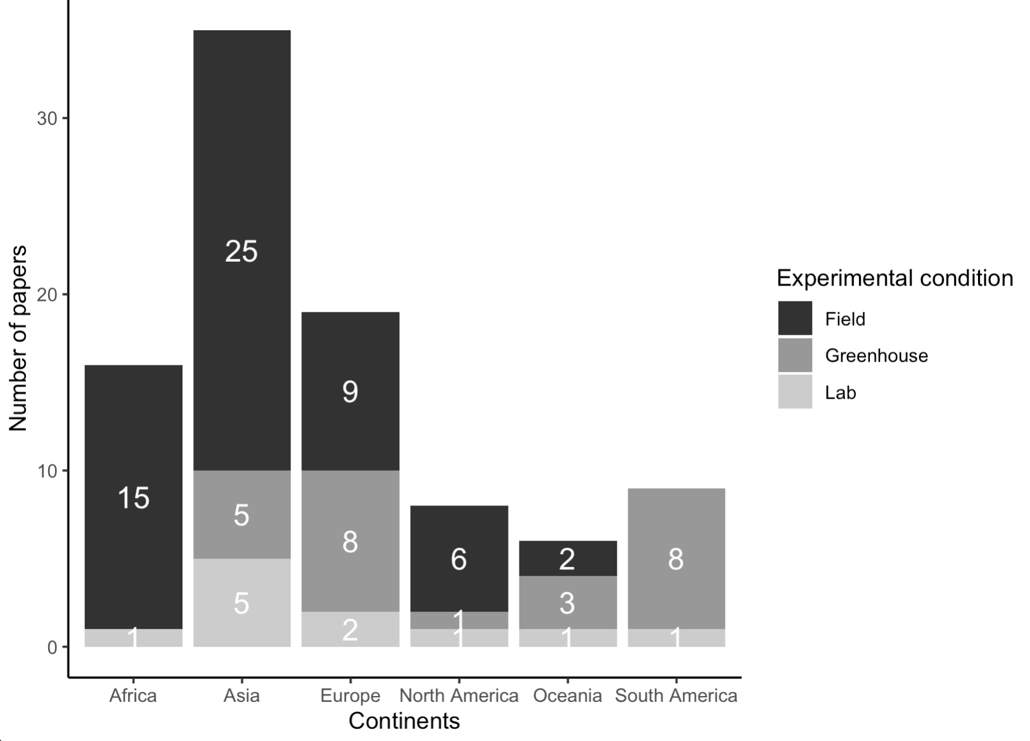


**Figure 10.1.** Studies with different experimental conditions per continent


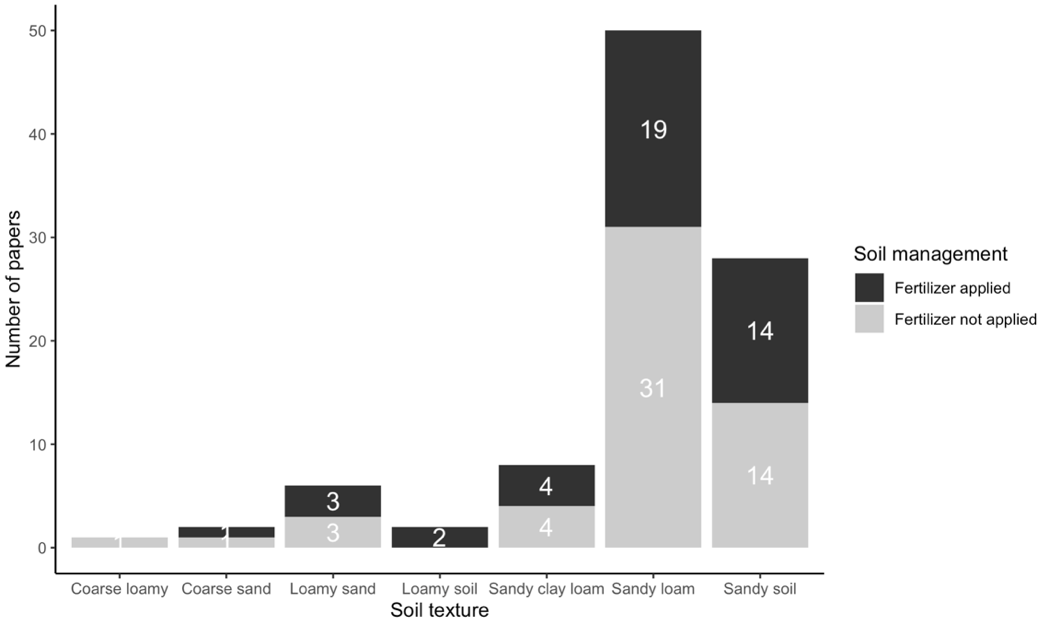


**Figure 10.2.** Studies with different soil managements per soil type


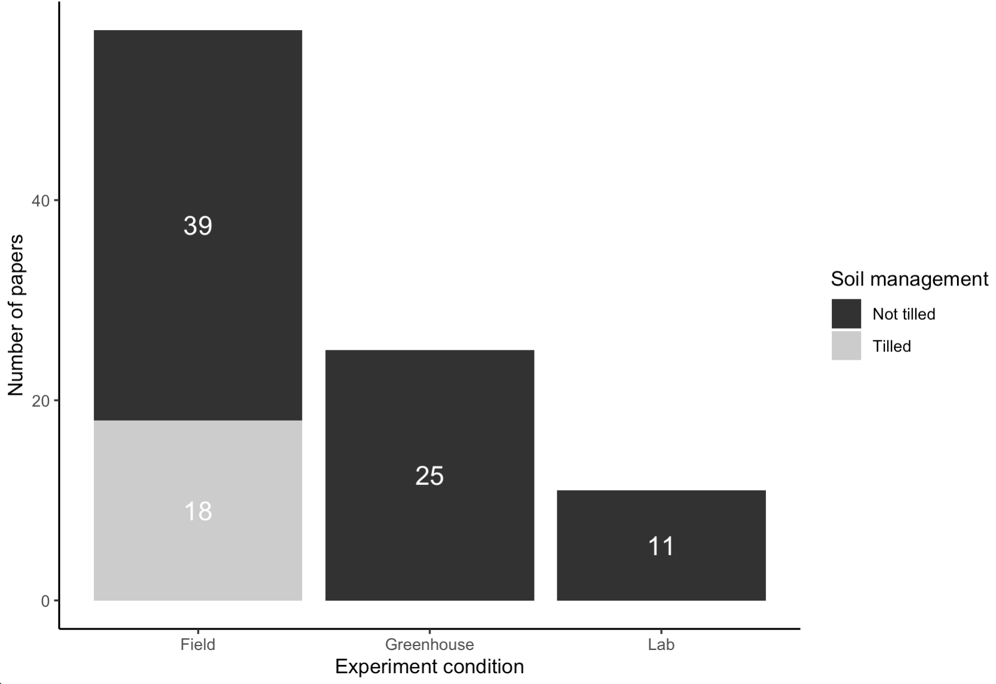


**Figure 10.3.** Studies with different soil managements per study design


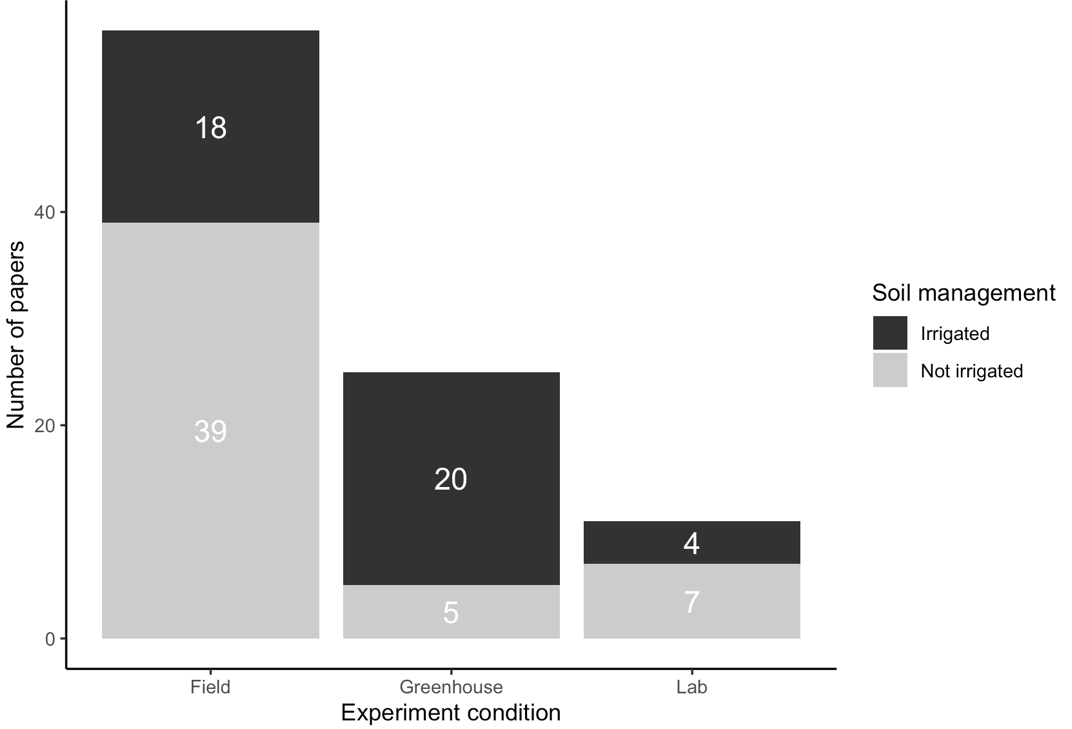


**Figure 10.4.** Studies with different soil managements per study design


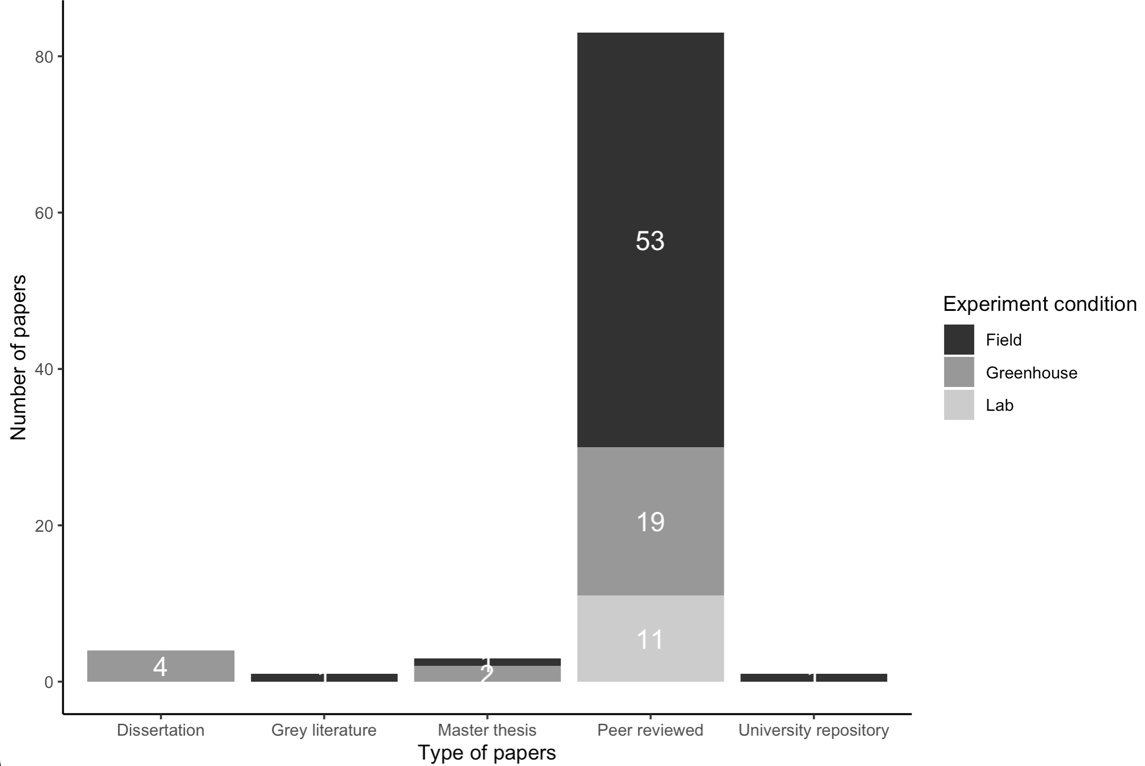


**Figure 10.5.** Studies with different soil managements per study design

**Table 10.1.** Number of observations per feedstock type in different pyrolysis groups

| Feedstock type | Pyrolysis group | Unique studies | Number of observations |
| --- | --- | --- | --- |
| Agricultural residue | High | 7 | 67 |
| Agricultural residue | Low | 22 | 414 |
| Agricultural residue | Medium | 25 | 326 |
| Manure\|digestate | High | 3 | 9 |
| Manure\|digestate | Low | 4 | 32 |
| Manure\|digestate | Medium | 11 | 135 |
| Woody | High | 9 | 97 |
| Woody | Low | 11 | 103 |
| Woody | Medium | 26 | 362 |

**Table 10.2.** Number of observations per tillage application in different experimental conditions

| Tillage application | Experiment condition | Unique studies | Number of observations |
| --- | --- | --- | --- |
| Not tilled | Field | 39 | 551 |
| Not tilled | Greenhouse | 25 | 496 |
| Not tilled | Lab | 11 | 211 |
| Tilled | Field | 18 | 341 |

**Table 10.3.** Number of observations per soil management in different sandy-textured soils

| NPK before biochar | Soil type | Unique studies | Number of observations |
| --- | --- | --- | --- |
| Fertilizer applied | Coarse sand | 1 | 5 |
| Fertilizer applied | Loamy sand | 3 | 75 |
| Fertilizer applied | Loamy soil | 2 | 28 |
| Fertilizer applied | Sandy clay loam | 4 | 43 |
| Fertilizer applied | Sandy loam | 19 | 198 |
| Fertilizer applied | Sandy soil | 14 | 240 |
| Fertilizer not applied | Coarse loamy | 1 | 9 |
| Fertilizer not applied | Coarse sand | 1 | 4 |
| Fertilizer not applied | Loamy sand | 3 | 81 |
| Fertilizer not applied | Sandy clay loam | 4 | 122 |
| Fertilizer not applied | Sandy loam | 31 | 536 |
| Fertilizer not applied | Sandy soil | 14 | 258 |

**Table 10.4.** Number of observations per experiment duration in different experimental conditions

| Experiment duration | Experiment condition | Unique studies | Number of observations |
| --- | --- | --- | --- |
| 0-60 | Field | 1 | 4 |
| 0-60 | Greenhouse | 9 | 202 |
| 0-60 | Lab | 4 | 40 |
| 60-160 | Field | 10 | 190 |
| 60-160 | Greenhouse | 11 | 232 |
| 60-160 | Lab | 7 | 77 |
| 160-402 | Field | 17 | 236 |
| 160-402 | Greenhouse | 4 | 54 |
| 160-402 | Lab | 2 | 94 |
| 402-730 | Field | 19 | 310 |
| 402-730 | Greenhouse | 1 | 8 |
| 730-2200 | Field | 9 | 152 |

| Treatment | Control | Unique studies | Number of observations |
| --- | --- | --- | --- |
| Biochar alone | Control no amendment | 79 | 1020 |
| Biochar with compost | Control with compost | 6 | 33 |
| Biochar with manure | Control with manure | 11 | 127 |
| Biochar with fertilizer | Control with fertilizer | 30 | 401 |
| Biochar with fertilizer & compost | Control with fertilizer & compost | 1 | 6 |
| Biochar with fertilizer & manure | Control with fertilizer & manure | 2 | 12 |

**Table 10.5.** Number of observations per control and treatment
