# Supplementary material for: Biochar improves the nutrient cycle in sandy-textured soils and increases crop yield: a systematic review
Source: Environ Evid. 2024 Feb 22;13:3. doi: 10.1186/s13750-024-00326-5 (PMC11376106; doi:10.1186/s13750-024-00326-5)
Supplement: Supplementary file 11 — Additional file 11. Sensitivity analyses. The effect of outlier removal and estimated SD from P value removal on the total outcome. [file 13750_2024_326_MOESM11_ESM.docx]

**Outlier detection and removal**

Before starting the analysis, we explored the meta-analytical dataset. When we drew a funnel plot for each SEP, we witnessed some existing outliers in the dataset due to higher values in standard error (SE). We tried to create a criterion to remove those outliers as they affected the final results. A criterion has been developed as follows; the first and third quartiles and the interquartile range (IQR) were found and if the 1/se is below the first quartile or above the third quartile by a factor of 10 times the IQR, these data points were outliers and excluded from the dataset. Results with and without outliers did not differ and we succeeded in eliminating these enormously large values from the dataset.

**Soil total NPK**

| \| 1. 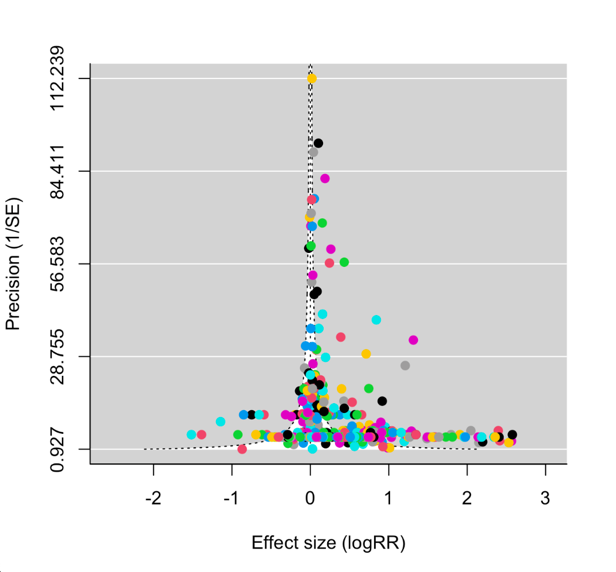 \| 1. 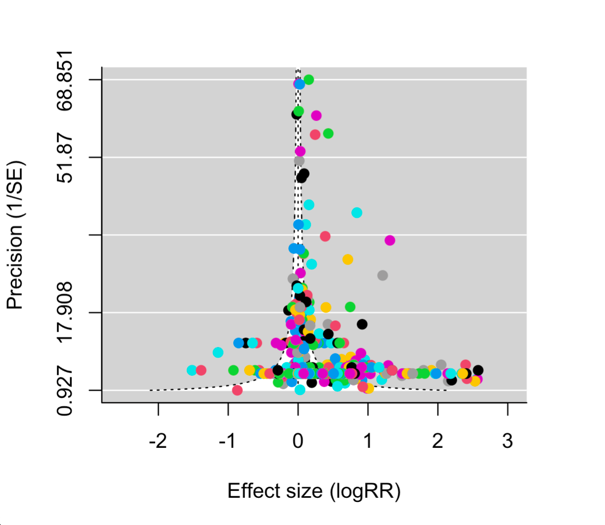 \| \| --- \| --- \| |
| --- | --- | --- |
| **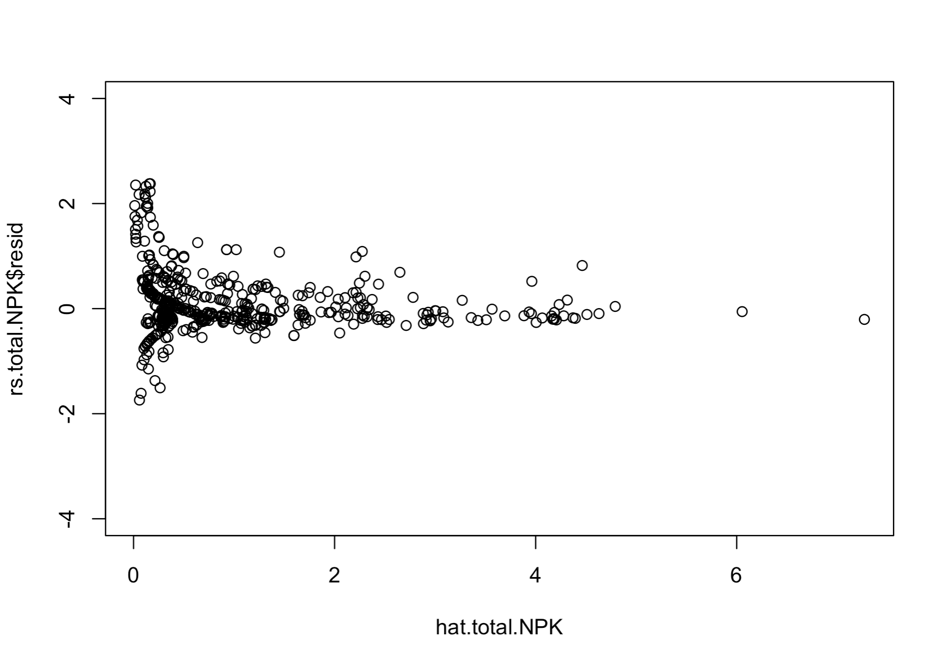**c. |

**Figure 11.1.** Funnel plots with 1/SE for lnRR as measures of uncertainty to visualize outliers in the dataset of soil total NPK. a. Funnel plot before outlier removal was created based on 504 observations; b. Funnel plot after outlier removal was created based on 486 observations; c. results of hat values: an alternative approach to funnel plots.

**Soil mineral nitrogen**

| 1. 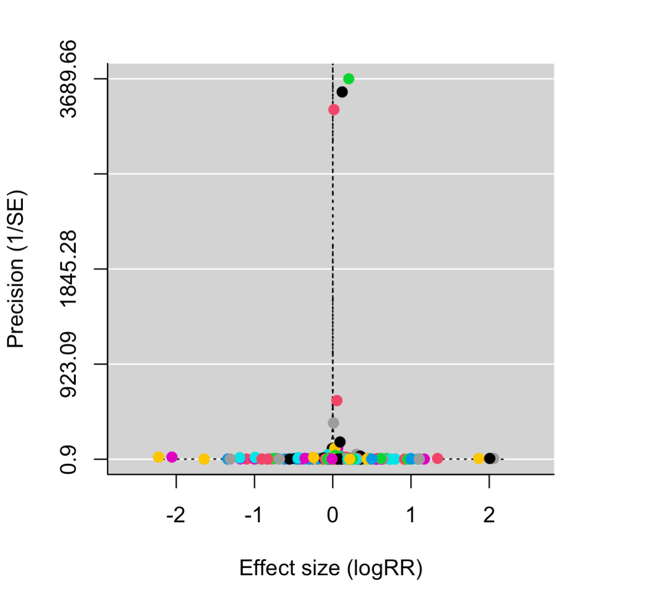 | 1. 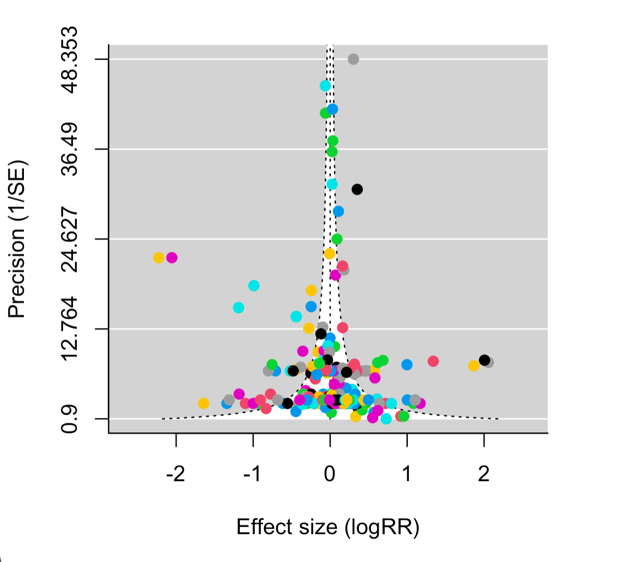 |
| --- | --- |

**Figure 11.2.** Funnel plots with 1/SE for lnRR as measures of uncertainty to visualize outliers in the dataset of soil mineral nitrogen. a. Funnel plot before outlier removal was created based on 247 observations; b. Funnel plot after outlier removal was created based on 236 observations

**Plant nutrient level**

| 1. **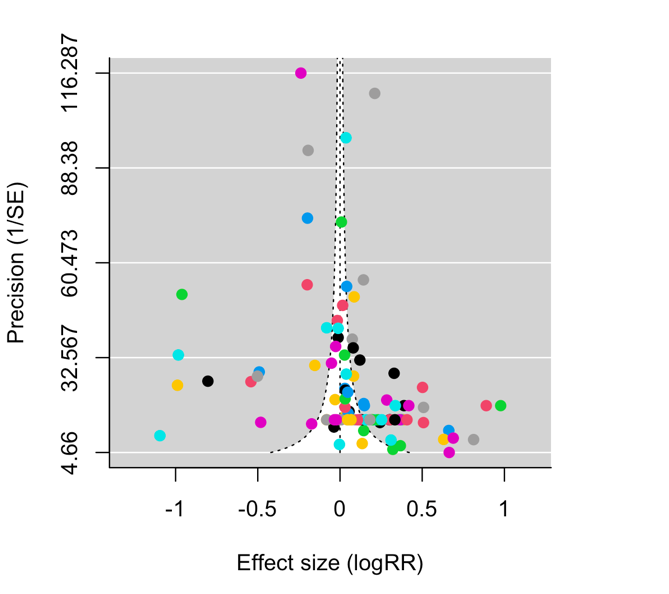** | 1. **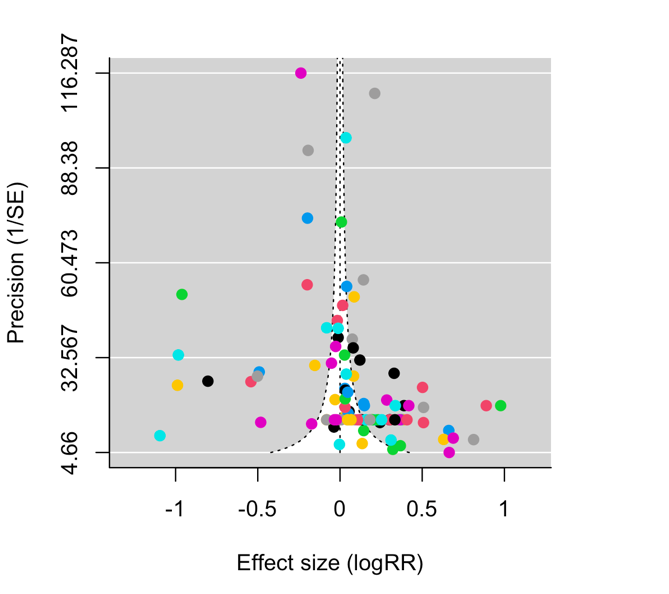** |
| --- | --- |

**Figure 11.3.** Funnel plots with 1/SE for lnRR as measures of uncertainty to visualize outliers in the dataset of plant nutrient level. a. Funnel plot before outlier removal was created based on 106 observations; b. Funnel plot after outlier removal was created based on 100 observations

**N2O emission**

| 1. **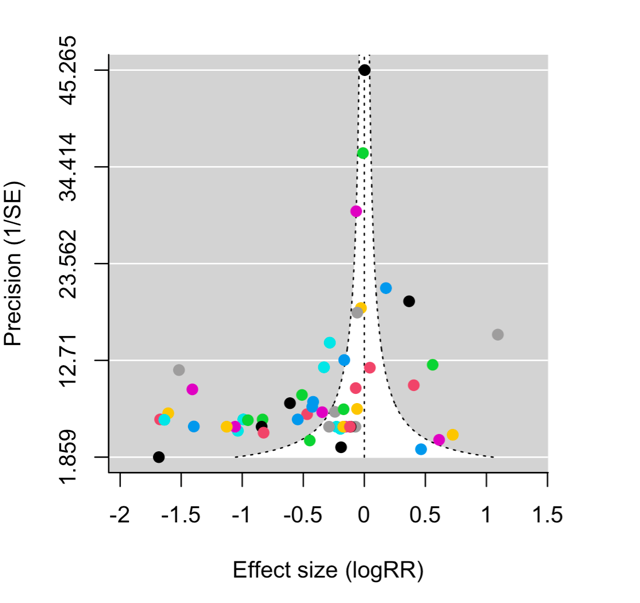** | 1. **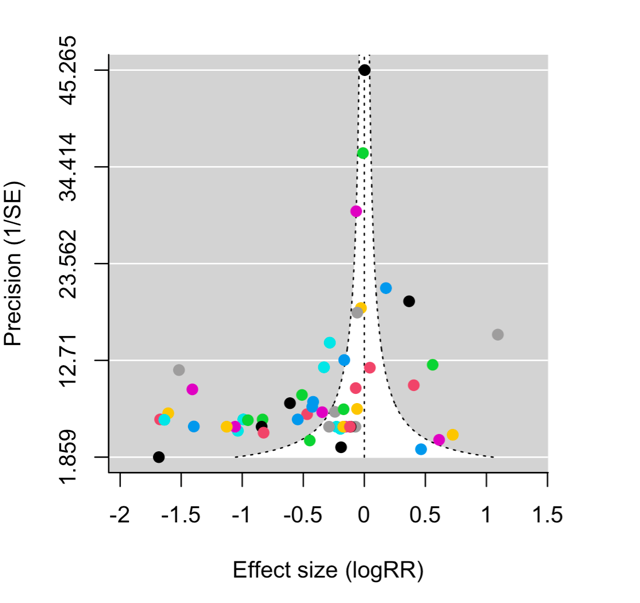** |
| --- | --- |

**Figure 11.4.** Funnel plots with 1/SE for lnRR as measures of uncertainty to visualize outliers in the dataset of N2O emission. a. Funnel plot before outlier removal was created based on 53 observations; b. Funnel plot after outlier removal was created based on 53 observations

**NPK availability**

| 1. **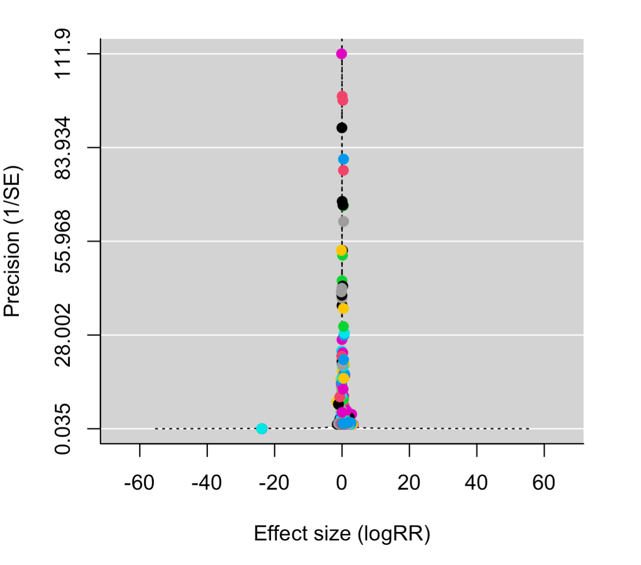** | 1. **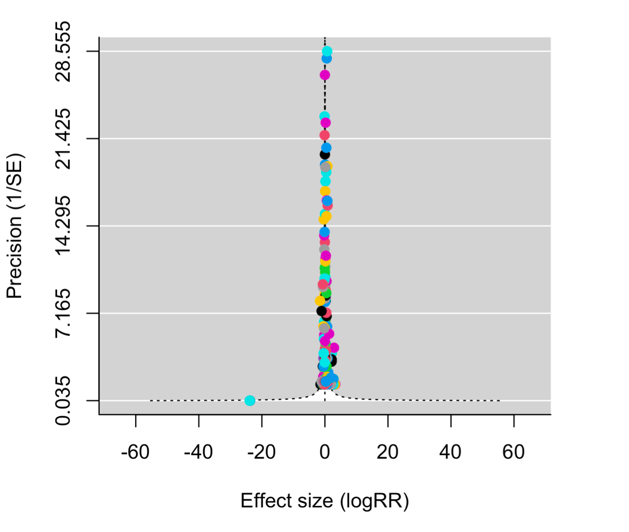** |
| --- | --- |

**Figure 11.5.** Funnel plots with 1/SE for lnRR as measures of uncertainty to visualize outliers in the dataset of NPK availability. a. Funnel plot before outlier removal was created based on 468 observations; b. Funnel plot after outlier removal was created based on 448 observations

**Potential CEC**

| 1. **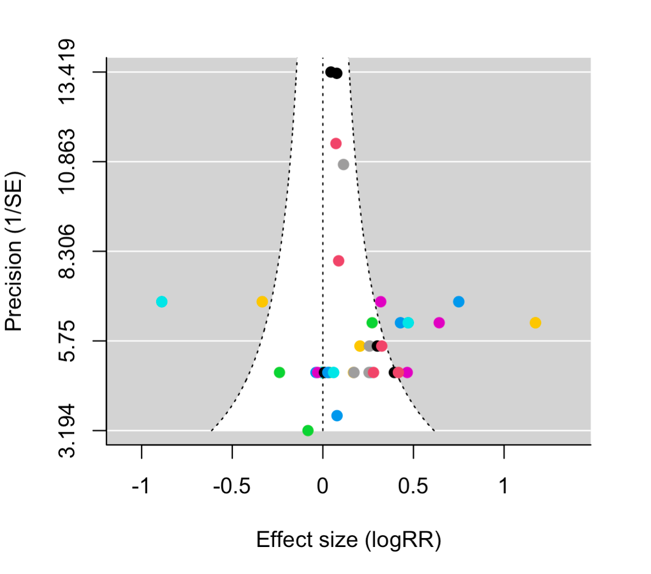** | 1. **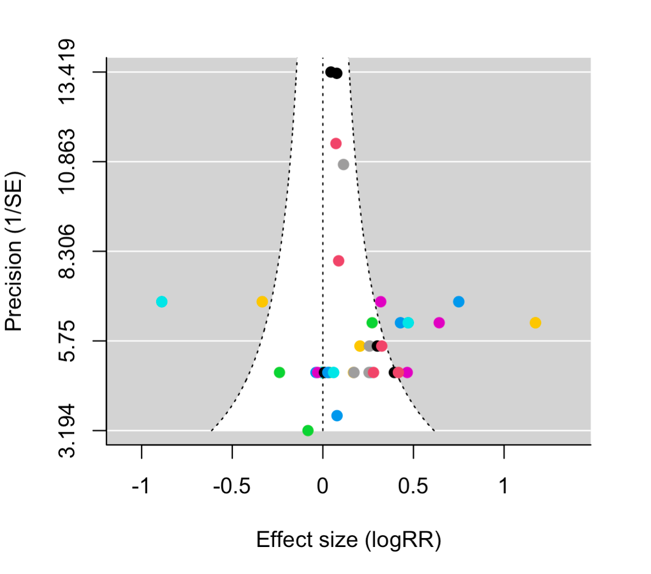** |
| --- | --- |

**Figure 11.6.** Funnel plots with 1/SE for lnRR as measures of uncertainty to visualize outliers in the dataset of potential CEC. a. Funnel plot before outlier removal was created based on 37 observations; b. Funnel plot after outlier removal was created based on 37 observations

**Effective CEC**

| 1. 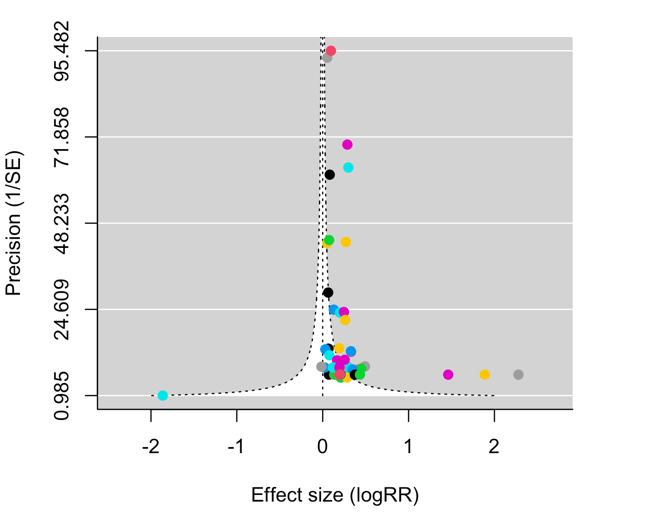 | 1. 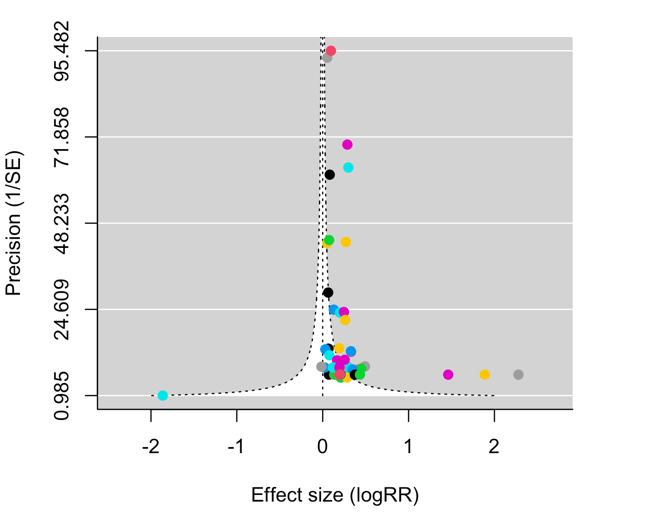 |
| --- | --- |

**Figure 11.7.** Funnel plots with 1/SE for lnRR as measures of uncertainty to visualize outliers in the dataset of effective CEC. a. Funnel plot before outlier removal was created based on 49 observations; b. Funnel plot after outlier removal was created based on 44 observations

**Nutrient use efficiency**

| 1. **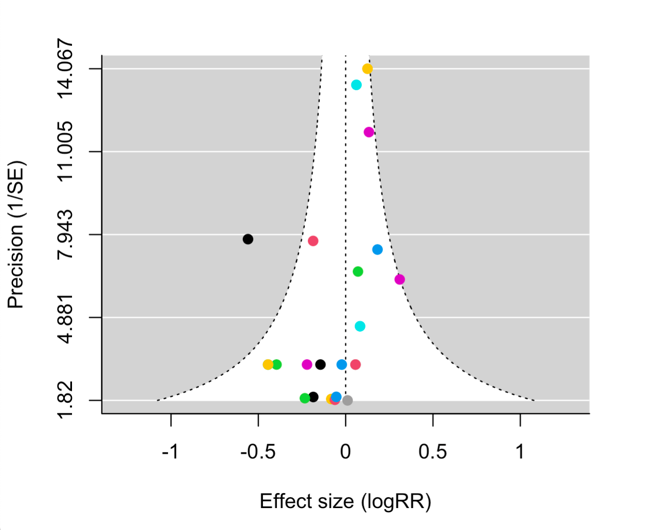** | 1. **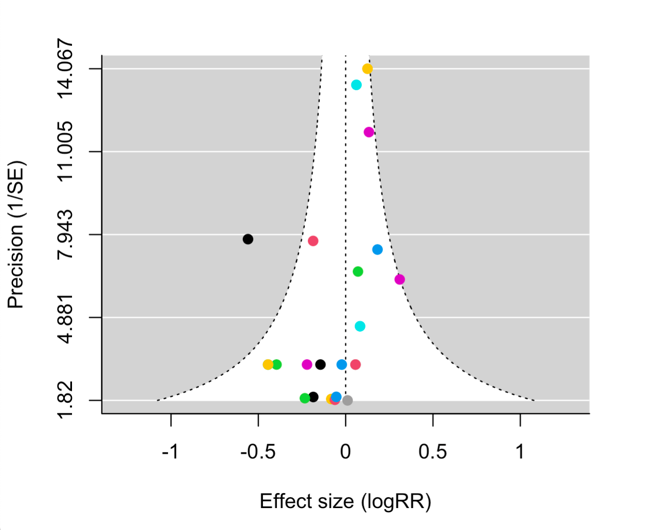** |
| --- | --- |

**Figure 11.8.** Funnel plots with 1/SE for lnRR as measures of uncertainty to visualize outliers in the dataset of nutrient use efficiency. a. Funnel plot before outlier removal was created based on 23 observations; b. Funnel plot after outlier removal was created based on 23 observations

**Mineral N leaching**

| 1. **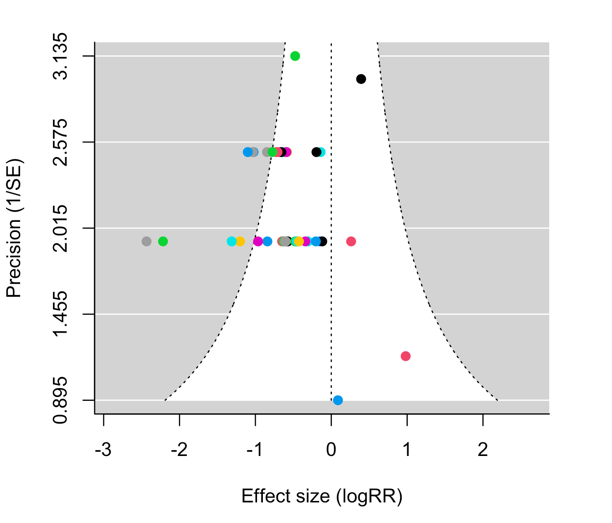** | 1. **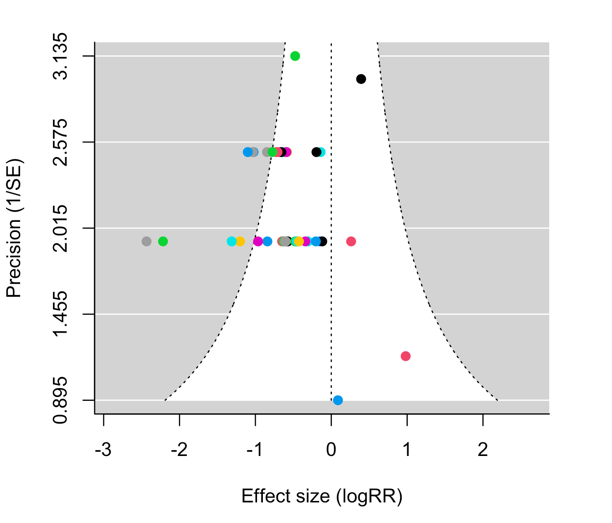** |
| --- | --- |

**Figure 11.9.** Funnel plots with 1/SE for lnRR as measures of uncertainty to visualize outliers in the dataset of mineral N leaching. a. Funnel plot before outlier removal was created based on 40 observations; b. Funnel plot after outlier removal was created based on 40 observations

**Overall effect before and after outlier removal (Missing cases method)**


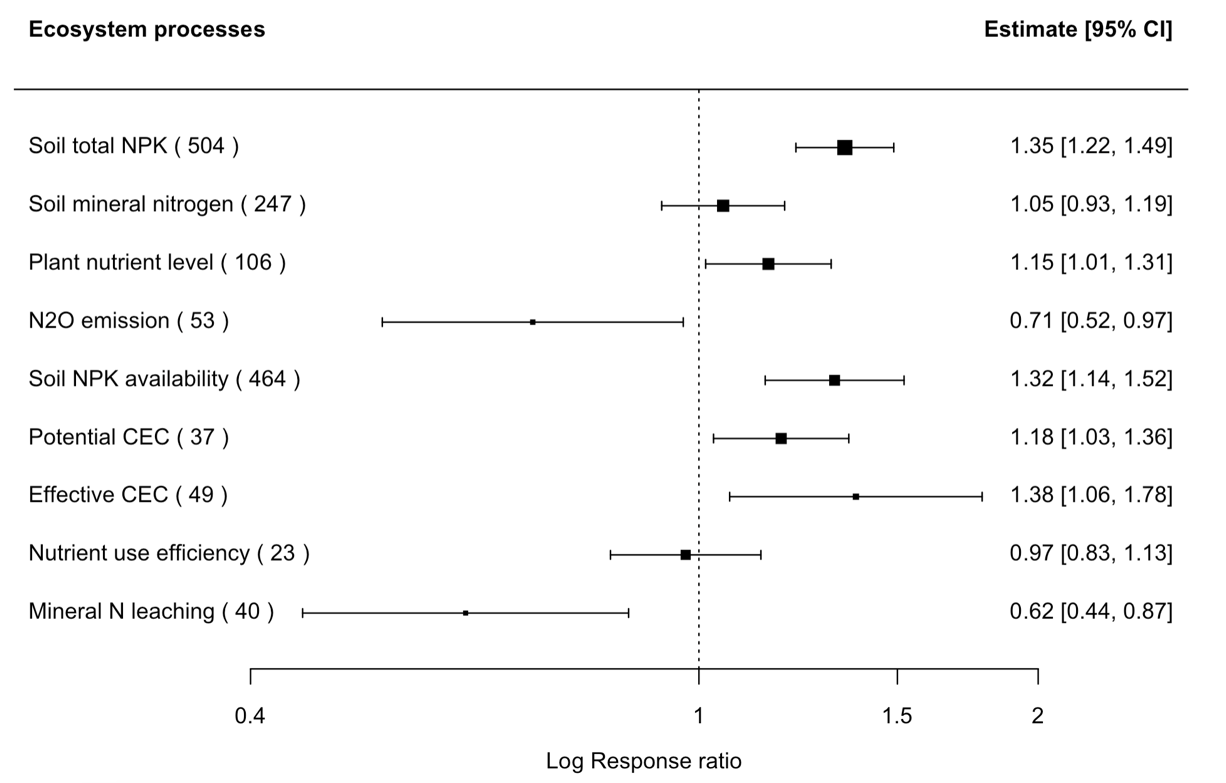
**a.**

**
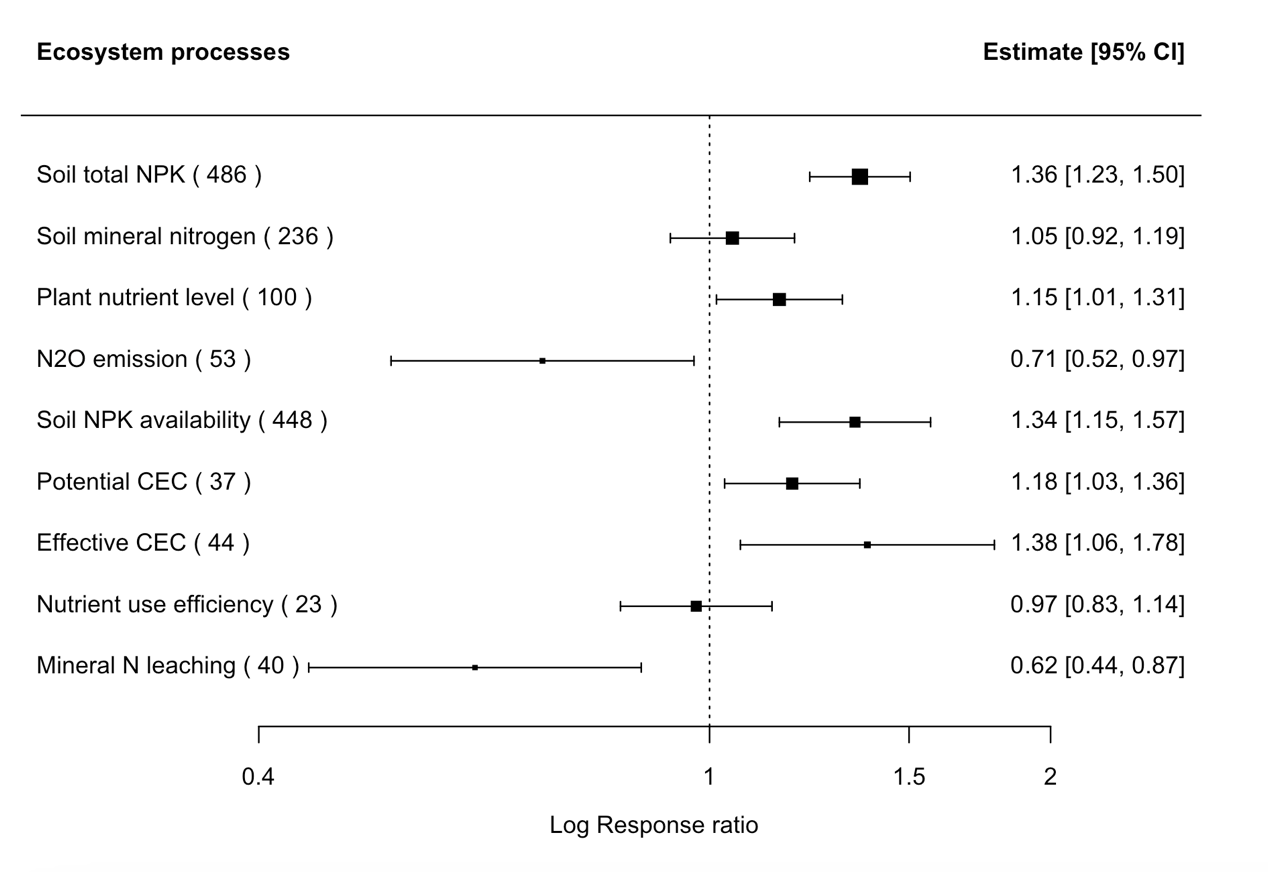
b.**

**Figure 11.10.** The response of SPPs to biochar application; a. the results with outliers. b. the results without outliers. The effect size is the response ratio (Estimate), where the mean value with biochar divided by the mean value without biochar amendment. An effect is significant (P<0.05) if its 95% confidence interval (CI) does not include 1. The reason why confidence intervals are not symmetrical around the effect sizes is because they were back transformed from the log response ratio.

**Overall effect before and after outlier removal (All cases method)**

**
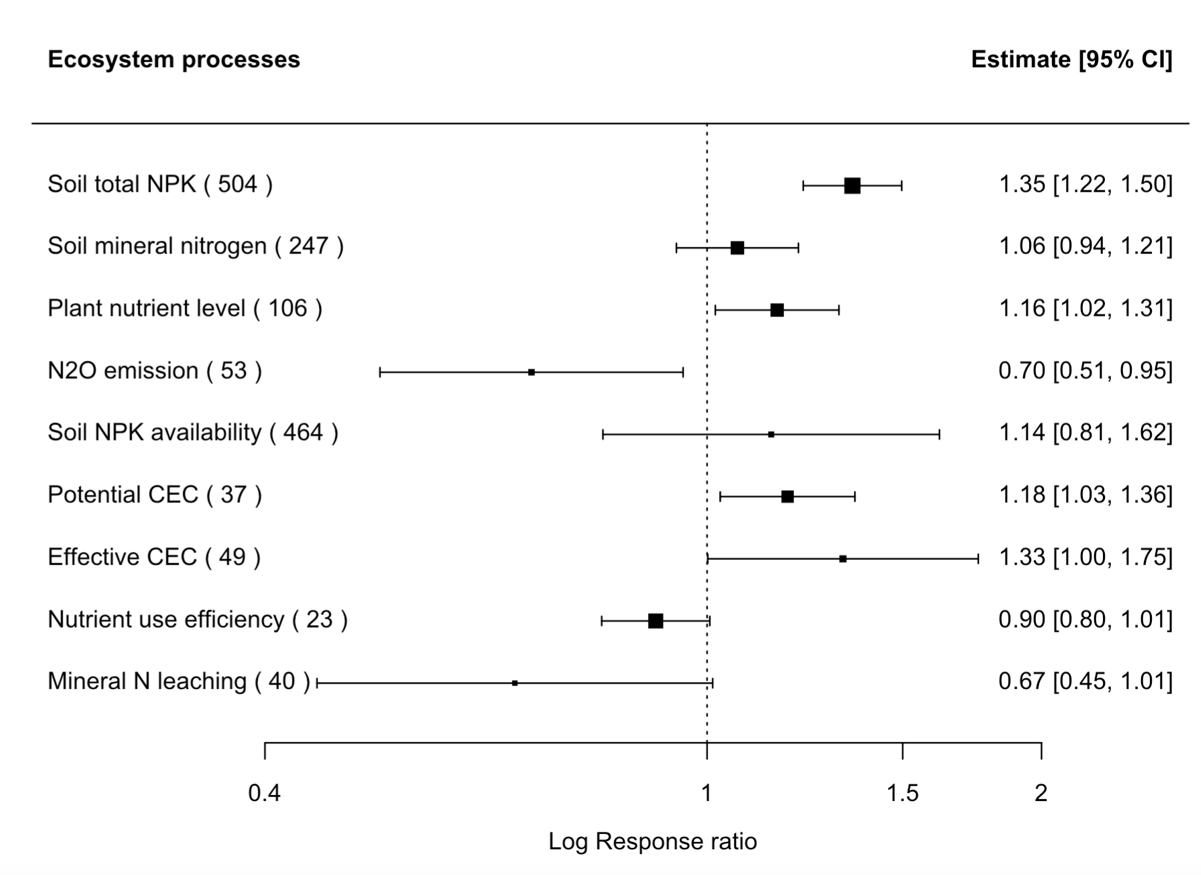
a.**

**
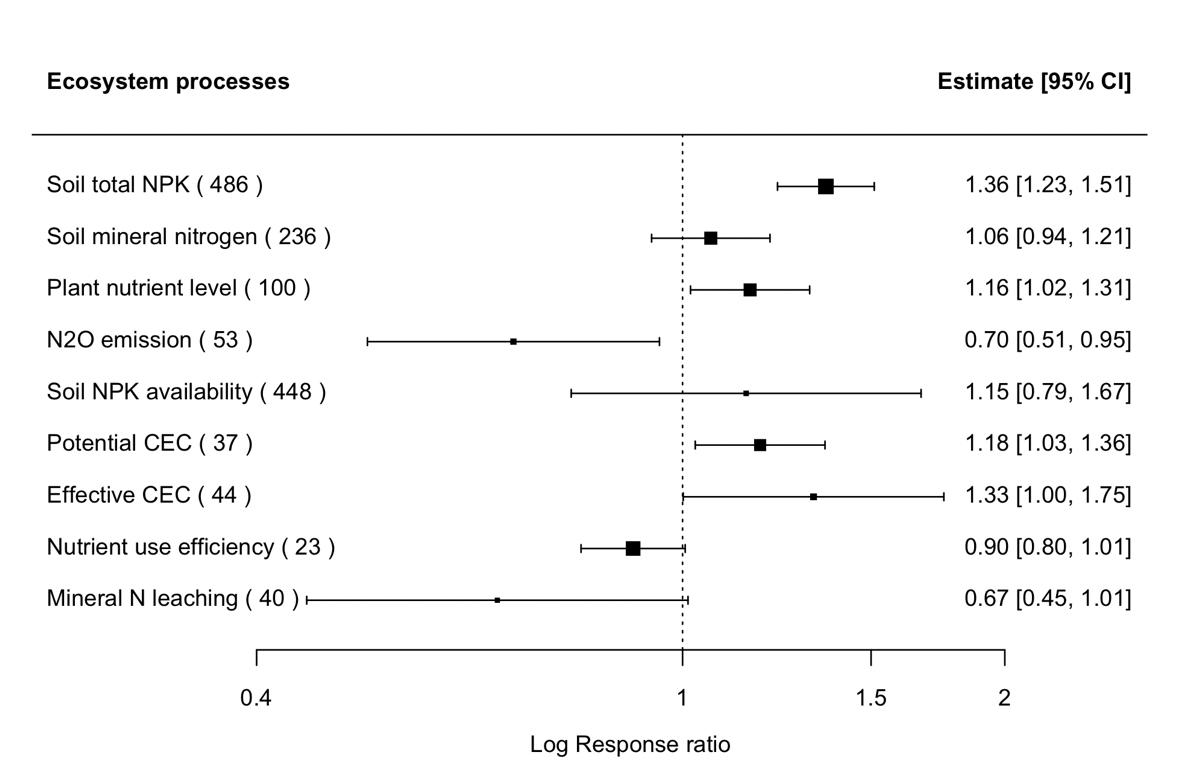
b.**

**Figure 11.11.** The response of SPPs to biochar application; a. the results with outliers. b. the results after outliers removed. The effect size is the response ratio (Estimate), where the mean value with biochar divided by the mean value without biochar amendment. An effect is significant (P<0.05) if its 95% confidence interval (CI) does not include 1. The reason why confidence intervals are not symmetrical around the effect sizes is because they were back transformed from the log response ratio.

**Overall effect before and after outlier removal (multiplicative method)**

1. **
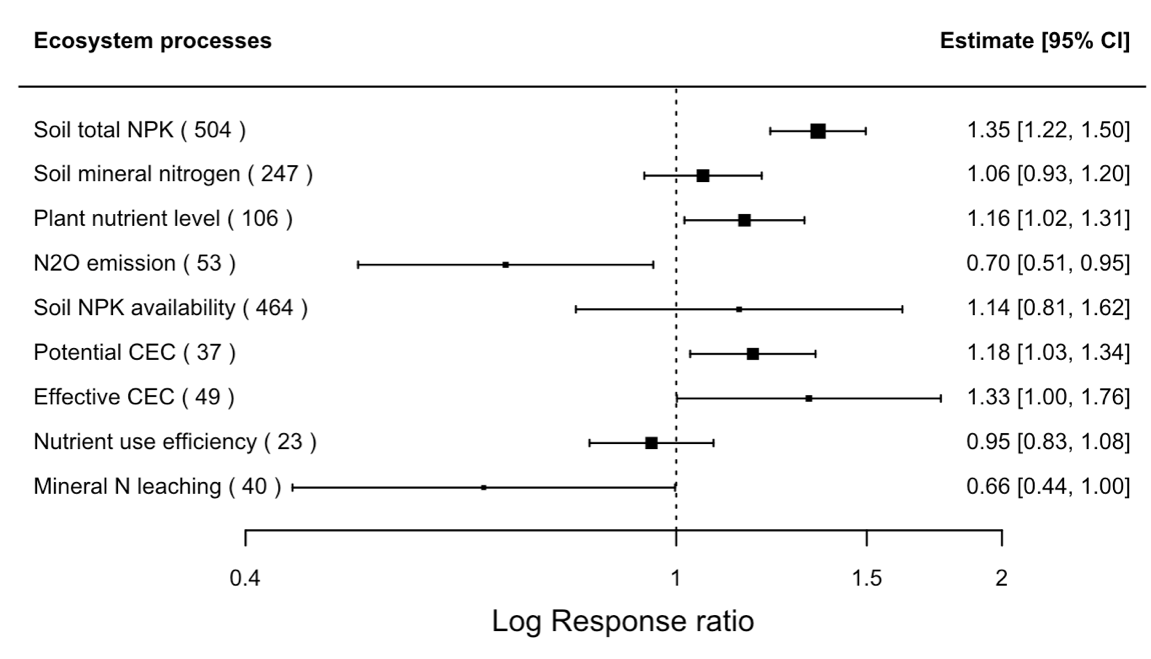
**
2. **
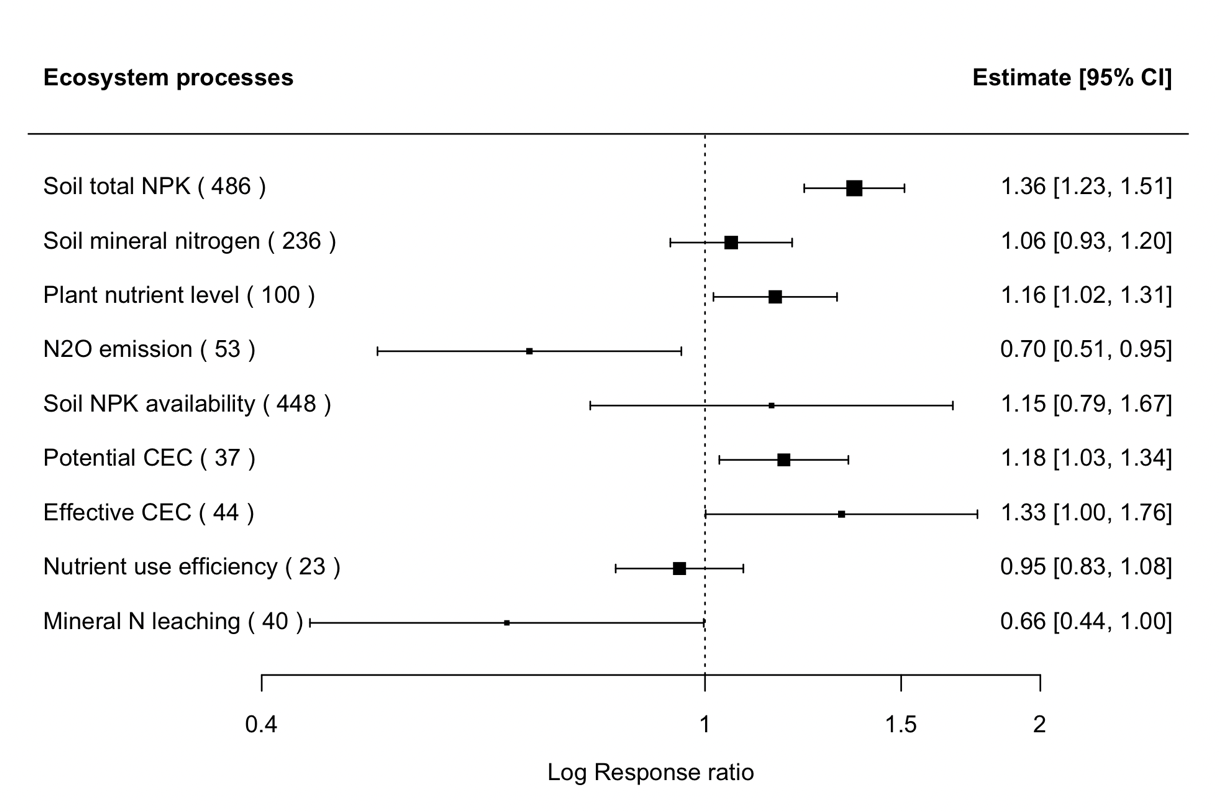
**

**Figure 11.12.** The response of SPPs to biochar application; a. the results with outliers. b. the results after outliers removed. The effect size is the response ratio (Estimate), where the mean value with biochar divided by the mean value without biochar amendment. An effect is significant (P<0.05) if its 95% confidence interval (CI) does not include 1. The reason why confidence intervals are not symmetrical around the effect sizes is because they were back transformed from the log response ratio.

**Overall effect before and after outlier removal (hybrid method)**

1. **
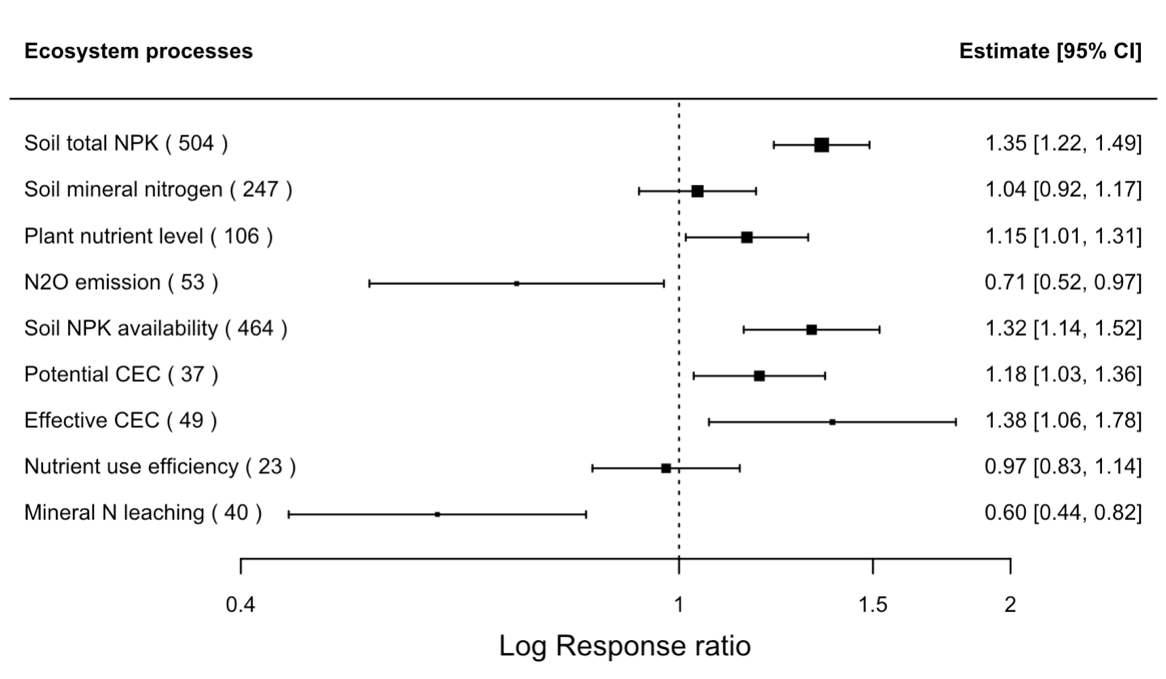
**
2. **
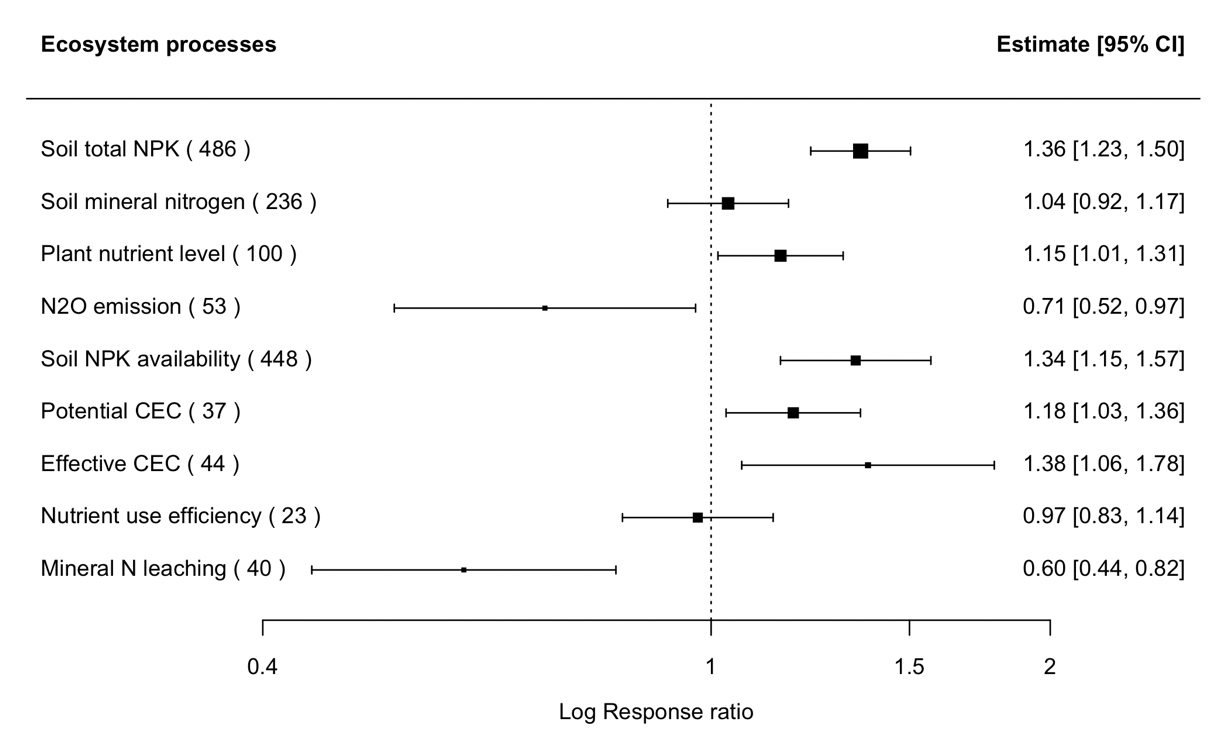
**

**Figure 11.12.** The response of SPPs to biochar application; a. the results with outliers. b. the results after outliers removed. The effect size is the response ratio (Estimate), where the mean value with biochar divided by the mean value without biochar amendment. An effect is significant (P<0.05) if its 95% confidence interval (CI) does not include 1. The reason why confidence intervals are not symmetrical around the effect sizes is because they were back transformed from the log response ratio.

**The effect of assumed Variations from P-values removal on overall effect**

**
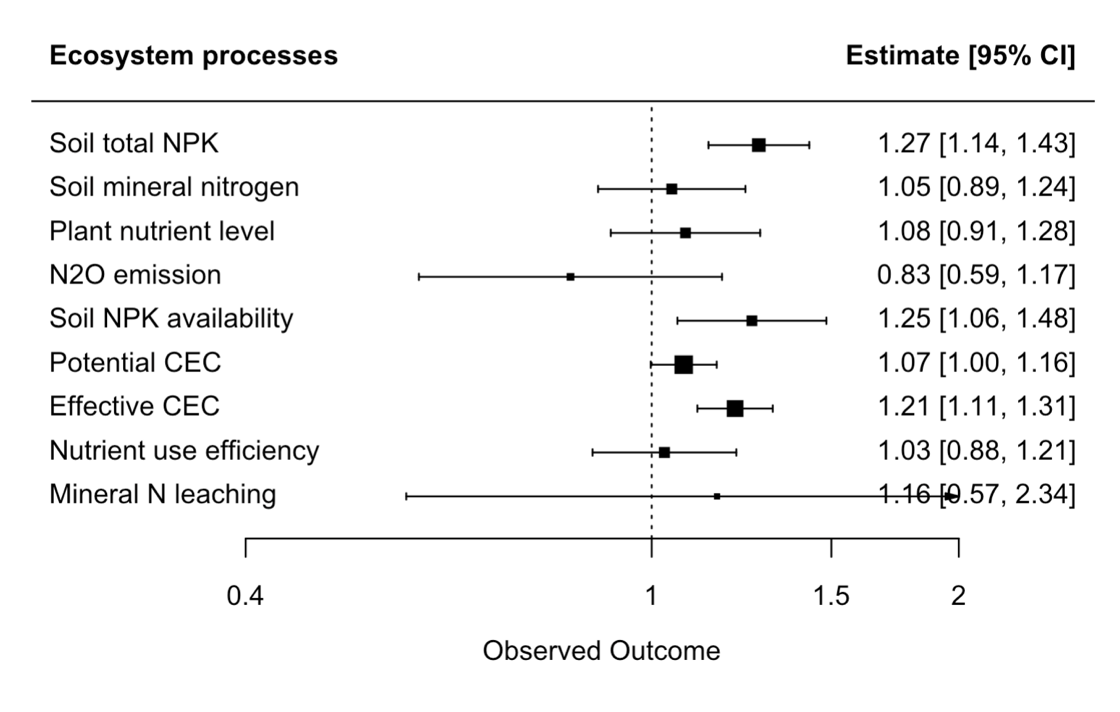
**

**Figure 11.12.** The response of SPPs to biochar application after assumed variations are removed. The effect size is the response ratio (Estimate), where the mean value with biochar divided by the mean value without biochar amendment. An effect is significant (P<0.05) if its 95% confidence interval (CI) does not include 1. The reason why confidence intervals are not symmetrical around the effect sizes is because they were back transformed from the log response ratio.
