# Supplementary material for: Biochar improves the nutrient cycle in sandy-textured soils and increases crop yield: a systematic review
Source: Environ Evid. 2024 Feb 22;13:3. doi: 10.1186/s13750-024-00326-5 (PMC11376106; doi:10.1186/s13750-024-00326-5)
Supplement: Supplementary file 12 — Additional file 12. Meta-regression results. Contains the results of LASSO analyses for each SPP. [file 13750_2024_326_MOESM12_ESM.docx]

**Randomized LASSO result – Soil total NPK**

**
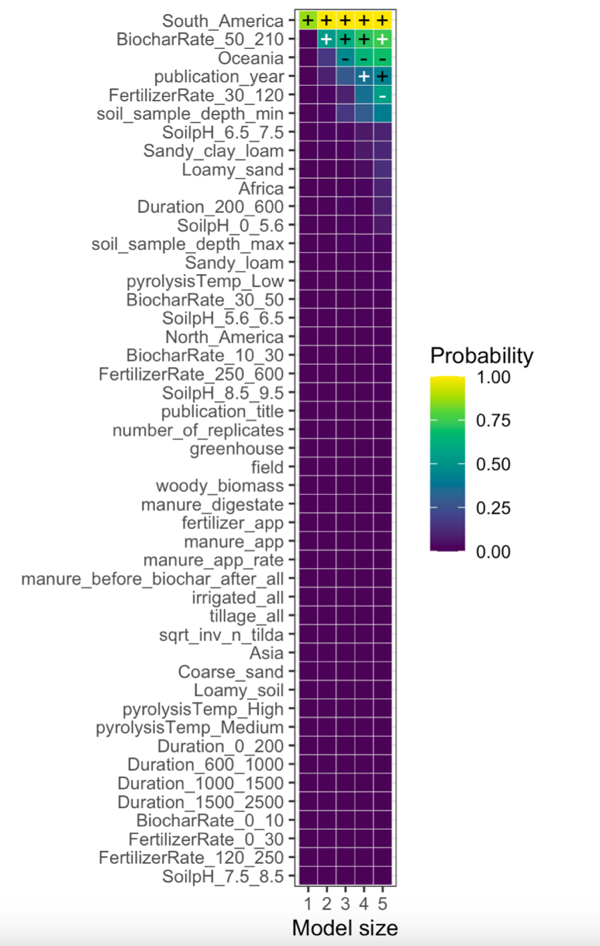
Figure 12.18.** Predictor selection for soil total NPK response by Randomized LASSO

**
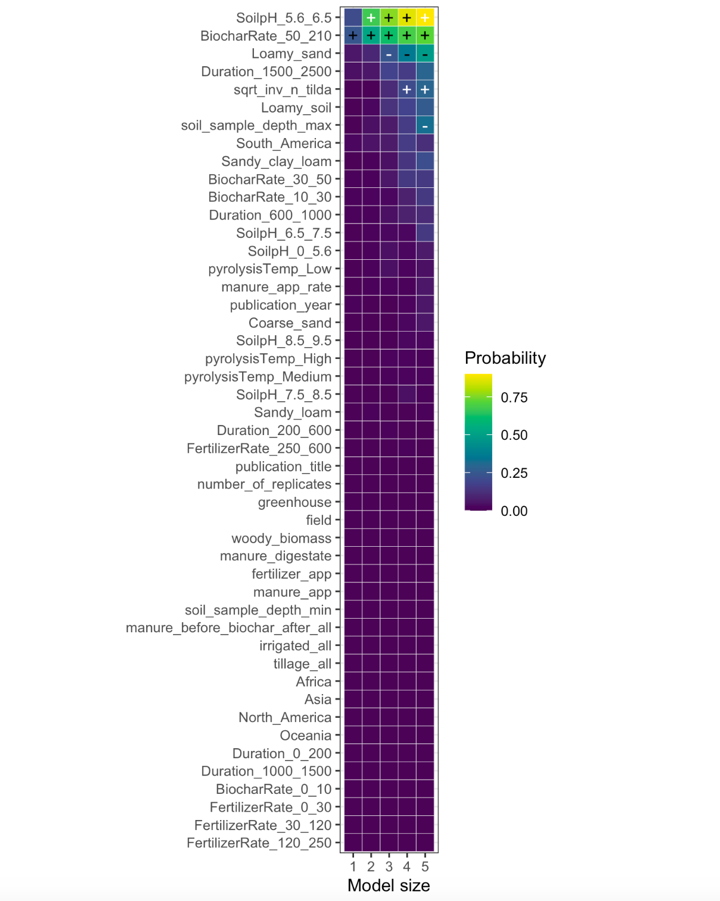
The results of randomized Lasso – soil mineral nitrogen**

**Figure 12.25.** Predictor selection for soil mineral nitrogen by randomized Lass

**
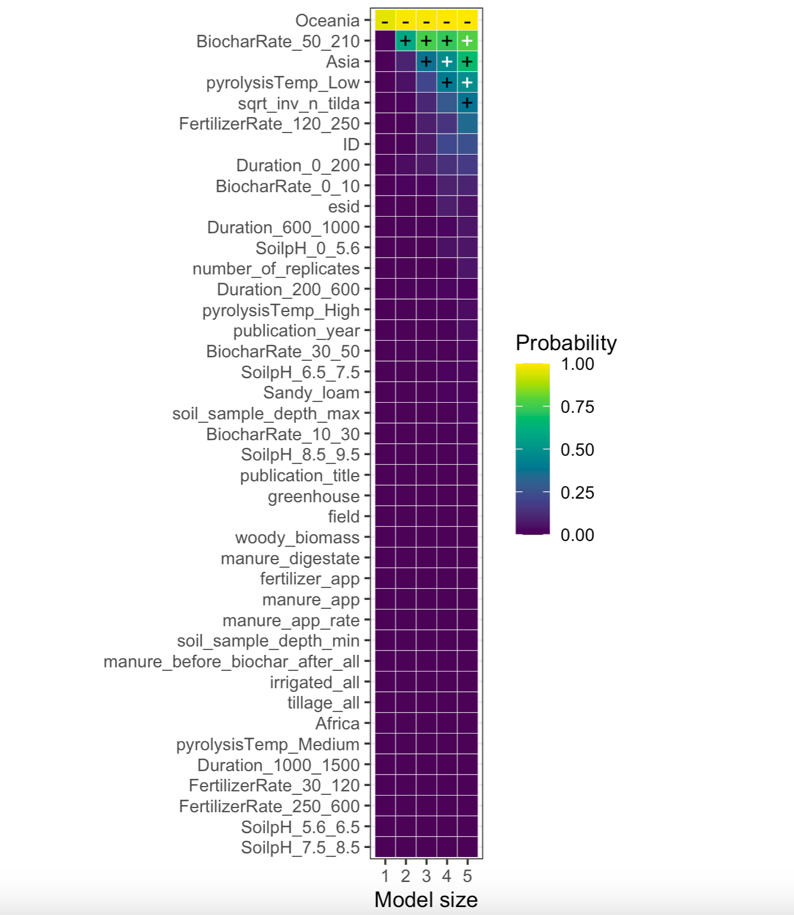
The results of randomized Lasso – Plant nutrient level**

**Figure 12.36.** Predictor selection by randomized Lasso on plant nutrient level

**
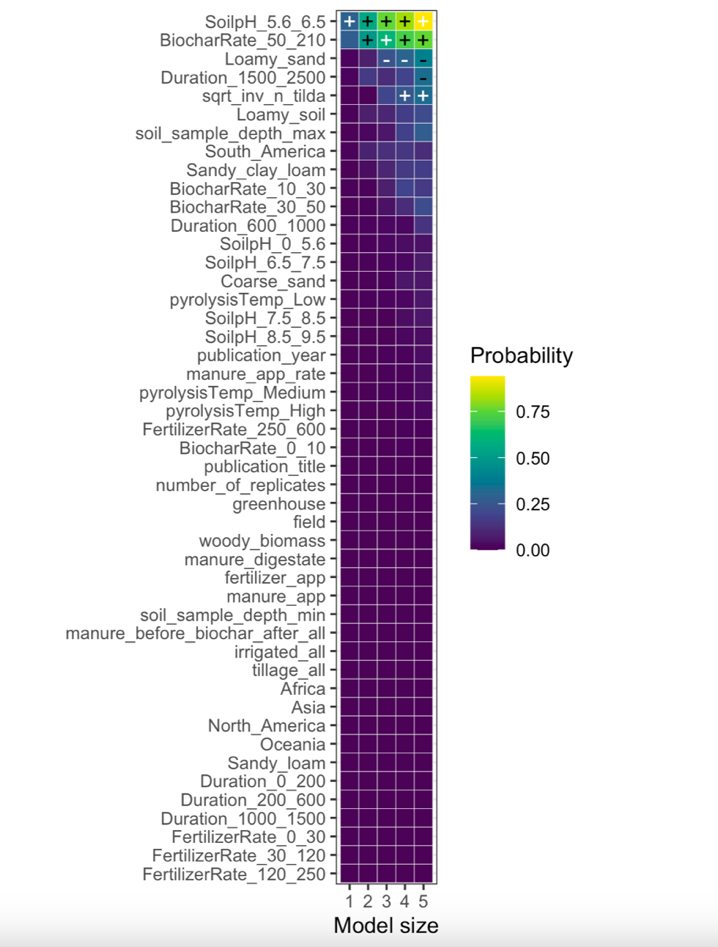
The results of randomized Lasso – N2O emission**

**Figure 12.47.** Predictor selection by randomized Lasso on N2O emission

**
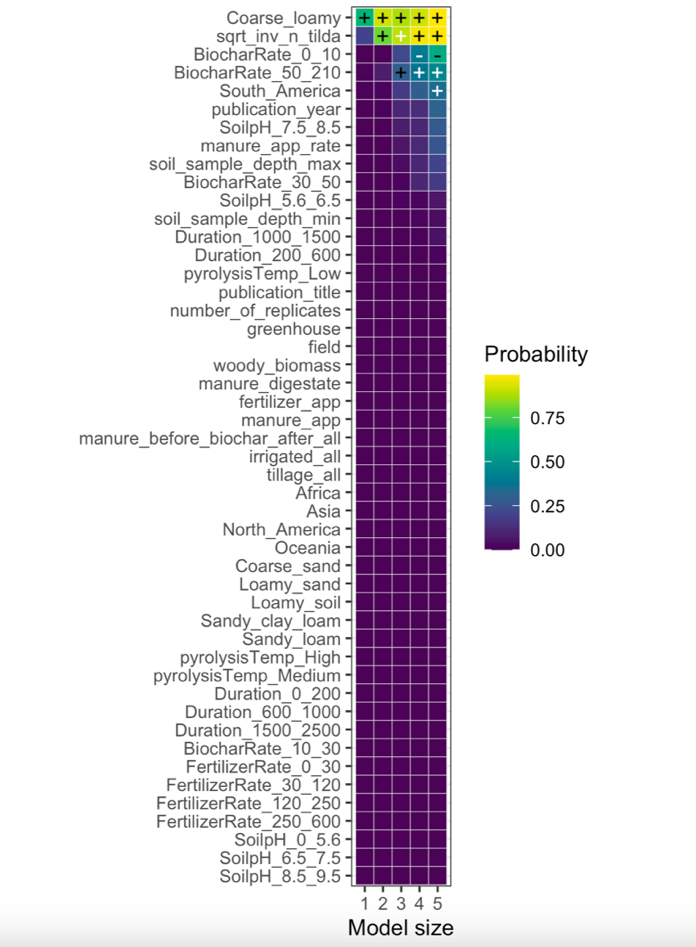
The results of randomized Lasso – Soil NPK availability**

**Figure 12.64.** Predictor selection by randomized Lasso on NPK availability

**The results of randomized Lasso – Potential CEC**

**
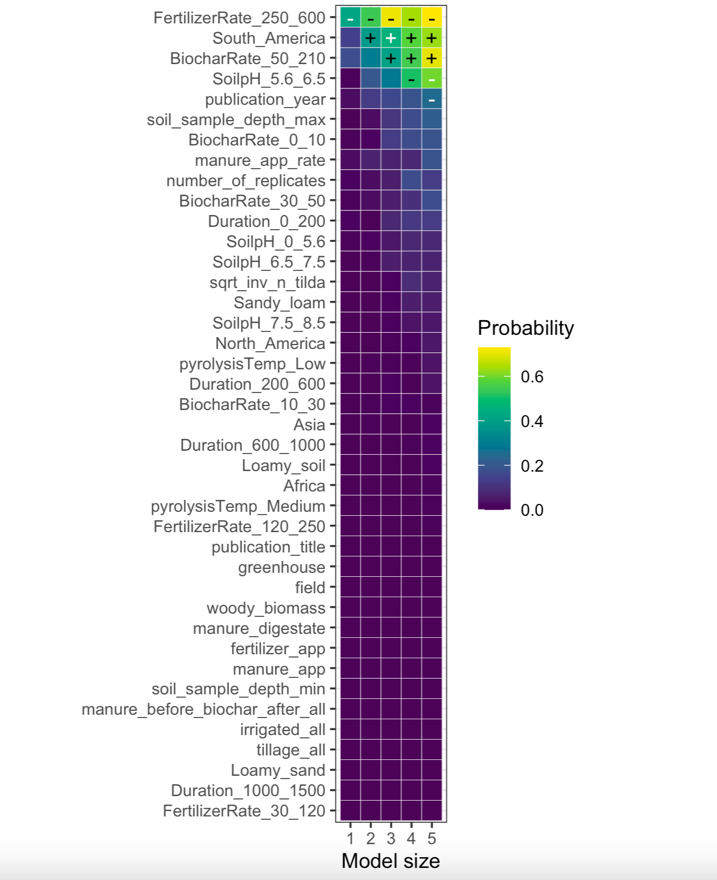
Figure 12.74.** Predictor selection by randomized Lasso on potential CEC

**
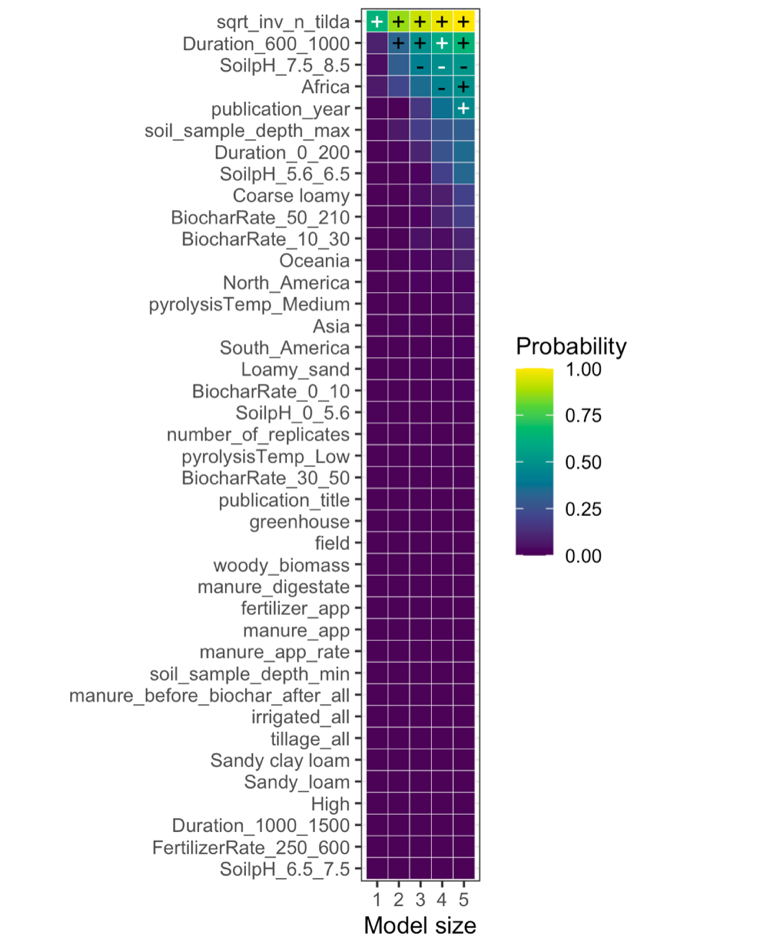
The results of randomized Lasso – Effective CEC**

**Figure 12.82.** Predictor selection by randomized Lasso on effective CEC
