# Supplementary material for: Biochar improves the nutrient cycle in sandy-textured soils and increases crop yield: a systematic review
Source: Environ Evid. 2024 Feb 22;13:3. doi: 10.1186/s13750-024-00326-5 (PMC11376106; doi:10.1186/s13750-024-00326-5)
Supplement: Supplementary file 13 — Additional file 13. Correction for publication bias. Contains funnel plots for each SPP and PET-PEESE results. [file 13750_2024_326_MOESM13_ESM.docx]

**Correction for publication bias**

We corrected for publication bias (PB) for each SPP using the PET (precision effect test) and PEESE (standard error precision effect estimates) methods. This method is especially popular when log response ratio (LnRR) is the effect size (1) (2). PET-PEESE deals with small study effects that might be the potential indicators of PB. The PET method is based on a following model where the study’s effect size is regressed on its standard error (SE):

|  | $\theta_{k}= \beta_{0}+ \beta_{1}{SE}_{\theta k}$ | (1) |
| --- | --- | --- |

We used the square root of the inverse of effective sample size ($\sqrt{1/\tilde{n_{i}}})$ instead of SE because it is recommended for the studies where the effect size is calculated based on response rate (3).

|  | $\theta_{k}= \beta_{0}+ \beta_{1}\sqrt{1/\tilde{n_{i}}}$ | (2) |
| --- | --- | --- |

The square root of the inverse of the effective sample size is calculated based on the following formula

|  | ($\sqrt{1/\tilde{n_{i}}}= \sqrt{n_{1i}{+ n}_{2i}/n_{1i}n_{2i}}$ | (3) |
| --- | --- | --- |

$n_{1i}$ is the sample size of the control, while $n_{2i}$ is the sample size of treatment. The reason for using this formula instead of SE is that SE can be correlated with point estimates because the variance of response ratio comprises both the control and treatment, which are also comprised of point estimates. This can lead to funnel asymmetry. When the true effect seized from $\beta_{0}$ is zero or not significant in equation (2), then the PET method works the best because the PET method ensures that the probability of incorrectly rejecting the null hypothesis (i.e., concluding that there is a significant effect) is controlled at a nominal significance level of 0.05.

PEESE is also regressed in a similar way as PET, but the difference is that instead of SE (in our case ($\sqrt{1/\tilde{n_{i}}})$), the squared SE i.e. the variance (in our case ($1/\tilde{n_{i}}$) (3)) is used as the predictor. This is because small studies are more susceptible to reporting highly over-estimated effects (2). In contrast to PET, PEESE works best when $\beta_{0}$ is not zero and significant.

PET–PEESE is usually performed with a fixed effects (FE) model, mainly because the point estimates are assumed to be biased, and FE can capture unbiased estimates in such a case. No evidence for publication bias (PB) is observed when the point estimates are unbiased, in which case a multilevel random effects model (ML-REML) is the most appropriate approach (2). Thus, the results below show that we applied both models with PET-PEESE to see which one is best suited for the correction of PB.

Funnel plots for each SPP were also created to observe visual funnel asymmetry and the existence of publication bias. Results for PET-PEESE and funnel plots are provided with and without outliers.

**Table 13.1**. Correction for PB in soil total NPK response to biochar application. Results for **full sample**. “ML” - Multilevel model, “FE” – Fixed effects model, “PET – ML” – PET with Multilevel model, “PET- FE” – PET with Fixed effects model, “PEESE – ML” – PEESE with Multilevel model, “PEESE- FE” – PEESE with Fixed effects model. Standard errors (SE) are given in parentheses.

|  | **ML** | **FE** | **PET - ML** | **PET - FE** | **PEESE - ML** | **PEESE - FE** |
| --- | --- | --- | --- | --- | --- | --- |
| Estimate | 0.2808  (0.0501) | 0.1169  (0.0432) | 0.2392  (0.0596) | 0.1596  (0.0438) | 0.2778  (0.0534) | 0.1277  (0.0420) |
| p-Value | <.0001 | 0.0267 | 0.0002 | 0.0025 | <.0001 | 0.0097 |
| Square root of effective sample size | - | - | 0.0147  (0.0117) | -0.0160  (0.0138) | - | - |
| p-value | - | - | 0.2434 | 0.2755 | - | - |
| Effective sample size | - | - | - | - | 0.0002  (0.0005) | -0.0012  (0.0009) |
| p-value | - | - | - | - | 0.7635 | 0.2221 |

**Table 13.2.** Correction for PB in soil total NPK response to biochar application. Results for sample **without outliers**. “ML” - Multilevel model with REML, “FE” – Fixed effects model, “PET – ML” – PET with Multilevel model, “PET- FE” – PET with Fixed effects model, “PEESE – ML” – PEESE with Multilevel model, “PEESE- FE” – PEESE with Fixed effects model. Standard errors (SE) are given in parentheses.

|  | **ML** | **FE** | **PET - ML** | **PET - FE** | **PEESE - ML** | **PEESE - FE** |
| --- | --- | --- | --- | --- | --- | --- |
| Estimate | 0.2914  (0.0528) | 0.2000  (0.0901) | 0.2497  (0.0624) | 0.2278  (0.1005) | 0.2888  (0.0563) | 0.2152  (0.0981) |
| p-Value | <.0001 | 0.0458 | 0.0002 | 0.0365 | <.0001 | 0.0453 |
| Square root of effective sample size | - | - | 0.0145  (0.0119) | -0.0104  (0.0085) | - | - |
| p-value | - | - | 0.2535 | 0.2509 | - | - |
| Effective sample size | - | - | - | - | 0.0001  (0.0005) | -0.0013  (0.0009) |
| p-value | - | - | - | - | 0.8002 | 0.1968 |

Summary: The funnel plot (Figure 1) suggests some asymmetry towards positive effects, but this is not captured in the meta-regression models. PB correction has almost no impact on average treatment effects (ATE). Outlier removal makes FE models non-significant

**
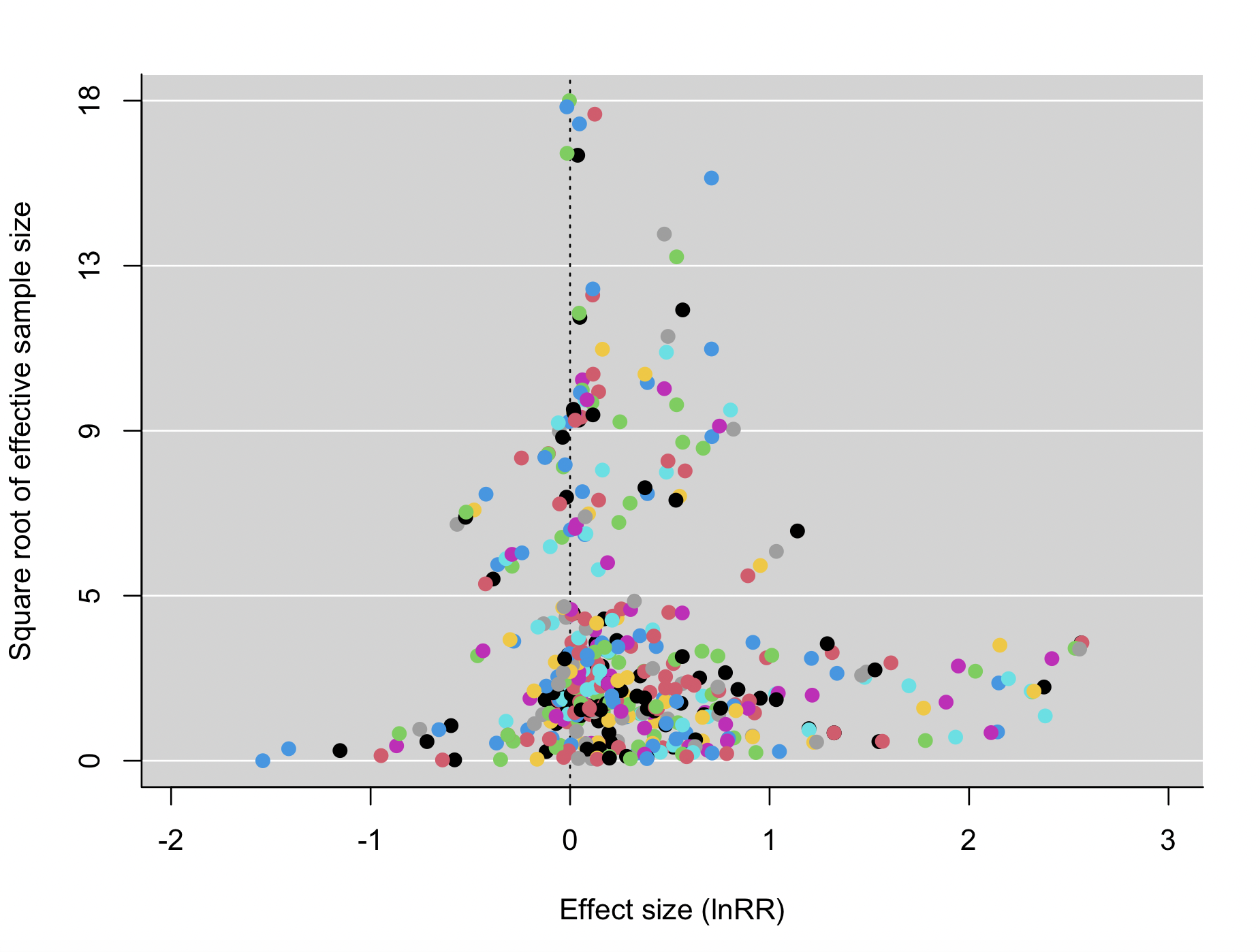
**

**Figure 13.1.** Funnel plot of soil total NPK (Full sample).

**Table 13.3.** Correction for PB in soil mineral nitrogen response to biochar application. Results for the **full sample**. “ML” - Multilevel model with REML, “FE” – Fixed effects model, “PET – ML” – PET with Multilevel model, “PET- FE” – PET with Fixed effects model, “PEESE – ML” – PEESE with Multilevel model, “PEESE- FE” – PEESE with Fixed effects model. Standard errors (SE) are given in parentheses.

|  | **ML** | **FE** | **PET - ML** | **PET - FE** | **PEESE - ML** | **PEESE - FE** |
| --- | --- | --- | --- | --- | --- | --- |
| Estimate | 0.0336  (0.0641) | 0.1174  (0.0056) | 0.1626  (0.0875) | 0.1688  (0.2656) | 0.0560  (0.0670) | 0.1312  (0.0199) |
| p-Value | 0.6041 | 0.0302 | 0.0750 | 0.6101 | 0.4099 | 0.0028 |
| Square root of effective sample size | - | - | -0.0058  (0.0033) | -0.0083  (0.0417) | - | - |
| p-value | - | - | 0.1270 | 0.8663 | - | - |
| Effective sample size | - | - | - | - | -0.00002  (0.00001) | -0.0003  (0.0004) |
| p-value | - | - | - | - | 0.3608 | 0.4154 |

**Table 13.4.** Correction for PB in soil mineral nitrogen response to biochar application. The results below are still **without outliers**. “ML” - Multilevel model with REML, “FE” – Fixed effects model, “PET – ML” – PET with Multilevel model, “PET- FE” – PET with Fixed effects model, “PEESE – ML” – PEESE with Multilevel model, “PEESE- FE” – PEESE with Fixed effects model. Standard errors (SE) are given in parentheses.

|  | **ML** | **FE** | **PET - ML** | **PET - FE** | **PEESE - ML** | **PEESE - FE** |
| --- | --- | --- | --- | --- | --- | --- |
| Estimate | 0.0304  (0.0643) | -0.0461  (0.0583) | 0.1546  (0.0872) | 0.0923  (0.0841) | 0.0521  (0.0674) | -0.0151  (0.0457) |
| p-Value | 0.6395 | 0.4632 | 0.0883 | 0.3390 | 0.4451 | 0.7550 |
| Square root of effective sample size | - | - | -0.0055  (0.0032) | -0.0161  (0.0120) | - | - |
| p-value | - | - | 0.1313 | 0.2077 | - | - |
| Effective sample size | - | - | - | - | -0.00002  (0.00001) | -0.0001  (0.0001) |
| p-value | - | - | - | - | 0.3608 | 0.3825 |

Summary: Relatively symmetric funnel plot (Figure 2). No indication of ATE or PB


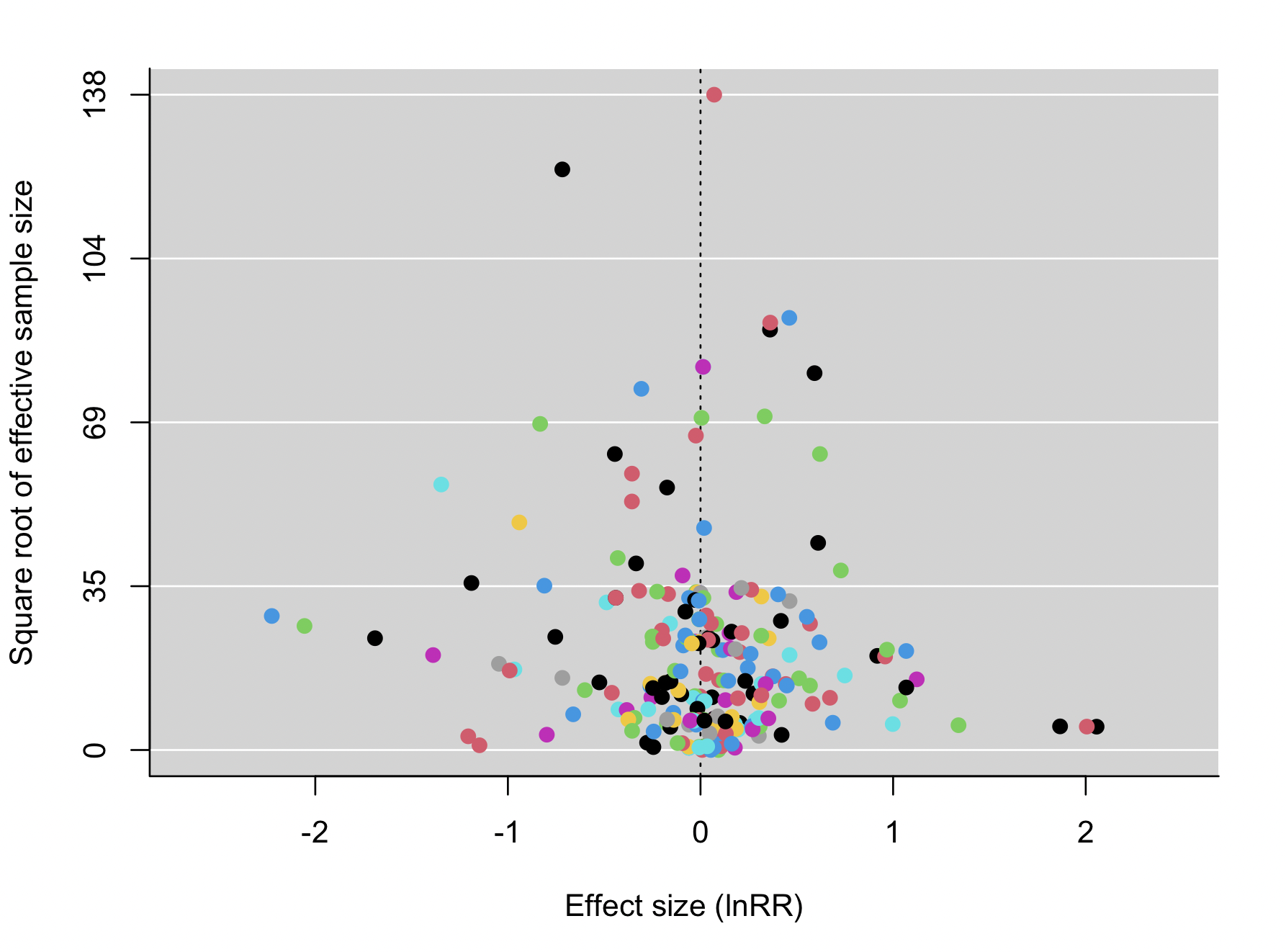


**Figure 13.2.** Funnel plot of soil mineral nitrogen (Full sample).

**Table 13.5.** Correction for PB in plant nutrient level response to biochar application. Results for the **full sample**. “ML” - Multilevel model with REML, “FE” – Fixed effects model, “PET – ML” – PET with Multilevel model, “PET- FE” – PET with Fixed effects model, “PEESE – ML” – PEESE with Multilevel model, “PEESE- FE” – PEESE with Fixed effects model. Standard errors (SE) are given in parentheses.

|  | **ML** | **FE** | **PET - ML** | **PET - FE** | **PEESE - ML** | **PEESE - FE** |
| --- | --- | --- | --- | --- | --- | --- |
| Estimate | 0.1252  (0.0668) | -0.0730  (0.1450) | 0.1832  (0.0468) | 0.0311  (0.0954) | 0.1677  (0.0460) | -0.0118  (0.1036) |
| p-Value | 0.0830 | 0.6483 | 0.0022 | 0.7696 | 0.0029 | 0.9163 |
| Square root of effective sample size | - | - | -0.0149  (0.0164) | -0.0461  (0.0155) | - | - |
| p-value | - | - | 0.4607 | 0.1455 | - | - |
| Effective sample size | - | - | - | - | -0.0010  (0.0013) | -0.0035  (0.0008) |
| p-value | - | - | - | - | 0.5427 | 0.1101 |

**Table 13.6.** Correction for PB in plant nutrient level response to biochar application. “ML” - Multilevel model with REML, “FE” – Fixed effects model, “PET – ML” – PET with Multilevel model, “PET- FE” – PET with Fixed effects model, “PEESE – ML” – PEESE with Multilevel model, “PEESE- FE” – PEESE with Fixed effects model. Standard errors (SE) are given in parentheses.

Summary: The funnel plot (Figure 3) - with three observations “outside the funnel” – is relatively symmetric. ML models suggest ATE, while FE models suggest no ATE. No indication of PB


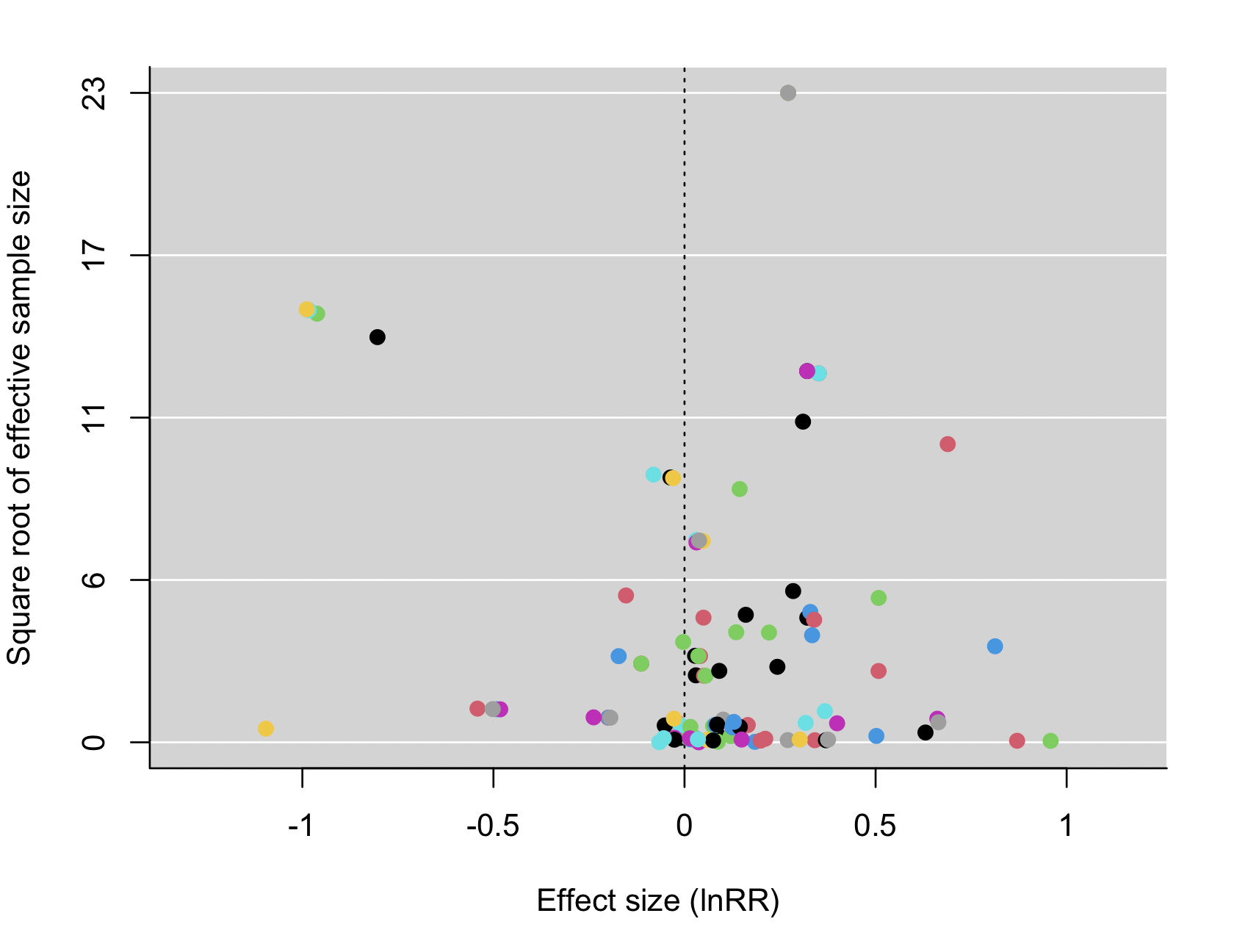


`**Figure 13.3.** Funnel plot of plant nutrient level (Full sample).

**Table 13.7.** Correction for PB in N2O emission response to biochar application. The results below are still **with and without outliers**. “ML” - Multilevel model with REML, “FE” – Fixed effects model, “PET – ML” – PET with Multilevel model, “PET- FE” – PET with Fixed effects model, “PEESE – ML” – PEESE with Multilevel model, “PEESE- FE” – PEESE with Fixed effects model. Standard errors (SE) are given in parentheses.

|  | **ML** | **FE** | **PET - ML** | **PET - FE** | **PEESE - ML** | **PEESE - FE** |
| --- | --- | --- | --- | --- | --- | --- |
| Estimate | -0.3362  (0.1557) | -0.0586  (0.0990) | -0.3678  (0.1854) | -0.0571  (0.1039) | -0.3399  (0.1724) | -0.0581  (0.1021) |
| p-Value | 0.0490 | 0.5816 | 0.0684 | 0.6091 | 0.0695 | 0.5976 |
| Square root of effective sample size | - | - | 0.0078  (0.0226) | -0.0007  (0.0077) | - | - |
| p-value | - | - | 0.7836 | 0.9418 | - | - |
| Effective sample size | - | - | - | - | 0.00003  (0.0004) | -0.00001  (0.0002) |
| p-value | - | - | - | - | 0.9596 | 0.9661 |

Summary: No outliers were detected. Funnel plot (Figure 4) with many imprecise estimates and four precise estimates. ML models suggest some indication for a negative ATE. No indication of PB


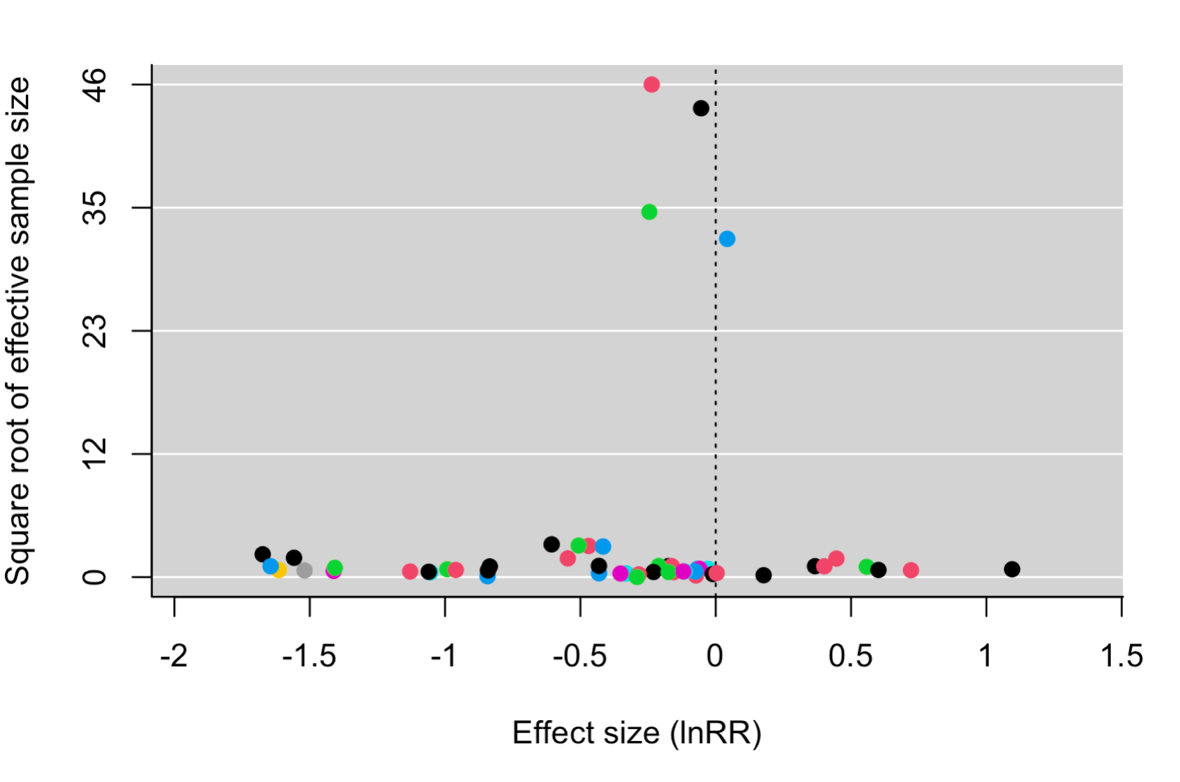


**Figure 13.4.** Funnel plot of N2O emission (Full sample).

**Table 13.8.** Correction for PB in soil NPK availability response to biochar application. Results for the **full sample**. “ML” - Multilevel model with REML, “FE” – Fixed effects model, “PET – ML” – PET with Multilevel model, “PET- FE” – PET with Fixed effects model, “PEESE – ML” – PEESE with Multilevel model, “PEESE- FE” – PEESE with Fixed effects model. Standard errors (SE) are given in parentheses.

|  | **ML** | **FE** | **PET - ML** | **PET - FE** | **PEESE - ML** | **PEESE - FE** |
| --- | --- | --- | --- | --- | --- | --- |
| Estimate | 0.3750  (0.0780) | 0.1573  (0.0850) | 0.3965  (0.0772) | 0.1702 (0.1018) | 0.3838  (0.0785) | 0.1588  (0.0863) |
| p-Value | <.0001 | 0.1160 | <.0001 | 0.1447 | <.0001 | 0.1181 |
| Square root of effective sample size | - | - | -0.0015  (0.0014) | -0.0016  (0.0022) | - | - |
| p-value | - | - | 0.3482 | 0.5321 | - | - |
| Effective sample size | - | - | - | - | -0.000009 (0.000001) | -0.000007 (0.000002) |
| p-value | - | - | - | - | 0.4509 | 0.4363 |

|  | **ML** | **FE** | **PET - ML** | **PET - FE** | **PEESE - ML** | **PEESE - FE** |
| --- | --- | --- | --- | --- | --- | --- |
| Estimate | 0.3788 (0.0805) | 0.1639  (0.0562) | 0.4011  (0.0798) | 0.1798  (0.0633) | 0.3880 (0.0811) | 0.1681  (0.0574) |
| p-Value | <.0001 | 0.0116 | <.0001 | 0.0150 | <.0001 | 0.0115 |
| Square root of effective sample size | - | - | -0.0015  (0.0014) | -0.0015  (0.0009) | - | - |
| p-value | - | - | 0.3446 | 0.2427 | - | - |
| Effective sample size | - | - | - | - | -0.00001 (0.00001) | -0.000007 (0.000001) |
| p-value | - | - | - | - | 0.4507 | 0.2151 |

**Table 13.9.** Correction for PB in soil NPK availability response to biochar application. The results below are still **without outliers**. “ML” - Multilevel model with REML, “FE” – Fixed effects model, “PET – ML” – PET with Multilevel model, “PET- FE” – PET with Fixed effects model, “PEESE – ML” – PEESE with Multilevel model, “PEESE- FE” – PEESE with Fixed effects model. Standard errors (SE) are given in parentheses.

Summary: The funnel plot (Figure 5) suggests asymmetry towards positive effects. ML model indicates ATE. FE models for full sample suggest not ATE but ATE becomes significant without outliers while effect sizes are less than half than for ML models. No indication of PB in regression models.


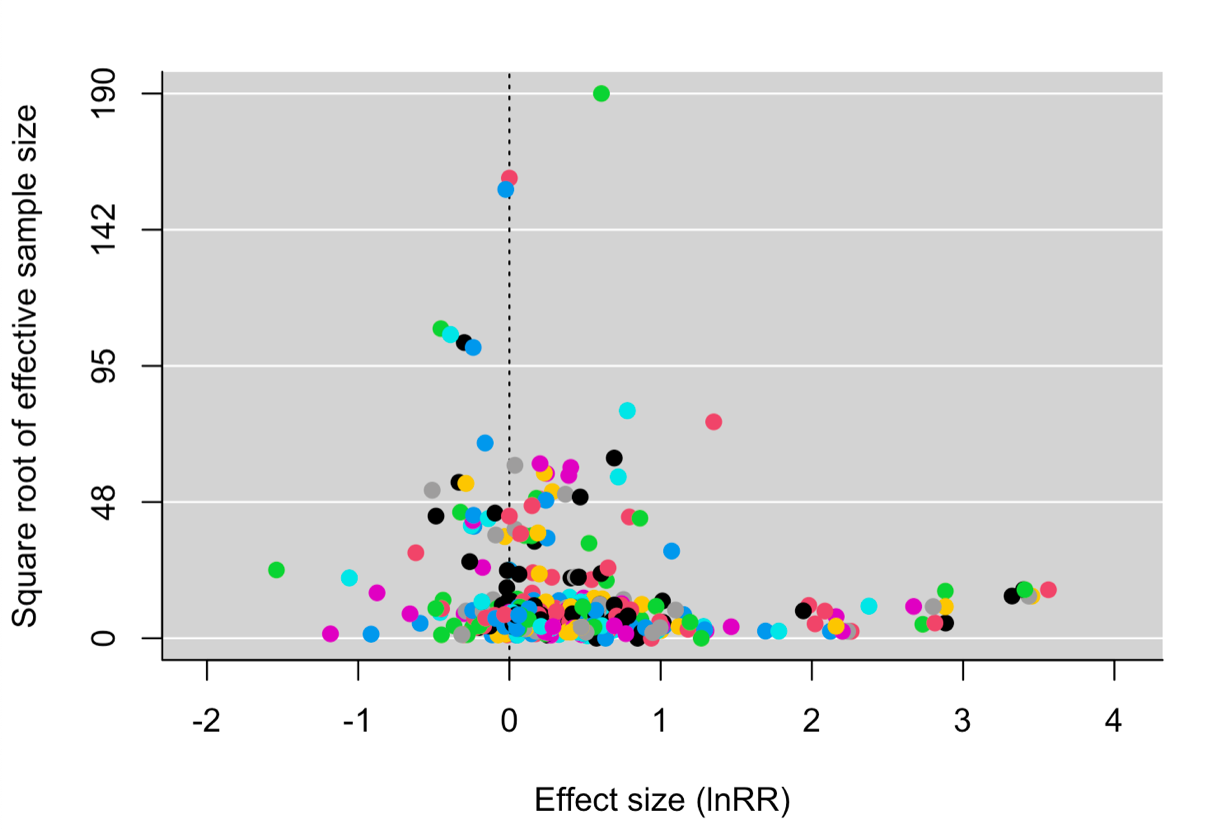


**Figure 13.5.** Funnel plot of soil NPK availability (Full sample)

**Table 13.10.** Correction for PB in soil potential CEC response to biochar application. Results for the **full sample**. “ML” - Multilevel model with REML, “FE” – Fixed effects model, “PET – ML” – PET with Multilevel model, “PET- FE” – PET with Fixed effects model, “PEESE – ML” – PEESE with Multilevel model, “PEESE- FE” – PEESE with Fixed effects model. Standard errors (SE) are given in parentheses.

|  | **ML** | **FE** | **PET - ML** | **PET - FE** | **PEESE - ML** | **PEESE - FE** |
| --- | --- | --- | --- | --- | --- | --- |
| Estimate | 0.1444  (0.0664) | 0.1118  (0.0426) | -0.0925  (0.0961) | -0.0769 (0.0866) | 0.0357  (0.0320) | 0.0357  (0.0320) |
| p-Value | 0.0564 | 0.0712 | 0.3866 | 0.4464 | 0.3492 | 0.3492 |
| Square root of effective sample size | - | - | 0.3760  (0.1558) | 0.3540  (0.1606) | - | - |
| p-value | - | - | 0.1378 | 0.1592 | - | - |
| Effective sample size | - | - | - | - | 0.1944  (0.0724) | 0.1944  (0.0724) |
| p-value | - | - | - | - | 0.1665 | 0.1665 |

**Table 13.11.** Correction for PB in soil potential CEC response to biochar application. The results below are still **without outliers**. “ML” - Multilevel model with REML, “FE” – Fixed effects model, “PET – ML” – PET with Multilevel model, “PET- FE” – PET with Fixed effects model, “PEESE – ML” – PEESE with Multilevel model, “PEESE- FE” – PEESE with Fixed effects model. Standard errors (SE) are given in parentheses.

|  | **ML** | **FE** | **PET - ML** | **PET - FE** | **PEESE - ML** | **PEESE - FE** |
| --- | --- | --- | --- | --- | --- | --- |
| Estimate | 0.1543 (0.0724) | 0.1395 (0.0591) | -0.0794 (0.1123) | -0.0652 (0.0890) | 0.0485 (0.0617) | 0.0512 (0.0423) |
| p-Value | 0.0601 | 0.0667 | 0.5059 | 0.5104 | 0.4578 | 0.2973 |
| Square root of effective sample size | - | - | 0.3810 (0.1617) | 0.3672 (0.1537) | - | - |
| p-value | - | - | 0.1488 | 0.1526 | - | - |
| Effective sample size | - | - | - | - | 0.1931 (0.0753) | 0.1914 (0.0722) |
| p-value | - | - | - | - | 0.1632 | 0.1680 |

Summary: Odd funnel plot (Figure 6). Precise studies with larger effect sizes than imprecise studies. ML suggests ATE but PB correction makes it non-significant but SE is also insignificant. All FE models are non-significant.

**
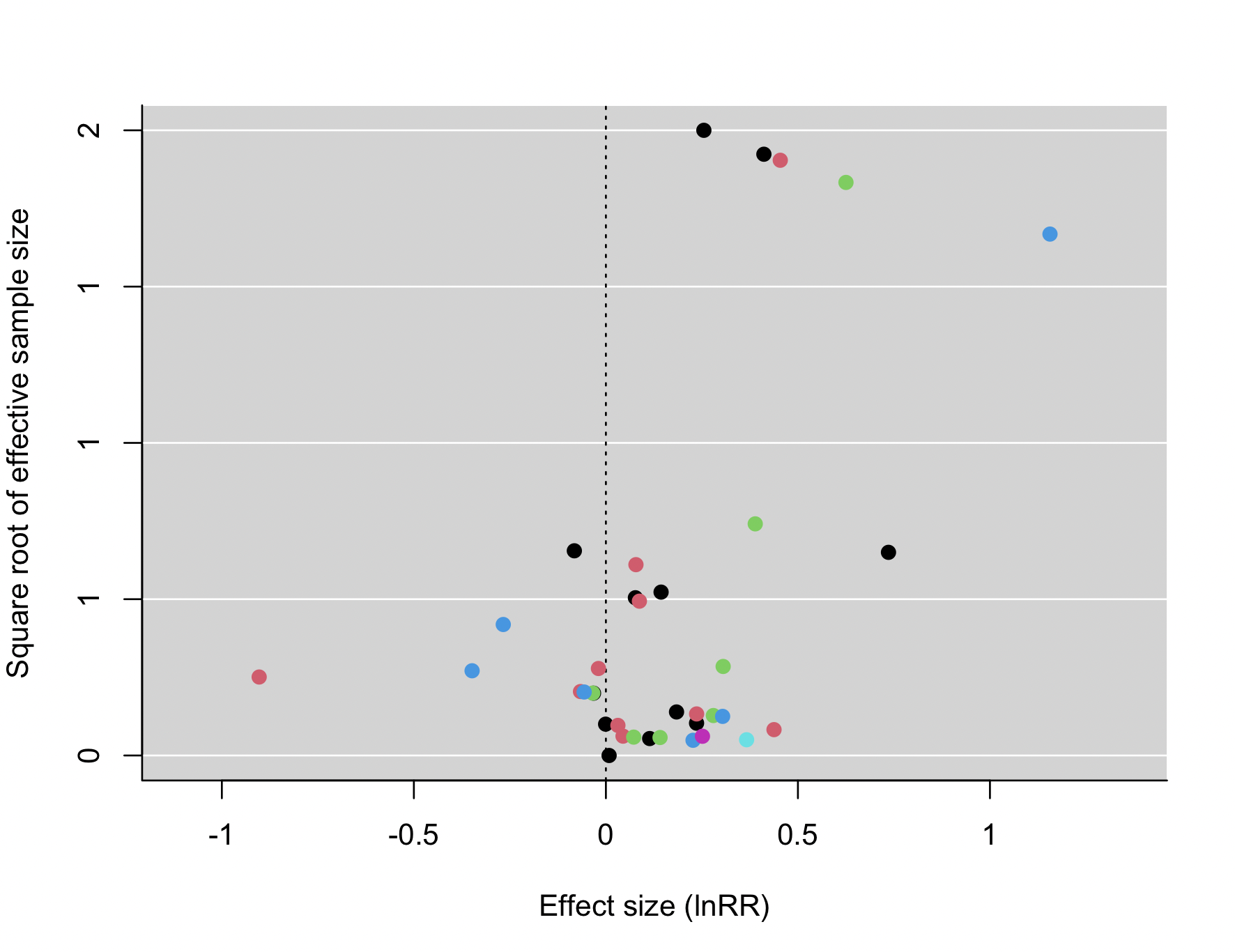
**

**Figure 13.6.** Funnel plot of potential CEC (Full sample).

**Table 13.12.** Correction for PB in soil effective CEC response to biochar application. The results below are still **with and without outliers**. “ML” - Multilevel model with REML, “FE” – Fixed effects model, “PET – ML” – PET with Multilevel model, “PET- FE” – PET with Fixed effects model, “PEESE – ML” – PEESE with Multilevel model, “PEESE- FE” – PEESE with Fixed effects model. Standard errors (SE) are given in parentheses.

|  | **ML** | **FE** | **PET - ML** | **PET - FE** | **PEESE - ML** | **PEESE - FE** |
| --- | --- | --- | --- | --- | --- | --- |
| Estimate | 0.2963 (0.1181) | 0.1448 (0.0737) | -0.4821 (0.2414) | -0.1763 (0.1523) | -0.0281 (0.0714) | -0.0195 (0.0708) |
| p-Value | 0.0278 | 0.1997 | 0.0823 | 0.3483 | 0.7028 | 0.8041 |
| Square root of effective sample size | - | - | 0.1253 (0.0478) | 0.0533 (0.0224) | - | - |
| p-value | - | - | 0.0538 | 0.1442 | - | - |
| Effective sample size | - | - | - | - | 0.0070 (0.0025) | 0.0043 (0.0012) |
| p-value | - | - | - | - | 0.0720 | 0.0741 |

Summary: No outliers are detected. Again, large studies have larger effect sizes. ML suggests ATE but vanishes with PB correction. The first time that PB is significant.

**
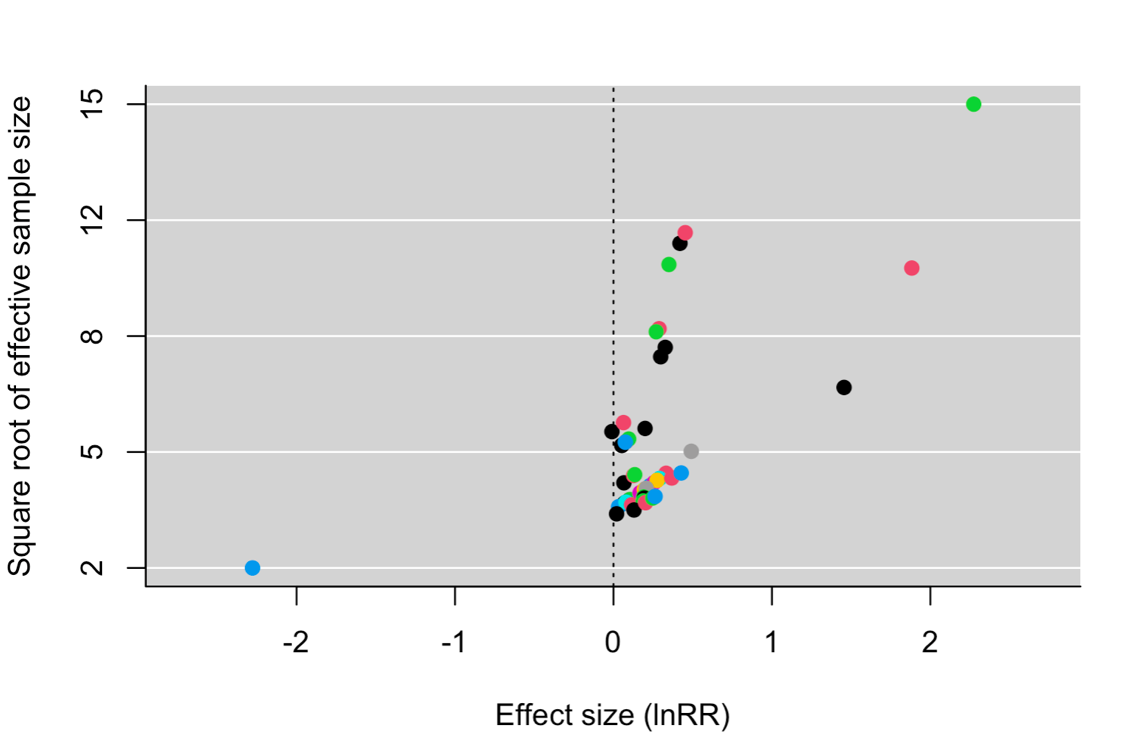
**

**Figure 13.7.** Funnel plot of effective CEC (Full sample).

**Table 13.13.** Correction for PB in NUE response to biochar application. The results below are still **with and without outliers**. “ML” - Multilevel model with REML, “FE” – Fixed effects model, “PET – ML” – PET with Multilevel model, “PET- FE” – PET with Fixed effects model, “PEESE – ML” – PEESE with Multilevel model, “PEESE- FE” – PEESE with Fixed effects model. Standard errors (SE) are given in parentheses.

|  | **ML** | **FE** | **PET - ML** | **PET - FE** | **PEESE - ML** | **PEESE - FE** |
| --- | --- | --- | --- | --- | --- | --- |
| Estimate | -0.0280 (0.0764) | 0.0151 (0.0910) | -0.7105  (0.4079) | -0.3570 (0.1555) | -0.3509 (0.2011) | -0.1688 (0.1495) |
| p-Value | 0.7354 | 0.8836 | 0.2228 | 0.1967 | 0.1836 | 0.3716 |
| Square root of effective sample size | - | - | 0.7407 (0.3706) | 0.4050 (0.1256) | - | - |
| p-value | - | - | 0.1768 | 0.1281 | - | - |
| Effective sample size | - | - | - | - | 0.3051 (0.2166) | 0.1930 (0.1190) |
| p-value | - | - | - | - | 0.3113 | 0.2993 |

Summary: Precise studies with larger effect sizes than imprecise studies (Figure 8). ML suggests no ATE, PB correction makes it non-significant, and SE is also insignificant. All FE models are non-significant.

**
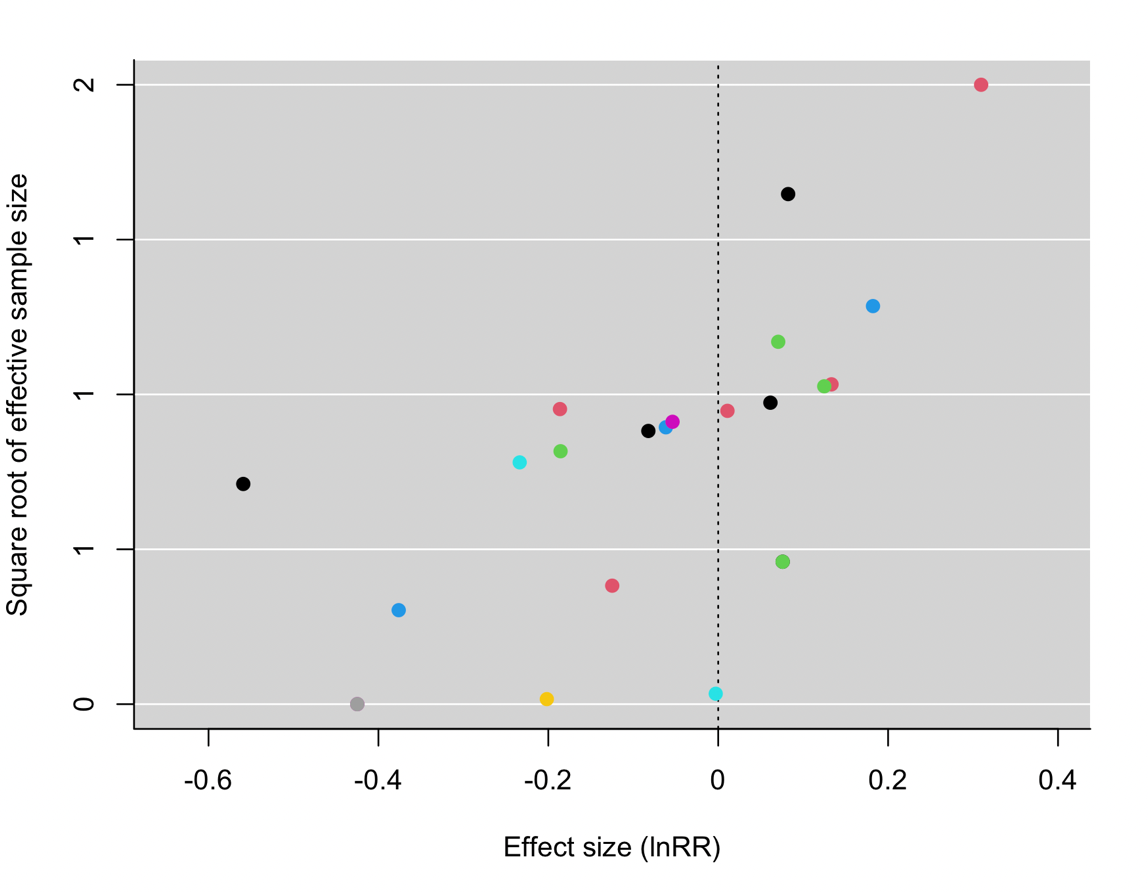
**

**Figure 13.8.** Funnel plot of nutrient use efficiency (Full sample).

**Table 13.15.** Correction for PB in mineral N leaching response to biochar application. The results below are still **with and without outliers**. “ML” - Multilevel model with REML, “FE” – Fixed effects model, “PET – ML” – PET with Multilevel model, “PET- FE” – PET with Fixed effects model, “PEESE – ML” – PEESE with Multilevel model, “PEESE- FE” – PEESE with Fixed effects model. Standard errors (SE) are given in parentheses.

|  | **ML** | **FE** | **PET - ML** | **PET - FE** | **PEESE - ML** | **PEESE - FE** |
| --- | --- | --- | --- | --- | --- | --- |
| Estimate | -0.7364 (0.0730) | -0.7479 (0.0604) | -0.6884 (0.1341) | -0.7369 (0.0916) | -0.7212 (0.0933) | -0.7312 (0.0703) |
| p-Value | 0.0126 | 0.0081 | 0.0317 | 0.0195 | 0.0222 | 0.0112 |
| Square root of effective sample size | - | - | -0.0064 (0.0438) | -0.0071 (0.0354) | - | - |
| p-value | - | - | 0.9029 | 0.8645 | - | - |
| Effective sample size | - | - | - | - | -0.0028 (0.0045) | -0.0027 (0.0038) |
| p-value | - | - | - | - | 0.6230 | 0.5917 |

Summary: the fairly symmetric funnel (Figure 9). Negative ATE in all models


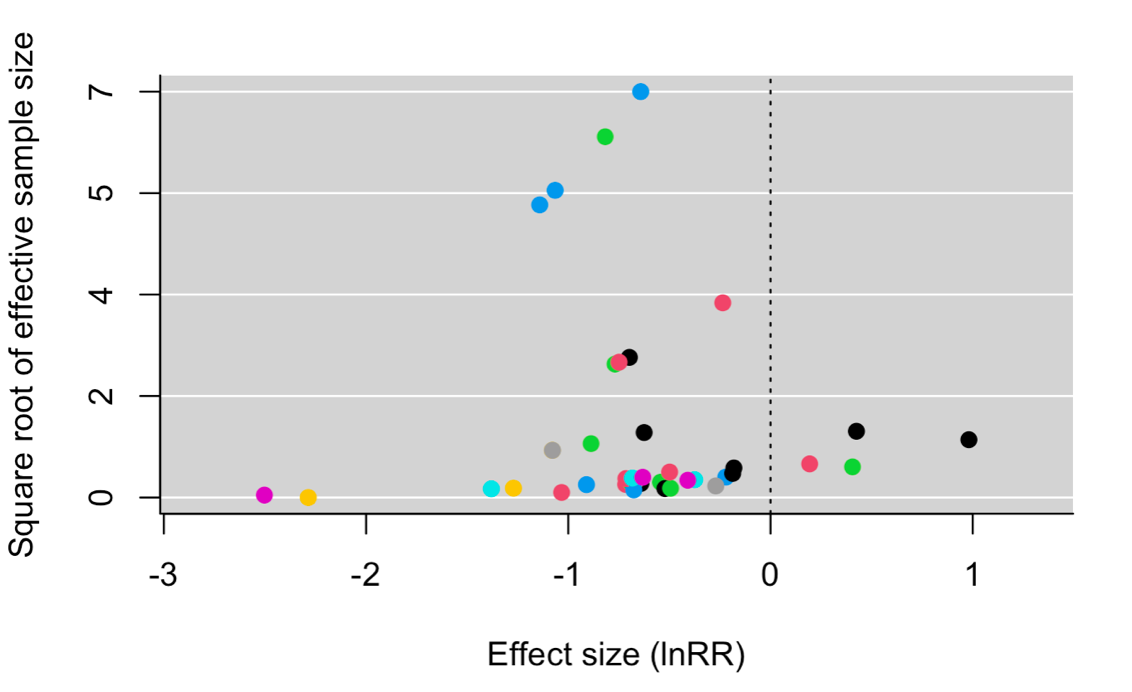


**Figure 13.9.** Funnel plot of mineral N leaching (Full sample).

**Additional to SPPs: correction for publication bias (Food crop yield)**

**Table 13.16.** Correction for PB in food crop yield response to biochar application. Results for the **full sample**. “ML” - Multilevel model with REML, “FE” – Fixed effects model, “PET – ML” – PET with Multilevel model, “PET- FE” – PET with Fixed effects model, “PEESE – ML” – PEESE with Multilevel model, “PEESE- FE” – PEESE with Fixed effects model. Standard errors (SE) are given in parentheses.

|  | **ML** | **FE** | **PET - ML** | **PET - FE** | **PEESE - ML** | **PEESE - FE** |
| --- | --- | --- | --- | --- | --- | --- |
| Estimate | 0.1242 (0.0525) | 0.3711 (0.1942) | -0.0893 (0.1275) | 0.8411 (0.5301) | 0.0525 (0.0631) | 0.6361 (0.2362) |
| p-Value | 0.0315 | 0.2477 | 0.5015 | 0.2309 | 0.4215 | 0.0971 |
| Square root of effective sample size | - | - | 0.0570 (0.0361) | -0.1168 (0.1470) | - | - |
| p-value | - | - | 0.1699 | 0.5171 | - | - |
| Effective sample size | - | - | - | - | 0.0042 (0.0027) | -0.0154 (0.0143) |
| p-value | - | - | - | - | 0.2253 | 0.4085 |

**Table 13.17.** Correction for PB in food crop yield response to biochar application. The results below are still **without outliers**. “ML” - Multilevel model with REML, “FE” – Fixed effects model, “PET – ML” – PET with Multilevel model, “PET- FE” – PET with Fixed effects model, “PEESE – ML” – PEESE with Multilevel model, “PEESE- FE” – PEESE with Fixed effects model. Standard errors (SE) are given in parentheses.

|  | **ML** | **FE** | **PET - ML** | **PET - FE** | **PEESE - ML** | **PEESE - FE** |
| --- | --- | --- | --- | --- | --- | --- |
| Estimate | 0.1610 (0.0727) | 0.1552 (0.0816) | 0.0384 (0.1214) | 0.0339 (0.1389) | 0.0976 (0.0933) | 0.0954 (0.1054) |
| p-Value | 0.0484 | 0.0874 | 0.7616 | 0.8153 | 0.3208 | 0.3896 |
| Square root of effective sample size | - | - | 0.0312 (0.0230) | 0.0315 (0.0249) | - | - |
| p-value | - | - | 0.2614 | 0.2946 | - | - |
| Effective sample size | - | - | - | - | 0.0032 (0.0026) | 0.0033 (0.0027) |
| p-value | - | - | - | - | 0.3541 | 0.3715 |

Summary: The funnel plot (Figure 10) indicates a slight asymmetry toward positive effects. ML suggests ATE, PB correction makes it non-significant, and SE is also insignificant. All FE models are non-significant.


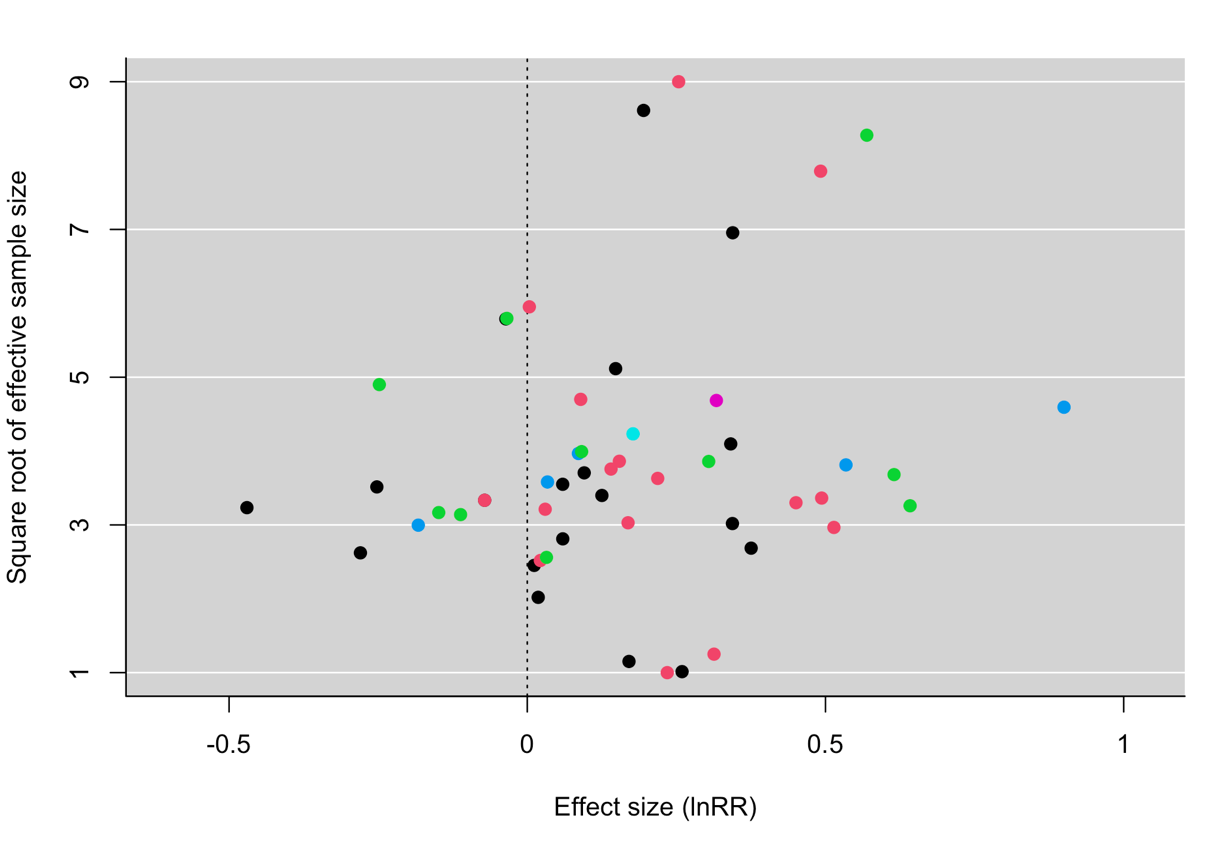


**Figure 13.10.** Funnel plot of Food crop yield (Full sample).

**Additional to SPPs: correction for publication bias (Biomass production)**

**Table 13.18.** Correction for PB in biomass production response to biochar application. The results below are **with and** **without outliers**. “ML” - Multilevel model with REML, “FE” – Fixed effects model, “PET – ML” – PET with Multilevel model, “PET- FE” – PET with Fixed effects model, “PEESE – ML” – PEESE with Multilevel model, “PEESE- FE” – PEESE with Fixed effects model. Standard errors (SE) are given in parentheses.

|  | **ML** | **FE** | **PET - ML** | **PET - FE** | **PEESE - ML** | **PEESE - FE** |
| --- | --- | --- | --- | --- | --- | --- |
| Estimate | -0.0810 (0.0455) | -0.1301 (0.1224) | -0.1744 (0.0550) | -0.2615 (0.0177) | -0.1296 (0.0346) | -0.2090 (0.0448) |
| p-Value | 0.1701 | 0.4263 | 0.1273 | 0.0358 | 0.0724 | 0.1157 |
| Square root of effective sample size | - | - | 0.0232 (0.0108) | 0.0454 (0.0137) | - | - |
| p-value | - | - | 0.1589 | 0.0603 | - | - |
| Effective sample size | - | - | - | - | 0.0015 (0.0013) | 0.0039 (0.0019) |
| p-value | - | - | - | - | 0.3827 | 0.1846 |

Summary: No outliers are detected. Large studies tend to exhibit larger effect sizes, as indicated by the results (Figure 11). The meta-analysis suggests no ATE, and the existing effect diminishes after applying PB correction. Interestingly, no evidence of PB is detected in the analysis.


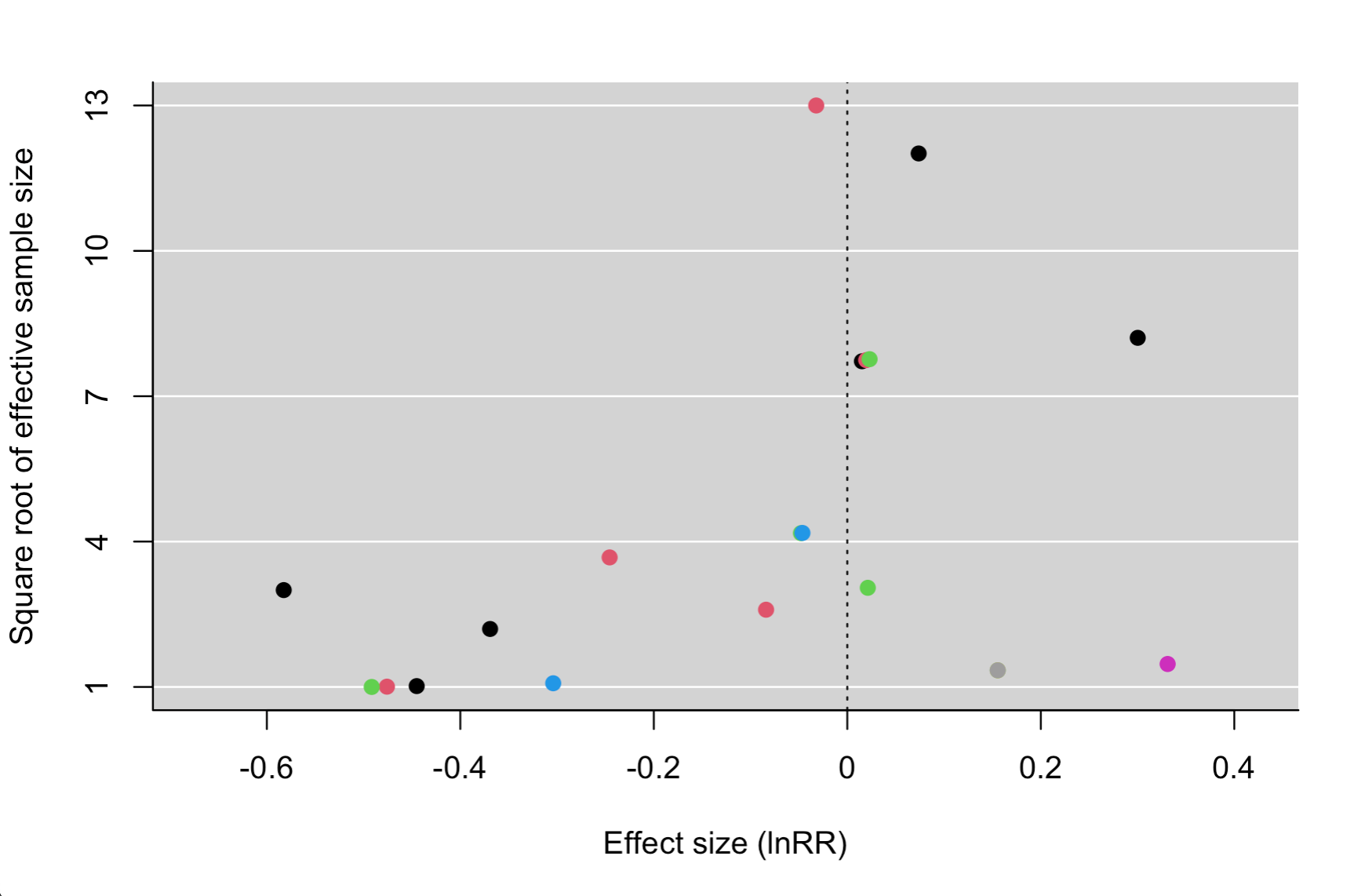
**Figure 13.11.** Funnel plot of Biomass production (Full sample)

**Reference list**

1. Stanley TD, Doucouliagos H. Meta‐regression approximations to reduce publication selection bias. Research Synthesis Methods. 2014;5(1):60-78.

2. Harrer M, Cuijpers P, Furukawa TA, Ebert DD. Doing meta-analysis with R: A hands-on guide: CRC press; 2021.

3. Nakagawa S, Lagisz M, Jennions MD, Koricheva J, Noble DW, Parker TH, et al. Methods for testing publication bias in ecological and evolutionary meta‐analyses. Methods in Ecology and Evolution. 2022;13(1):4-21.
